# PROTACs in cancer therapy: targeted degradation of GPX4, PARP and epigenetic regulators

**DOI:** 10.1080/14756366.2026.2636394

**Published:** 2026-03-26

**Authors:** Sunny Periyasamy, Thyla Jarrett, Joe Truong, Rachid Skouta

**Affiliations:** ^a^ Department of Chemistry, University of Massachusetts, Amherst, MA, USA; ^b^ Department of Biology, University of Massachusetts, Amherst, MA, USA

**Keywords:** Chemical synthesis of anticancer compounds, targeted protein degradation (TPD), proteolysis targeting chimaera (PROTAC), GPX4, epigenetic regulators

## Abstract

The degradation of overexpressed proteins has emerged as a promising strategy for halting disease progression, particularly in cancer. Traditional small-molecule drugs often face limitations in the elimination of pathogenic proteins, leading to the development of targeted protein degradation (TPD) approaches. A prominent strategy for TPD is the proteolysis targeting chimaera (PROTAC) which harnesses the ubiquitin proteasome system, the cell’s innate degradation machinery, to degrade proteins of interest (POIs). In this review, we will focus on the design and synthetic strategies that led the advancements of PROTACs as a cancer therapy for the targeted degradation of poly ADP-ribose polymerases (PARPs), glutathione peroxidase 4 (GPX4) and epigenetic regulators. We also aim to address the prevailing challenges in PROTAC development and clinical translation, namely target diversification, oral bioavailability, stability, degradation efficiency, and optimising multivalent binding.

## Introduction

Cancer remains among the most fatal diseases, with around 20 million new cancer cases reported around the world in recent years. According to the National Cancer Institute, the cancers responsible for over 50% of deaths are breast, prostate, lung, bronchus and colorectal cancers, with breast cancer being the leading cause of new cancer cases.^1^ Over 100 chemotherapy agents are on the market that can be categorised into alkylating agents, anti-metabolites, antibiotics, topoisomerase inhibitors and mitotic inhibitors.^2^ A commonality between many of these existing chemotherapeutics are the range of adverse side effects they exhibit on patients, often due to off-target toxicity of such drugs in healthy cells.^3^^,^^4^ The drawbacks of cancer drugs, particularly treatments intended to inhibit disease-associated proteins, prompt the development of novel targeted protein degradation approaches yielding reduced toxicity and higher efficacy with sub-stoichiometric dosages.

### Proteolysis targeting Chimaeras (PROTACs)

**Figure 1.**
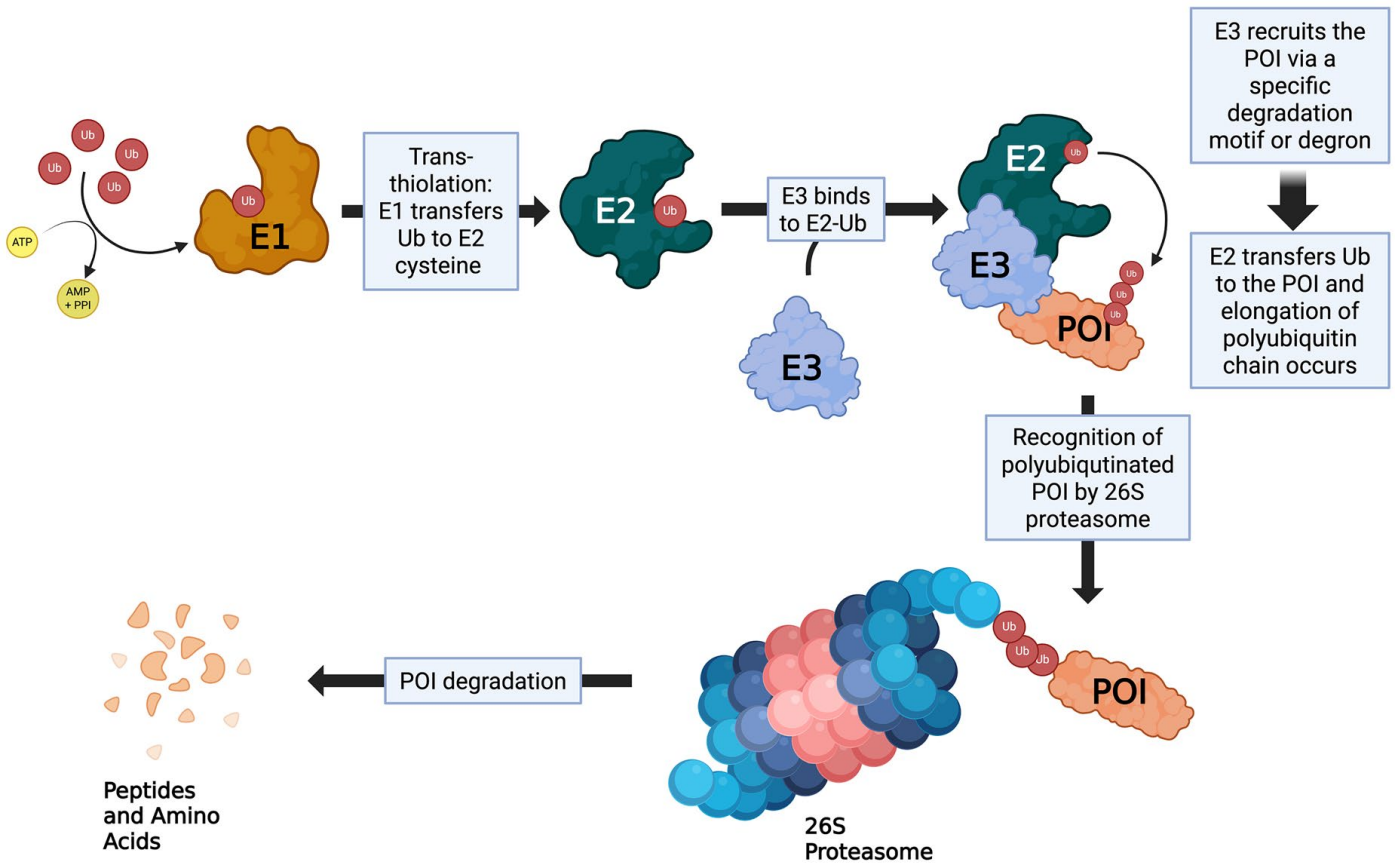
This schematic illustrates the stepwise process of protein degradation via the ubiquitin proteasome system. The ATP-dependent activation of ubiquitin by the ubiquitin-activating enzyme (E1) is followed by the transfer of ubiquitin from E1 to the ubiquitin-conjugating enzyme (E2). Subsequently, the ubiquitin ligase (E3) binds to the POI and E2, forming a ternary complex and enabling the transfer of ubiquitin from E2 to the POI. Following the addition of a polyubiquitin chain to the POI, recognition of the POI by the proteasome occurs. The POI is processed by the proteasome complex, which unfolds, translocates and degrades this protein into the constituent peptides and amino acids. It is understood that this process is essential for the quality control of proteins, regulated protein turnover and numerous cellular processes. (created via BioRender, https://app.biorender.com/, on 29 April 2025).

PROTACs (Proteolysis Targeting Chimaeras) have emerged as a novel and promising approach to treating various forms of cancer through leveraging the innate protein degradation tools, namely the ubiquitin proteasome system, that is present in mammalian cells. The general structure of this therapeutic can be described as a heterobifunctional molecule, consisting of a ligand for the protein of interest (POI) linked to a ligand for the enzyme E3 ligase. The strategic recruitment of E3 ligases represents a fundamental component of PROTAC-mediated TPD, wherein these ligases operating in conjunction with the E1 and E2 enzymes of the UPS orchestrate the polyubiquitination of target proteins, thereby marking them for degradation by the 26S proteasome (Figure 1).^5^ This class of therapeutic has been highly investigated in recent years due to the advantages of the ‘event-driven-pharmacology’ approach that PROTACs hold, which involves the degradation of the protein of interest that occurs upon interaction with both the protein of interest and E3 ligase, differentiating PROTACs from the stoichiometric dose dependency associated with traditional inhibitors.^6^

### The emergence of PROTACs: from discovery to clinical trials

**Figure 2.**
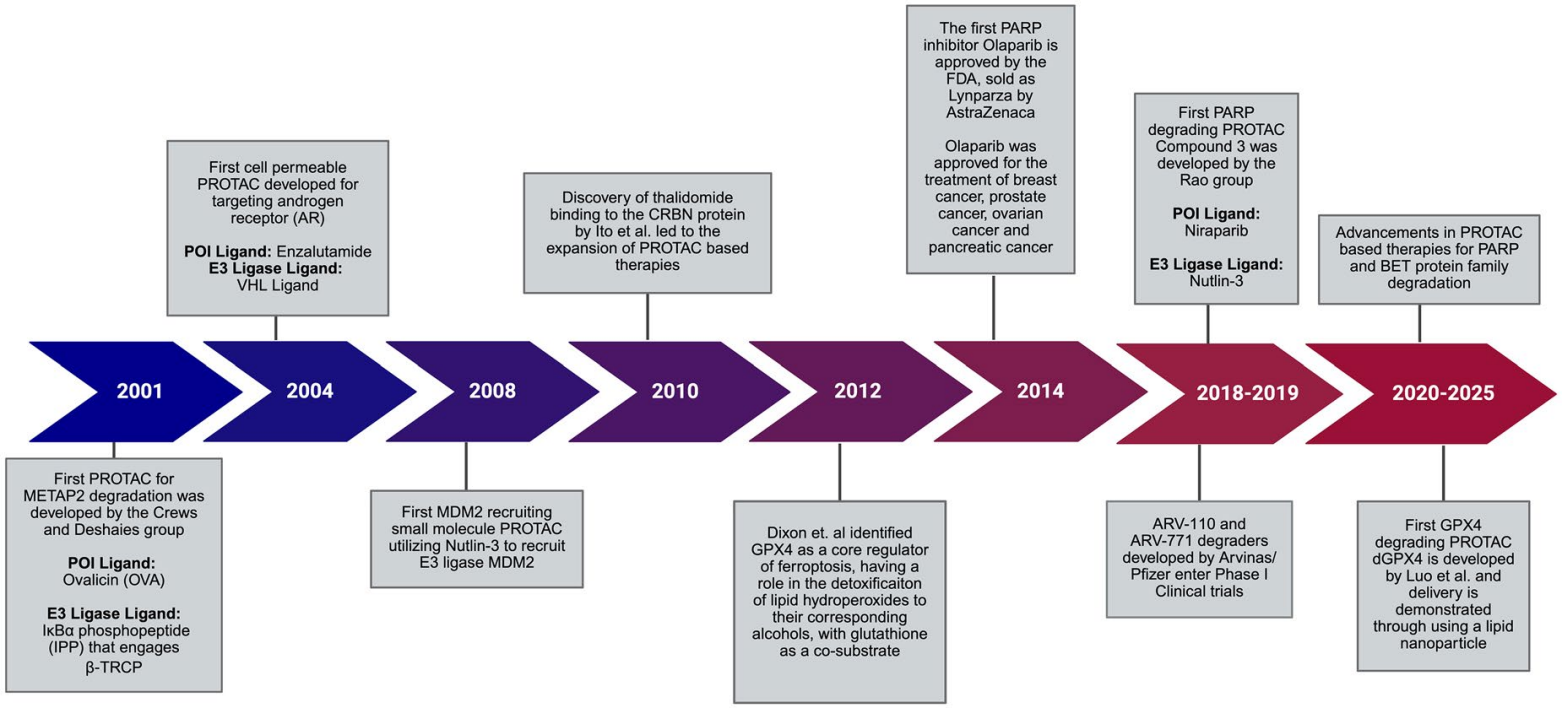
Timeline of PROTAC Development (2001–2025). Chronological overview of key milestones in the evolution of PROTAC technology from 2001 to 2025. This timeline highlights the conceptual development of PROTACs, advances in their clinical translation and the emergence of PROTACs targeting clinically relevant proteins such as PARP and the bromo and extra-terminal domain (BET) family. Special emphasis is placed on the discovery of the role of GPX4 in ferroptosis and the development of small molecule inhibitors and degraders targeting this pathway.^7–13^ (created via BioRender, https://app.biorender.com/, on 29 April 2025).

The first PROTAC was reported by Sakamoto et al. in 2001 through the development of Protac-1, a bifunctional molecule that tethered methionine aminopeptidase-2 (MetAP-2) to the SCFβ-TRCP ubiquitin ligase complex for targeted protein degradation (Figure 2).^7^ Initial approaches to designing PROTACs involved a peptide-based design, while recent innovations employ small-molecule-based PROTAC designs for enhanced cell permeability.^14^ PROTAC designs ventured into small molecule-based strategies following the work of Schneekloth et al. towards the development of a cell-permeable small molecule PROTAC capable of degrading androgen receptor (AR) through recruiting the E3 ligase MDM2.^8^ In later years, Buckley et al. reported the first small molecule targeting the Von-Hippel Lindau protein (VHL) with the aim of mimicking the binding of the known VHL substrate hypoxia-inducible factor 1-alpha (HIF-1α).^15^ Hydroxyproline served as the foundational scaffold for the design of the VHL ligand, leveraging insights from the known interactions of the Hyp564 residue with the native VHL binding interface to facilitate ligand optimisation. The usage of *in silico* design strategies along with structure-guided medicinal chemistry enabled the successful development of this small molecule ligand and facilitated progress in the design of cell-penetrant molecules that would be the basis of design for subsequent PROTAC variants. Advancements have been made in translating PROTACs to the clinic, with approximately 20 PROTACs in the clinical stage as of 2023.^16^

### PROTACS in clinical trials and their significance

**Figure 3.**
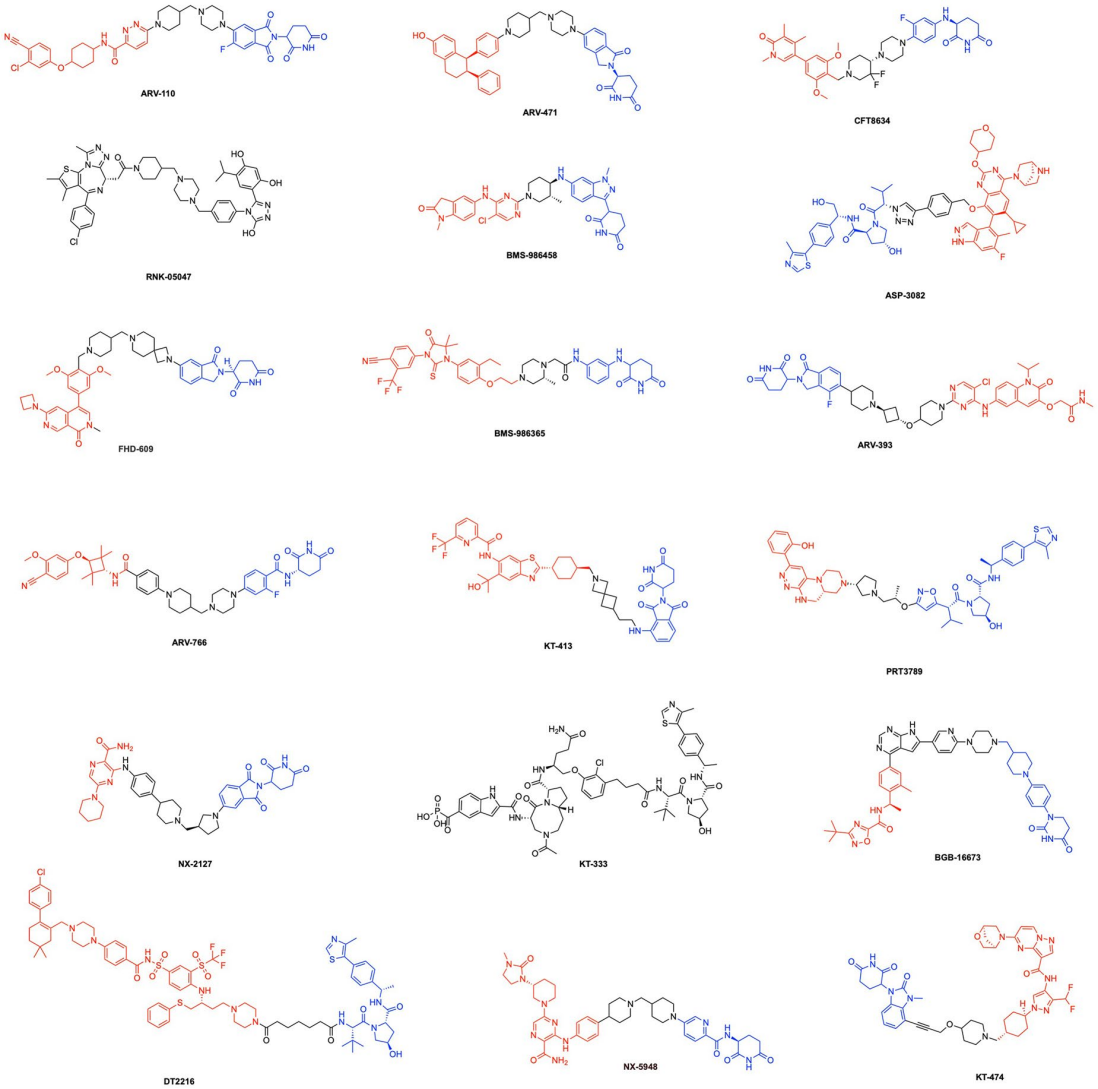
Structures of various PROTAC molecules currently in clinical trials. For PROTACs with publicly available information on the E3 ligase ligand and the POI ligand, the E3 ligase ligand is highlighted in blue and the POI ligand in red.

The clinical translation of TPD has undergone rapid advancement, with numerous degraders progressing through Phase I-III evaluation. Notably, the PROTACs ARV-110 and ARV-471 have undergone significant advancements in clinical trials, with ARV-471 recently entering phase III clinical trials and ARV-110 currently in Phase I trials (Figure 3, Table 1). Arvinas and Pfizer developed ARV-471 (vepdegestrant) to degrade oestrogen receptors for the treatment of oestrogen receptor positive (ER+), HER2-negative breast cancer.^17^ Arvinas has also developed ARV-110 (Bavdegalutamide) for the degradation of androgen receptors in the treatment of metastatic castration-resistant prostate cancer (mCRPC).^18^ Building on these successes, Arvinas has further expanded its portfolio of degraders to include ARV-771 and ARV-766, which target the bromo and extra-terminal domain (BET) family of proteins and androgen receptors respectively (Figure 3).^19^^,^^20^ ARV-766 is currently in Phase I/II clinical trials while ARV-771 has not yet entered clinical trials (Table 1). This pipeline also includes ARV-393, targeting BCL6 for the treatment of relapsed/refractory non-Hodgkin lymphoma (Table 2). Beyond the work of Arvinas & Pfizer, other corporations have developed PROTACs for the degradation of oncogenic proteins, such as RNK05047 by Ranik Therapeutics for the degradation of BRD4 (Figure 3, Table 1). The functionality of RNK05047 depends upon the recruitment of heat shock protein 90, to cause the degradation of the target protein, terming this drug as a chaperone-mediated protein degrader.^20^ Bristol Myers Squibb has also emerged as a frontrunner in the clinical translation of PROTAC technologies through the development of the Phase III candidate BMS-986365 and the Phase I candidate BMS-986458, which degrade the androgen receptor and B-cell lymphoma 6 (BCL6) protein respectively. (Figure 3, Table 1).^21^^,^^22^ The company Foghorn Therapeutics has also made a notable contribution to this field with the PROTAC FHD-609, a Phase I therapeutic capable of degrading the protein BRD9 in the treatment of synovial sarcoma and SMARCB1-deficient tumours (Table 1).^23^ Kymera Therapeutics has introduced a series of degraders aimed at modulating key immune and inflammatory signalling pathways. The PROTACs KT-474 and KT-413 target IRAK4 and are in Phase II and Phase I trials respectively (Table 1), In parallel, the STAT3 degrader KT-333 is currently in Phase I/II evaluation for the treatment of haematologic malignancies (Table 1). Similarly, Nurix Therapeutics has advanced the BTK-targeting degraders NX-2127 and NX-5948 for B-cell malignancies (Table 1).

**Table 1.** Clinical-Stage Targeted Protein Degraders and Their Therapeutic Indications.

Company	Degrader	Target	E3 Ligase/ Binding Target	Disease	Highest Phase	Clinical Trial Number
Arvinas/Pfizer	ARV-110	AR	CRBN	Metastatic castration-resistant prostate cancer	Phase I	NCT05177042
Arvinas/Pfizer	ARV-471	ERα	CRBN	oestrogen receptor (ER)-positive and HER2-negative breast cancer	Phase III	NCT05654623
Ranok Therapeutics	RNK-05047	BRD4	HSP90	advanced solid tumours, including diffuse large B-cell lymphoma (DLBCL)	Phase I/II	NCT05487170
Bristol-Myers Squibb	BMS-986453	BCL6	CRBN	Non-Hodgkin lymphoma (NHL)	Phase I/II	NCT06153251
Foghorn Therapeutics	FHD-609	BRD9	CRBN	Advanced synovial sarcoma or advanced SMARCB1-loss tumours	Phase I (Terminated)	NCT04965753
Bristol-Myers Squibb	BMS-986365	AR	CRBN	Advanced prostate cancer	Phase III	NCT06764485
Arvinas/Pfizer	ARV-766	AR	CRBN	Metastatic castration-resistant prostate cancer (mCRPC)	Phase I/II	NCT05067140
Accutar Biotech	AC682	ERα	CRBN	ER⁺/HER2⁻ advanced or metastatic breast cancer	Phase I	NCT05489679
Dialectic Therapeutics	DT2216	BCL-XL	VHL	BCL-XL-dependent leukaemia and cancer cells	Phase I/II	NCT06620302
Kymera Therapeutics	KT-474	IRAK4	CRBN	Atopic dermatitis (AD) and hidradenitis suppurativa (HS)	Phase II	NCT06058156
Kymera Therapeutics	KT-413	IRAK4	CRBN	B-cell non-Hodgkin lymphomas (NHL), MYD88 mutant	Phase I	NCT05233033
Kymera Therapeutics	KT-333	STAT3	VHL	Large granular lymphocytic leukaemia (LGL-L), peripheral T-cell lymphoma (PTCL), and cutaneous T-cell lymphoma (CTCL)	Phase I	NCT05225584
Nurix Therapeutics	NX-2127	BTK	CRBN	Relapsed/refractory B cell malignancies	Phase I	NCT04830137
Nurix Therapeutics	NX-5948	BTK	CRBN	Elapsed or refractory Waldenstrom’s macroglobulinemia (WM)	Phase I	NCT05131022

AR, Androgen Receptor; ERα, Oestrogen receptor α; BRD4, Bromodomain-containing protein 4; BCL6, B-cell lymphoma 6; Bromodomain-containing protein 9;B-cell Lymphoma-extra-large; interleukin-1 receptor-associated kinase 4; Signal transducer and activator of transcription 3; BTK, Bruton’s tyrosine kinase

**Table 2.** Clinical-Stage Targeted Protein Degraders and Their Therapeutic Indications, Continued.

Company	Degrader	Target	E3 Ligase/ Binding Target	Disease	Highest Phase	Clinical Trial Number
C4 Therapeutics	CFT8634	BRD9	CRBN	Synovial sarcoma and SMARCB1-deficient solid tumours	Phase I/II (Terminated)	NCT05355753
C4 Therapeutics	CFT8919	EGFR L858R	CRBN	Non-Small Cell Lung Cancer (NSCLC)	Phase I	NCT06641609
Cullgen Inc.	CG001419	Pan-TRK	CRBN	Pain treatment	Phase I	NCT06636500
BeiGene	BGB-16673	BTK	CRBN	chronic lymphocytic leukaemia (CLL)/small lymphocytic lymphoma (SLL), Waldenström macroglobulinemia (WM), and other types of non-Hodgkin lymphoma	Phase I/II	NCT05006716
Kintor Pharmacutical	GT20029	AR	N/A	Male androgenetic alopecia (AGA)	Phase II	NCT06692465
Astellas Pharma	ASP-3082	KRAS G12D	VHL	KRAS(G12D)-mutated solid tumours	Phase I	NCT05382559
Arvinas/Pfizer	ARV-393	BCL6	CRBN	Advanced or metastatic solid tumours with KRAS G12D	Phase I	NCT06393738
Accutar Biotechnology	AC-699	ERα	CRBN	Advanced or metastatic ER⁺/HER2⁻ breast cancer	Phase I	NCT05654532
Accutar Biotechnology	AC-676	BTK	CRBN	Relapsed/refractory B-cell malignancies	Phase I	NCT05780034
AbbVie	ABBV-101	BTK	CRBN	B-cell malignancies	Phase I	NCT05753501
Prelude Therapeutics	PRT3789	SMARCA-2	VHL	Advanced or Metastatic Solid Tumours w/ SMARCA4 Mutation	Phase II	NCT06682806
Jiangsu Henrui Pharmaceuticals	HRS-1358	AR	N/A	Metastatic or Local Advanced Breast Cancer	Phase I/II	NCT06679036
Jiangsu Henrui Pharmaceuticals	HRS-5041	AR	N/A	Advanced or metastatic castration-resistant prostate cancer	Phase I	NCT06568094

AR, Androgen Receptor; ERα,Oestrogen receptor α; BRD4, Bromodomain-containing protein 4; BCL6, B-cell lymphoma 6; Bromodomain-containing protein 9;BCL-XL, B-cell Lymphoma-extra-large; BTK, Bruton’s tyrosine kinase; EGFR, Epidermal growth factor receptor; Pan-TRK, Pan-tropomyosin receptor kinase; SMARCA-2, SWI/SNF-related, matrix-associated, actin-dependent regulator of chromatin, subfamily A, member 2, KRAS G12D, Kirsten rat sarcoma virus.

Several emerging compounds further illustrate the growing clinical translation of this modality. The company C4 Therapeutics has initiated first in-human studies of CFT8634, a BRD9 degrader for synovial sarcoma, and CFT8919, a mutant EGFR (L858R) degrader for non-small-cell lung cancer (Table 2). The pan-TRK degrader CG001419 by Cullgen is currently in clinical trials for pain and Neurotrophic tyrosine receptor kinase (NTRK)-driven tumours (Table 2). BeiGene’s BGB-16673, a BTK degrader, is progressing through Phase 1/2 studies in relapsed/refractory B-cell malignancies (Table 2). Early results for this compound demonstrate durable BTK knockdown. Other recent entries into clinical translation include AC682 and AC699, oestrogen receptor alpha degraders by Accutar Biotechnology (Tables 1and 2). Accutar along with AbbVie have also made contributions to the progression of BTK degraders through the compounds AC676 and ABBV-101 respectively (Table 2). These listed programs mark a pivotal expansion of PROTAC degraders into the clinical landscape and serve to exemplify the momentum of furthering PROTAC efficacy and diversification. The growing entry of PROTACs into clinical evaluation by multiple pharmaceutical companies alongside the increasing advancements in target diversification underscore their evolution beyond proof-of-concept validation and highlights increasing interest in TPD as a promising therapy for cancer, while the extent to which this therapeutic confers improved safety or efficacy remains an active area of investigation.^24–27^

**Figure 4.**
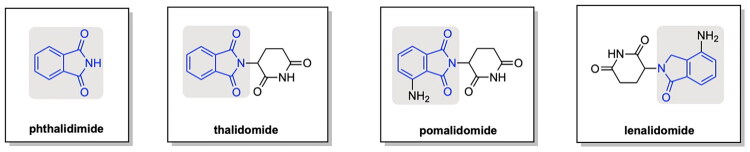
Structural Comparisons of Phthalimide and its IMiD derivatives. Chemical structures of phthalimide-based immunomodulatory drugs (IMiDs). Phthalimide serves as the core scaffold and is present in derivatives such as thalidomide, pomalidomide and lenalidomide. Structural modifications distinguishing each compound include the presence of a glutarimide moiety attached to the phthalimide core, along with the presence of an amino group on the aromatic ring of the derivative. These structural differences influence the biological activity of each compound and binding affinity to CRBN (cereblon), an E3 ligase commonly leveraged in PROTAC designs.

At the molecular level, much of the progress in PROTAC design and applications have been driven by degraders exploiting the cereblon (CRBN) E3 ligase, which has emerged as the most versatile and widely adopted scaffold in degrader design. The development of PROTACs targeting the CRBN E3 ligase has become a cornerstone to advancements in the applications of this therapeutic for targeted protein degradation, as seen in the discussion of PROTAC developments by Arvinas. The work of Winter et al. introduced a PROTAC targeting BRD4, a key protein in epigenetic regulation and tumour cell proliferation, and their approach utilised the CRBN targeting ligand phthalimide.^28^ This notable milestone was facilitated by the discovery of phthalimide immunomodulatory imide drugs (IMiDs) as a ligand to the CRBN E3 ligase, serving to enable a novel utility of thalidomide, pomalidomide and lenalidomide for the development of PROTAC variants (Figure 4).^10^

**Figure 5.**
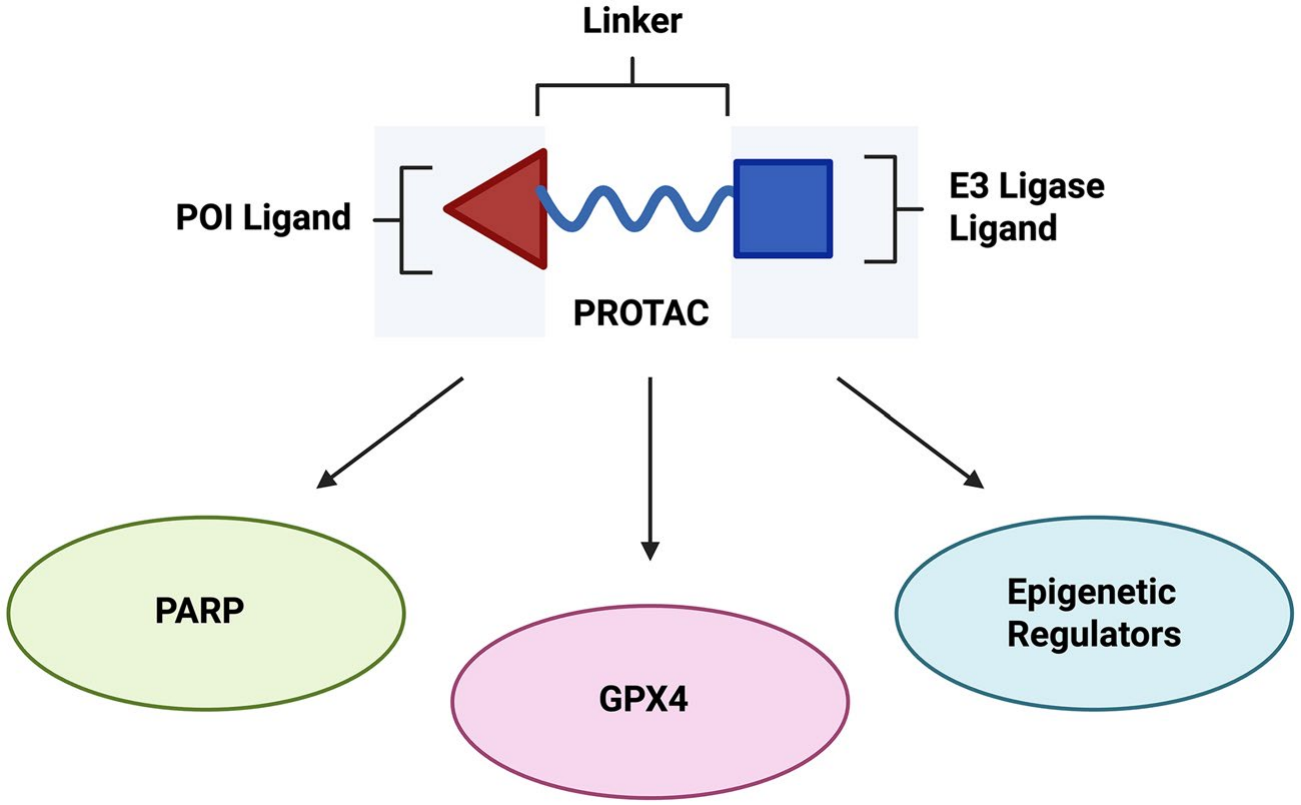
Protein targets for degradation by PROTACS: PARP, GPX4, and Epigenetic Regulators proteins. Overview of the structure of PROTACs, consisting of a ligand for the POI (protein of interest), and an E3 ligase ligand connected by a linker. The three categories of PROTACs emphasised in this paper, namely PARP, GPX4 and epigenetic regulators are depicted. (created via BioRender on 29 April 2025).

Advancements in PROTAC translation to the clinic have also progressed for the targeted degradation of zinc finger proteins, particularly key zinc finger transcription factors such as IKZF1 and IKZF3.^29^ Zinc finger proteins are a class of transcription factors having multifaceted roles in metabolism, development and autophagy, along with roles in cancer proliferation. Recent PROTAC developments have aimed to degrade this class of proteins as a means of treating multiple myeloma and non-Hodgkin’s lymphoma, with the previously mentioned phthalimide derivatives constituting a significant portion of current zinc finger protein degraders. The development of CFT7455 (Cemsidomide) by C4 Therapeutics has expanded upon the current progress in IKZF1/3 degradation through introducing a high affinity binding of the CRBN E3 ligase superior to that of formerly utilised phthalimide derivatives.^30^ The utility and immense potential of PROTACs for targeted protein degradation is exemplified by (i) the catalytic mode of action,^31^^,^^32^ (ii) versatility in ligand and linker choice^33^^,^^34^ and (iii) their potential to target ‘undruggable’ proteins.^35^^,^^36^ Numerous studies serve to discuss the progression of PROTAC development and novel advancements since their initial discovery.^6^^,^^30^^,^^37–40^ Among these present reviews, the advancements of PROTACs for the degradation of cancer-associated proteins is also discussed.^41–43^ In this paper, we seek to highlight the recent developments of PROTACs for the degradation of three specific protein categories, namely PARP, GPX4 and epigenetic regulators. Specifically, we intend to provide an updated review of novel PROTACs for GPX4 due to the rapid emergence of this subset of PROTAC development in recent months. In this paper, we have included work from the initial discovery of the PROTAC concept in 2001 to PROTAC variants that have been published in 2025. Herein, we report the synthesis of selected PROTAC variants, specifically those molecules identified as the most potent in the corresponding publication as per the provided half maximal inhibitory concentration (IC_50_), half maximal degradation concentration (DC_50_) and maximum percent of protein degradation achieved (D_max)_ values. We apologise in advance for the possible neglect of any relevant work including the synthesis and advancements of PROTACs. In the following sections, we will discuss the synthesis and biological testing data of PROTACs for the degradation of PARP, GPX4 and epigenetic regulator proteins (Figure 5).

The three classes of targets highlighted in this review, namely PARP, epigenetic regulators, and GPX4, were selected to represent distinct, yet interconnected stress-response networks that converge on ferroptosis. While each protein has unique canonical roles in cellular homeostasis: PARP in DNA repair, epigenetic regulators in transcriptional plasticity, and GPX4 in redox balance, their inhibition triggers signalling cascades that ultimately hinder ferroptosis resistance mechanisms and promote cell death. Inhibition of these protein classes induces ferroptosis through diverse, yet converging in mechanisms that impact redox homeostasis lipid metabolism and iron handling. In recent studies, PARP inhibition has been identified to induce ferroptosis through multifaceted intersecting pathways. The treatment with PARP inhibitors such as olaparib activates the tumour suppressor transcription factor p53, resulting in the transcriptional repression of solute carrier family 7 member 11 (SLC7A11), the cystine/glutamate antiporter that maintains intracellular glutathione (GSH) levels necessary for GPX4 function.^44^ The inhibition of PARP has also been shown to activate the cGAS-STING-ATF3 axis, which serves to repress SLC7A11 and weakens GPX4-dependent detoxification.^44^ Beyond the transcriptional effects of PARP inhibition, this blockade has been shown to directly elevate levels of lipid peroxidation. The small-molecule inhibitor Niraparib has been showed to upregulate the fatty acid transporter cluster of differentiation 36 (CD36), thereby increasing fatty acid uptake, lipid accumulation and the formation of lipid peroxides that drive ferroptotic death.^45^

Epigenetic regulators likewise influence ferroptosis through the control of genes encoding proteins involved in maintaining redox and iron balance. The tumour suppressor BRCA1-associated protein 1 (BAP1) promotes ferroptosis through the deubiquitinating of histone 2 A to repress SLC7A11 transcription, while the protein USB7 deubiquitinates histone H2B and stabilises the p53 tumour suppressor, serving to reinforce the SLC7A11 suppression and the associated increase in ferroptosis.^46^ In particular, several epigenetic regulators implicated in ferroptosis, such as Enhancer of zeste homolog 2 (EZH2), histone deacetylates (HDAC) and the BET family of proteins, represent major targets in the emerging field of PROTAC based therapeutic development, as discussed in this review. The inhibition of the histone methyltransferase EZH2 has been identified to disrupt the EZH2-HIF-1α co-repressive complex, preventing the repression of Acetyl-CoA Synthetase Long-Chain Family Member 4 (ACSL4), a key promoter of lipid peroxidation.^47^ This inhibition also serves to reduces histone H3 trimethylation to increase the transferrin receptor 2 (TFR2)-mediated iron uptake.^48^ Inhibition of the HDAC family of proteins has been identified to reprogram iron metabolism^49^ through enhancing ferritinophagy and iron release. The resulting upregulation of nuclear receptor coactivator 4 (NCOA4), haem oxygenase (HMOX1/2) and transient receptor potential mucolipins (TRPML) and increase in intracellular iron flux combined with the reduced SLC7A11-mediated cystine uptake promotes reactive oxygen species generation and lipid peroxidation.^50^^,^^51^ Additionally, HDAC inhibition enhances p53 acetylation and downregulates signal transducer and activator of transcription 3(STAT3)-dependent GPX4 expression while suppressing iron sequestration, collectively heightening ferroptosis susceptibility.^52^ The inhibition of the bromodomain protein BRD4, a member of the BET protein family, further contributes to ferroptosis through the coordinated regulation of antioxidant and iron pathways.^53^^,^^54^ BET inhibitors such as (+)-JQ1 downregulate lipid peroxide defense enzyme ferroptosis suppressor protein 1 (FSP1) through the disruption of BRD4 binding at the FSP1 promoter.^55^ They also induce ferritinophagy, releasing free Fe^+2^ that fuels the generation of reactive oxygen species (ROS) and suppresses the antioxidant genes encoding GPX4, SLC7A11 and SLC3A2, thereby weakening redox defences. The nuanced regulatory effects of PARP and epigenetic regulators converge to activate key triggers of ferroptosis through their inhibition. As emerging research continues to elucidate the multidimensional downstream effects of these proteins, a clear precedent has been established for exploring synergistic therapeutic strategies, particularly those leveraging PROTAC-based degradation to target these regulators.

### Methodology for bibliographic search

We used the following databases to survey the literature reporting PROTAC degraders for pathogenic proteins, along with the key biological processes underlying the malignancy of such target proteins and the functionality of designed targeted degradation therapies: 

- SciFinder website at: https://scifinder.cas.org/
- PubMed website at: https://www.ncbi.nlm.nih.gov/pubmed/

The following criteria were utilised to identify articles for the scope of this review: 

- Articles must report on the development of PROTACs for targeting GPX4, PARP, or epigenetic regulator proteins
- Articles must provide relevant biological background for protein of interests in PROTAC-based therapies
- Articles must provide the synthetic scheme for PROTAC design along with biological assay data
- Articles must detail the incentive to utilise the PROTAC approach for protein targeting

## PROTACs for cancer treatment

### PROTACs for PARP degradation

#### Biology

PROTACs hold great potential in the targeting of disease associated proteins for pathologies beyond cancer, such as for neurodegenerative diseases, autoimmune and inflammatory diseases, cardiovascular diseases as well as inflammatory conditions.^56–59^ The heterobifunctional design of these therapeutics can resolve limitations associated with traditional small molecule inhibitors pertaining to the necessity for high affinity interactions between inhibitors and target proteins. The employment of a PROTAC-based approach to protein degradation allows for improvements towards selectivity, owing to the ternary complex formation, along with a reduced likelihood for acquiring drug resistance.^60^ PARP or poly (ADP-ribose) polymerase is a family of nuclear proteins with key roles in the repair of DNA damage, particularly for the recruitment of proteins in base excision single strand break repair and double stranded break repair pathways.^61–63^ This protein works to synthesise a polymeric adenosine diphosphate ribose (PAR) chain through the transfer of an ADP-ribosyl moiety from NAD+ to acceptor proteins. Through the addition of the PAR chain, components of base excision repair for trimming of the abasic site, along with repair tools such as DNA Polymerase β and DNA Ligase III are recruited.^64^ This family of proteins consists of around 18 members that are predominantly involved in DNA repair processes but have additional functions in the proliferation and death of cells.^65^^,^^66^ PARP plays a critical role in DNA repair, and its inhibition is particularly effective in BRCA-mutated cancers, where homologous recombination is compromised. The abnormal functioning of PARP that contributes to cancer is associated with mutations of the BRCA1 (breast-cancer-susceptibility-gene 1) and BRCA2, tumour suppressor genes that are involved in the repair of double stranded DNA breaks using homologous recombination.^67^^,^^68^ In the presence of poor BRCA1 and BRCA2 functionality, mammalian cells rely on a nonconservative mechanism of DNA repair such as NHEJ (non-homologous end joining), serving to leverage proteins such as PARP for continued cell proliferation.^69–71^ Treatment of cancer through PARP inhibition has thus become a promising therapeutic approach, as seen through recent advancements, both in the pre-clinical and in the clinical stages.^72^^,^^73^ Numerous successes have been seen in the synthesis of PARP inhibitors, such as Olaparib, Niraparib, and Rucaparib. However, the requirements of high affinity interactions that govern the efficacy of small molecule drugs and their interactions with target proteins are inherently a limitation to the targeting of PARP.^74^ Such limitations can be addressed with the utilisation of PROTACs, a promising therapeutic modality, for PARP inhibition and degradation.^75^

#### Synthesis

**Figure 6.**
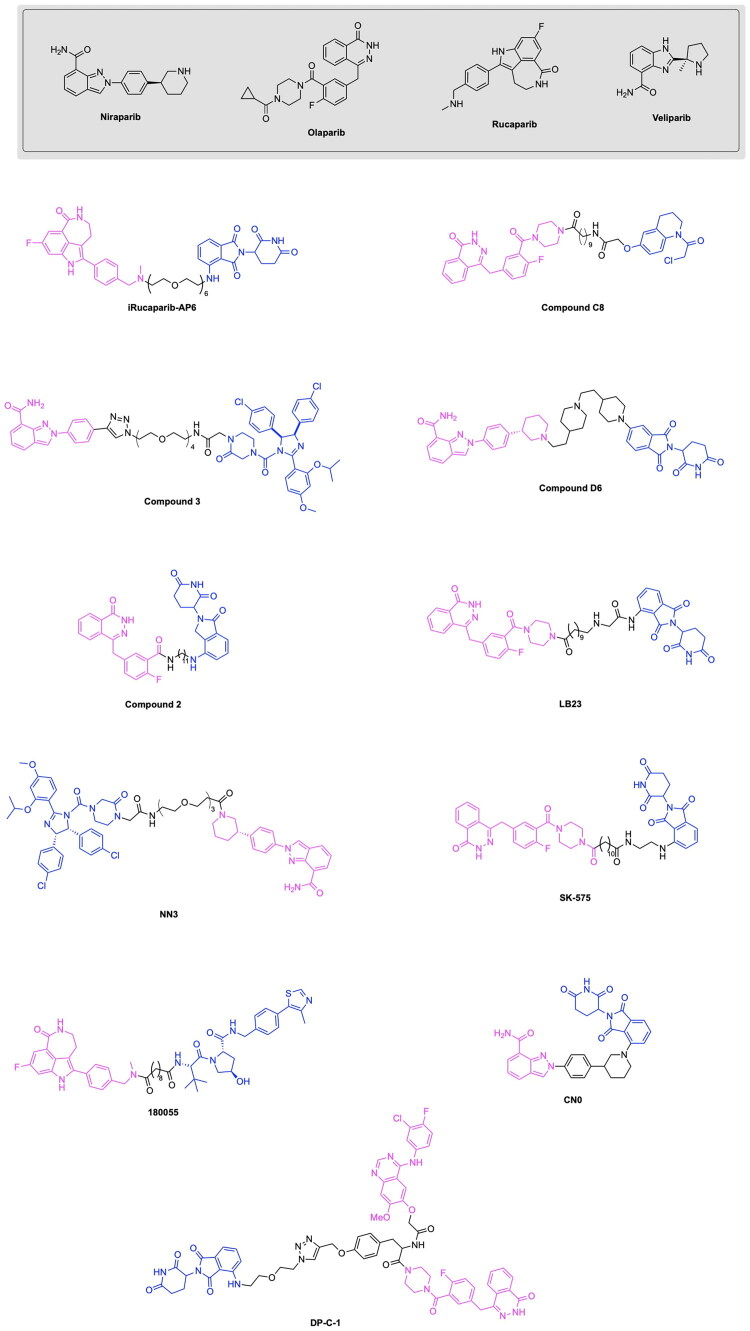
Overview of PARP degraders covered in the subsequent section on notable PARP targeting PROTACs..^76–85^

**Scheme 1.**
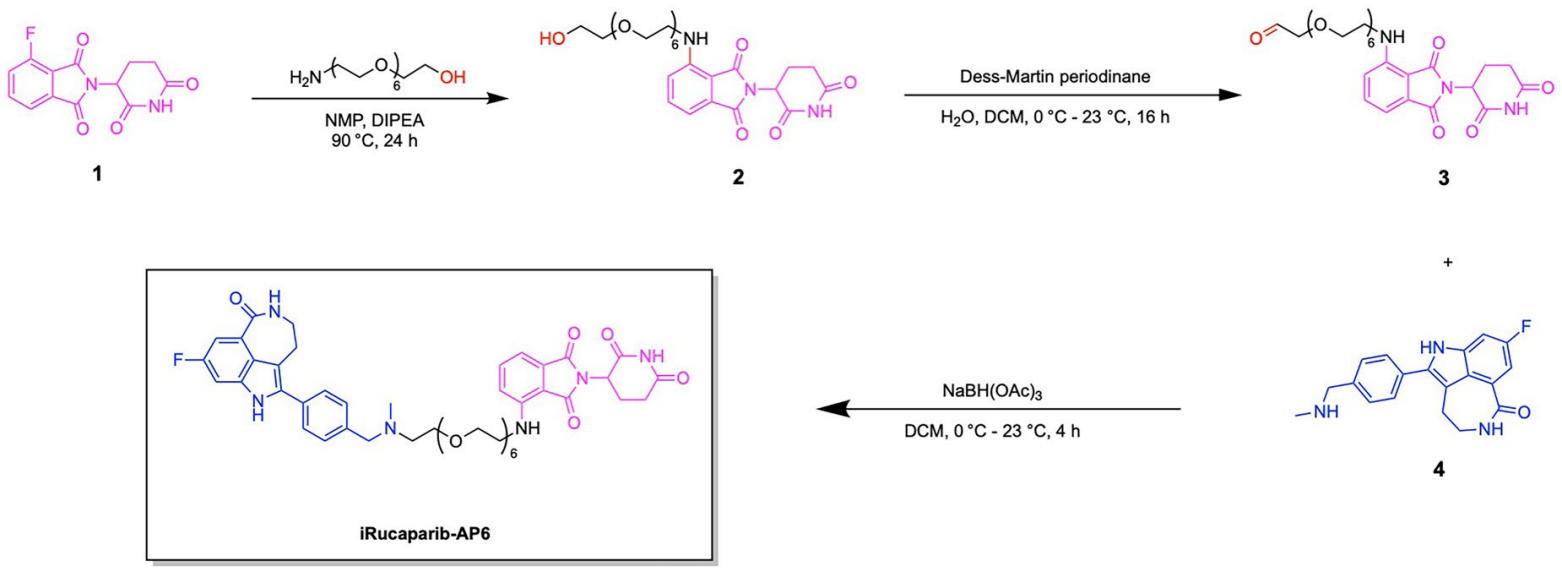
Synthesis route of iRucaparib-AP6 adapted from reference.^76^

The first PARP degrading PROTAC was reported by the Rao group through the synthesis of Compound 3, thereby pioneering the emergence of heterobifunctional designs for PARP degraders.^79^ Compound 3 achieved an IC_50_ of 6.12 μM following a 48-h treatment in the MDA-MB-231 cell line, exhibiting promise in the field of PARP targeting PROTACs (Figure 6, Table 3).^78^ The PARP inhibitor Niraparib was functionalised at the piperidine ring for attachment to a linker and the E3 ligase ligand nutlin-3. A total of five analogs were synthesised, incorporating variations in the POI ligand (Niraparib or Olaparib) and the E3 ligase ligand, such as ligands for CRBN, VHL, and MDM2. The cleavage of PARP1 due to PROTAC activity was confirmed through a pre-treatment competition assay, in which treatment with Niraparib and Nutlin-3 yielded the complete and partial blockage of Compound 3-induced PARP1 cleavage respectively.^78^ The effect of PARP1 cleavage in inducing apoptosis was studied by treating MDA-MB-231 cells with Compound 3, followed by annexin V-FITC/PI staining. The externalisation of phosphatidylserine and the presence of cleaved caspase-3 indicated an apoptotic response due to Compound 3 treatment. Current PARP1 inhibitors induce tumour cell death through both PARP1 catalytic inhibition and PARP1 trapping, with inhibition being the ideal strategy of preventing the role of PARP in cancer cell proliferation. Wang et al. synthesised the novel PROTAC iRucaparib-AP6 along with other small molecule PARP degraders for exclusively inducing PARP depletion and preventing the associated pathological conditions of PARP overexpression in cancer (Figure 6, Table 3).^76^ A series of small molecule PARP degrading PROTACs utilising E3 ligase ligand, linker and warhead variations were created to identify iRucaparib-AP5 and iRucaparib-AP6 as the most promising degraders of PARP1. This optimal PARP1 degrading PROTAC is based on the FDA-approved PARP1 inhibitor Rucaparib linked to a pomalidomide, a ligand to the E3 ligase CRBN, through a five and six-unit PEG linker respectively. In their initial screening of optimal PROTAC structures, they identified the profound importance of linker length on degradation properties and the optimal derivatization site on Rucaparib to be a tether attached at the amino group distal to the indolyl lactam moiety in this inhibitor.^76^ They also revealed the superiority of pomalidomide in comparison to the VHL ligand for inducing PARP1 degradation, along with the inferior degradation associated with replacing the Rucaparib warhead with other PARP1 inhibitors in the clinic such as Olaparib and Veliparib. The successful PARP1 PROTACs iRucaparib-AP5 and iRucaparib-AP6 achieved DC_50_ values of 36 nM and 82 nM respectively in primary rat neonatal cardiomyocytes *in vitro*.^76^ The synthesis of the potent PROTAC iRucaparib-AP6 is depicted in Scheme 1 and was achieved with the following steps: A nucleophilic aromatic substitution was performed on compound **1** (4-fluoro thalidomide) using amino-PEG6-OH, Hünig’s Base (DIPEA) and the solvent N-methyl-2-pyrrolidone (NMP) to afford compound **2**. This product was then oxidised using Dess-Martin periodinane in water and dichloromethane (DCM) to yield compound **3**. This pomalidomide derivative was reacted with a PARP inhibitor compound **4** (Rucaparib) in a reductive amination condition using sodium triacetoxyborohydride to afford the final PROTAC product (iRucaparib-AP6).

**Table 3.** Summary of Reported PARP-Targeting PROTACs and Their In Vitro Potency Profiles.

PROTAC	POI	E3 Ligase	Warhead	Linker Motif	Cell Line	In Vitro Potency/Affinity	References
iRucaparib-AP6	PARP1	CRBN	Rucaparib	PEG	Primary rat neonatal cardiomyocytes	DC_50_: 82 nM D_max_ : 92%	71
Compound C8	PARP 2	DCAF16	Olaparib	Alkyl	MDA-MB-231	DC_50_: 2 µM D_max_ = 92%	72
Compound 3	PARP1	MDM2	Niraparib	PEG	MDA-MB-231	IC_50_: 6.12 µM	73
Compound D6	PARP1	CRBN	Niraparib	Piperadine	MDA-MB-231	DC_50_: 25.23 nM IC_50_: 1.04 µM	74
Compound 2	PARP1	CRBN	Olaparib	Alkyl	SW620	DC_50_: 5.4 µM	80
LB23	PARP1	CRBN	Olaparib	Alkyl	MDA-MB-231	DC_50_: 53 nM	75
NN3	PARP1	MDM2	Niraparib	PEG	MDA-MB-231	IC_50_: 13.97 µM	76
SK-575	PARP1	CRBN	Olaparib	Alkyl	MDA-MB-436, Capan-1	IC_50_: 2.30 µM IC_50_ in MDA-MB-436: 0.019 µM IC_50_ in Capan-1: 0.056 µM	77
180055	PARP1	VHL	Rucaparib	Alkyl	T470, MDA-MB-231	DC_50_ in T470: 180 nM DC_50_ MDA-MB-231: 240 nM	78
CN0	PARP1	CRBN	Niraparib	None	MDA-MB-231, 4T1	CI for CN0 (10 M) and Daunorubicin (0.25 µM): MDA-MB-231: 0.21 4T1: 0.44	79
DP-C-1	PARP, EGFR	CRBN	Olaparib, Gefitinib	PEG	N/A	K_d_ for PARP: 7.81 µM K_d_ for EGFR: 2.74 µM	82

DC_50_, half-maximal degradation concentration; D_max_, maximal fraction of a target protein that is degraded; IC_50_, half-maximal inhibitory concentration; EC_50_, half-maximal effective concentration; K_d_, dissociation constant

Further advancements in the field of PARP degrading PROTACs have been accomplished by Zhang et al. in the synthesis of Compound 2 (Figure 6).^85^ The authors selected the PARP inhibitor Olaparib and covalently linked this POI ligand to lenalidomide through linkers of varying alkyl chain lengths, namely five carbon, eight carbon and nine carbon linkers. Structure-activity relationship data was used to identify cyclopropyl(piperazin-1-yl)methanone as a solvent exposed moiety suitable for functionalization in the PROTAC design. A DC_50_ value of 5.4 μM in SW260 cells was obtained following a 24-h incubation (Table 3).^85^ Upon investigating the role of Compound 2 in apoptosis and cell cycle arrest, it was determined that the degrader caused apoptosis in a dose dependent manner, with a 10 μM concentration of Compound 2 resulting in a 60% apoptosis ratio.^85^ Furthermore, Compound 2 arrested the cell cycle in the G1 phase, whereas existing PARP inhibitors arrested the cell cycle in the G2 phase. The metabolic stability of Compound **2** was investigated *in vitro* using human liver microsomes (HLMs). A high instability of Compound **2** was revealed, due to the metabolisation of 90% of the PROTAC following a 30-min time period.^85^ Cao et al. expanded on the synthesis of Olaparib-based PARP degraders through the development of the PROTAC SK-575, achieving a DC_50_ value of 1.26 nM and 0.509 nM in the tumour cell lines MDA-MB-436 and SW620 cells respectively.^82^ The authors leveraged the cyclopropyl moiety of Olaparib for the attachment of diverse linkers and a thalidomide/lenalidomide CRBN-binding ligand. A strategic approach was taken in the testing of linker variants through the utilisation of amino acid linkers, such as 4-aminobutyric acid and 6-aminocaproic acid. Due to the relatively low potency exhibited by the synthesised amino acid linkers, a series of alkyl dicarboxylic acid linkers were tested. This alteration in linker design enabled the discovery of the potent PARP degrader SK-575, containing a 12-carbon dodecarboxylic acid linker along with a thalidomide E3 ligase ligand (Figure 6, Table 3).^82^ Further optimisation efforts towards the choice of E3 ligase ligand were conducted, revealing the lower potency associated with PROTAC analogs containing a VHL ligand. The authors further examined the ability of SK-575 and the inhibitor Olaparib to suppress the cell growth inhibition of HR-deficient cell lines and HR-proficient cell lines. It was determined that both SK-575 and Olaparib demonstrate inhibition of both types of cell lines under investigation, with SK-575 exhibiting higher potency due to the advantages of protein degradation over inhibition.^82^ Lin et al. further contributed to the growing field of PARP degraders through the synthesis of CN0, a novel PROTAC achieving the degradation of PARP1 in the MDA-MB-231 cell line (Figure 6, Table 3).^84^ The degrader is comprised of a CRBN-recruiting pomalidomide directly tethered to the PARP1 inhibitor Niraparib in the absence of a linker, thereby representing the effectiveness of a minimalistic design approach to heterobifunctional degraders. The compound demonstrated selectivity for PARP1 degradation, while resulting in little to no degradation of PARP2 and PARP3 proteins. Competitive inhibition studies utilising the proteasome inhibitor MG132 and the E1 activating enzyme inhibitor MLN4924 revealed the presence of a proteolytic knockdown of PARP1. The PROTAC CN0 exhibits promise in facilitating cytotoxicity through a combined treatment with the DNA-damaging agent daunorubicin, which serves to activate downstream STING signalling.^84^ This effect is evidenced by the presence of elevated phosphorylation of key proteins in the innate immune response system, namely TANK-binding kinase 1 (TBK1), Interferon regulatory factor 3 (IRF3) and Stimulator of interferon genes (STING). An increased expression of inflammatory chemokines such as CXCL3 and CXCL10, along with the tumour suppression of an in vivo 4T1 allograft mouse model further indicates the efficacy of this degrader.

**Scheme 2.**
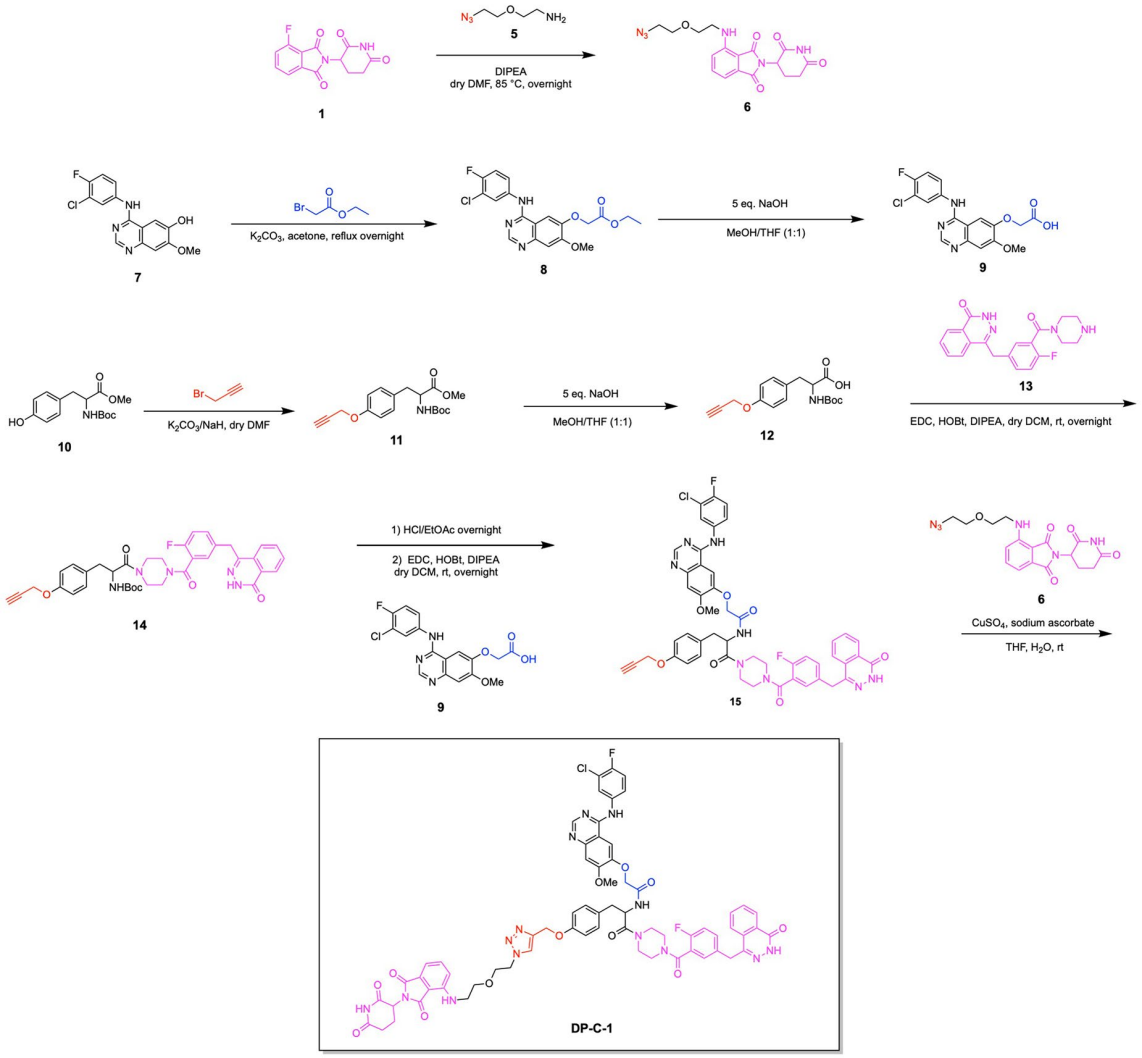
Synthesis Pathway for DP-C-1 adapted from reference.^87^

The PROTAC model has also been successfully employed to facilitate the degradation of additional cancer-associated proteins along with PARP using a singular heterobifunctional molecule. Due to the upregulation of multiple proteins for chemoresistance, the sole degradation of exclusively one protein as a means of cancer treatment serves to be associated with limitations towards reducing cancer proliferation. In relation to the role of PARP in cancer, the downregulation of base-excision repair agents serves to facilitate the sensitivity of cancer cells to PARP inhibition. The degradation of epidermal growth factor receptor EGFR serves to have this favourable function towards increasing the impact of PARP inhibition in facilitating tumour cell death.^86^ Zheng et al. designed a novel dual PROTAC utilising a star-type linker to converge the EGFR inhibitor Gefitinib, the PARP inhibitor Olaparib and an E3 ligase ligand into a single trifunctional protein degradation tool (Figure 6).^87^ A convergent synthetic strategy was employed, involving the alkylation of the hydroxyl group of a serine or tyrosine-based linker with an alkyne moiety followed by amide formation reactions with EGFR and PARP inhibitors. The E3 ligase ligand of interest was then attached to the added alkyne group via a click chemistry reaction to form a triazole based linkage. The synthesis of four CRBN ligand-based dual-PROTACs, four VHL ligand-based dual-PROTACs and four mono-PROTACs were successfully achieved, with variations of serine or tyrosine linkers within the listed categories of PROTACs. All four CRBN based dual-PROTACS exhibited dual degradation effects, with an IC_50_ value of 19.92 μM obtained for the potent variant DP-V-4, having a VHL ligand and serine linker-based.^87^ Binding affinities of the trifunctional PROTACs to the two POIs was noted from the obtained Kd values for the binding of DP-V-4 to EGFR and PARP (5.47 μM and 12.80 μM respectively) and the binding of DP-C-1 (CRBN ligand with a tyrosine linker) to EGFR and PARP (2.74 μM and 7.89 μM respectively) (Figure 6, Table 3).^87^ The synthesis of DP-C-1 is depicted in Scheme 2.^87^ Compound **1** (4-fluoro thalidomide) was reacted with azido-PEG1-amine (compound **5**) in a nucleophilic aromatic substitution reaction to afford the azide-terminated CRBN ligand (compound **6**). The following portion of the synthesis details the covalent linkage of the EGFR inhibitor Gefitinib and the PARP inhibitor Olaparib. Compound **7** (Gefitinib) was alkylated using ethyl bromoacetate via a Williamson ether synthesis reaction to yield the ester compound **8,** which subsequently underwent ester hydrolysis using sodium hydroxide (NaOH) to afford the acid compound **9.** On the other hand, the phenol of compound **10** was alkylated using propargyl bromide via another Williamson ether synthesis reaction to afford the ether compound **11**, followed by its ester hydrolysis to afford the acid compound **12**. Subsequently, this acid was coupled with the secondary amine compound **13** (Olaparib derivative) using 1-Ethyl-3–(3-dimethylaminopropyl)carbodiimide (EDC) and 1-hydroxybenzotriazole (HOBt) to yield the amide-containing compound **14**. The latter underwent an amine deprotection, followed by another EDC coupling reaction with the previously synthesised acid (compound **9)** to afford the second amide bond formation (compound **15)**, which was then reacted with the azide-terminated CRBN ligand (compound **6)** via a copper-catalysed azide alkyne cycloaddition to result in the dual-PROTAC variant DP-C-1.

**Scheme 3.**
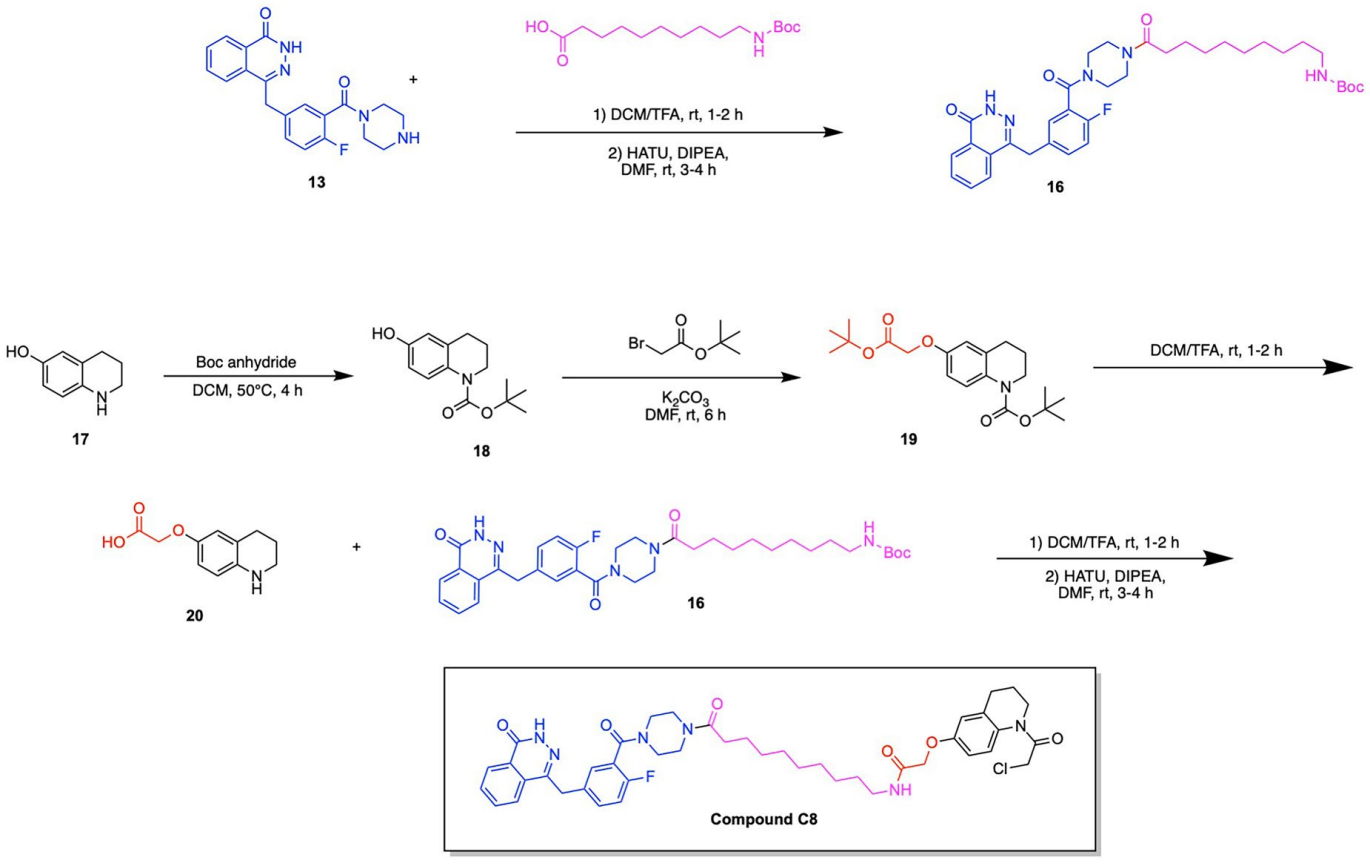
Synthesis route of Compound C8 adapted from reference.^77^

Although the majority of developed PROTACs targeting the PARP protein have focused on the degradation of PARP1, the work of Pu et al. has made a significant contribution to the scope and specificity of PARP proteins targeted for degradation through the development of the PARP2 targeting PROTAC C8.^77^ The incentive to target PARP2 exclusively lies in the differing mechanisms by which the two PARP protein types contribute to tumour development.^88–90^ Recent studies have demonstrated that the deficiency of both PARP1 and PARP2 in T-cells is associated with tumour growth, whereas the deficiency of solely PARP2 exhibits the desirable effect of limiting tumour progression.^91^ This recent finding regarding the role of PARP2 targeting in preventing cancer proliferation has incentivised the investigation of methods by which solely PARP2 can be degraded, while employing current PARP inhibitors targeting PARP1/PARP2 in the molecular design. An innovative approach was taken in the utilisation of a ligand for the nuclear-localized E3 ligase DCAF16. The small molecule inhibitor of DCAF16, known as KB02, is known to covalently bind to the cysteine residue of DCAF16 and subsequently cause the inhibition of this E3 ligase.^92^ The authors intelligently leveraged the cysteine binding properties of KB02 to cause the prevention of PARP1 degradation and the enablement of PARP2 degradation, due to the inherent structural difference present between PARP1 and PARP2 proteins. It is known that PARP1 contains a cysteine residue near the binding pocket, whereas the POI PARP2 does not. It was hypothesised that in the presence of PARP1 binding by a PARP inhibitor, KB02 would form a cysteine bond with PARP1, thereby preventing the necessary interaction with the E3 ligase DCAF16 and the degradation of PARP1.^77^ Based on this knowledge, a series of analogs were synthesised, containing Olaparib covalently bound to a variety of linkers, as well as to KB02. Among the developed analogs, the PROTAC compound C8 emerged as the most potent compound, having a DC_50_ of 2 μM in the MDA-MB-231 cell line (Table 3, Figure 6).^77^ The series of linker optimizations conducted through the synthesis of several PROTAC analogs revealed that an alkyl linker yielded the most optimal antiproliferative activity in four cancer cell lines, namely MDA-MB-436, MDA-MB-468, MDA-MB-231, and Capan-1. The iterative design of analogs revealed the necessity of the presence of carbonyl on the piperazine ring of Olaparib, as well as the absence of an amide bond in the middle of the linker chain.^77^ Compound C8, consisting of an 8-carbon linker chain, was thus determined as the most potent and carried forward for biological assays. The antiproliferative activity of C8 *in vitro* was confirmed through treating a series of cancer cell lines with the developed PROTAC. It was observed that C8 inhibits the growth of MDA-MB-436, Capan-1, MDA-MB-468 and MDA-MB-231 cancer cell lines.^77^ An investigation of the role of C8 in cell cycle arrest and apoptosis revealed that the cell cycle progression was arrested at the G2/M phase in both the MDA-MB-436 and MDA-MB-231 cell lines. Additionally, C8 promoted apoptosis in the MDA-MB-231 cells by around 20% at a 1.25 μM concentration of the PROTAC.^77^ The synthesis of the Compound C8 is depicted in Scheme 3 and was accomplished with the following steps. An amide coupling between an alkyl-based linker and compound **13** preceded by the deprotection of compound **13** was performed to afford compound **16**. The secondary amine of compound **17** was protected using Boc anhydride to afford compound **18**, which subsequently underwent a Williamson ether reaction with tert-butyl bromoacetate to yield compound **19**. Compound **19** was deprotected to yield compound **20**, which was reacted with compound **16** to afford the PROTAC compound C8.

**Scheme 4.**
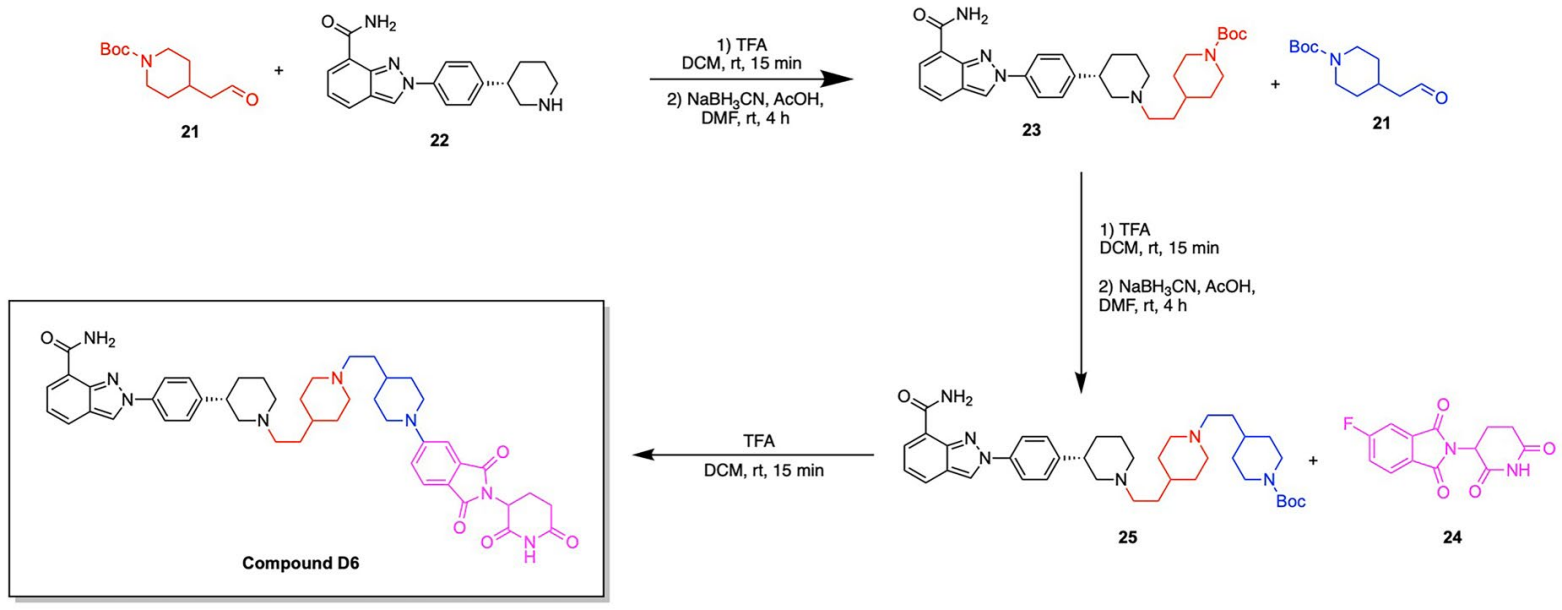
Synthesis route of Compound D6 adapted from reference.^79^

The work of Pu et al. has further contributed to the growing field of PARP PROTACs through the development of the degrader LB23 (Figure 6, Table 3).^80^ This study was incentivised by the potential of linker and ligand optimisation to improve potency and biological properties, thereby adding to the increasing progress being made in PARP degraders. A series of analogs were synthesised through the covalent linkage of the PARP inhibitor Olaparib to a series of linkers and thalidomide/pomalidomide CRBN ligands. Due to the previously established importance of Olaparib-linker covalent conjugation in formerly published PARP degraders, the authors designed analogs with the absence and the presence of a carbonyl group on the piperazine ring of Olaparib. The PROTAC LB23 was determined to have the most favourable degradation properties, with an IC_50_ value of 31 nM in the MDA-MB-436 cell line and 77 nM in Capan-1 cells.^80^ The role of LB23 as a PROTAC utilising the ubiquitin proteasome system was confirmed through a series of competition experiments, in which a blockage of PARP degradation due to LB23 was observed in the presence of Olaparib, pomalidomide and the proteasome inhibitor MG132. A comparison with the previously discovered PROTAC SK-575 by Cao et al. revealed that LB23 exhibits 60-fold selectivity for tumour cells over the human normal liver cell line L-O2, whereas SK-575 demonstrated only 5-fold selectivity.^80^ The achievement of reduced toxicity in PARP degrader design was attributed to the incorporation of a 2-amino acetamide linker in LB23, whereas an amide linker was present in SK-575. It was further determined that LB23 and Olaparib both arrested MDA-MB-231 cancer cells in the G2/M phase of the cell cycle.^80^The optimisation of linker design has not only been a key source of iterative PROTAC design and improvement, but also a fundamental driving force of developing PARP degraders with higher potency and selectivity. Wu et al. adopted an innovative approach to linker design by utilising nitrogen-containing heterocyclic linkers in the development of the PROTAC compound D6 (Figure 6, Table 3).^79^ A computational approach was taken towards the development of PROTAC analogs through the utilisation of ternary complex modelling. The initial hypothesis regarding the beneficial role of nitrogen heterocycles in PROTAC linkers was developed through the observed superior degradation capabilities of PROTAC analogs containing two piperidinyl groups as opposed to one piperidinyl group. The effect of nitrogen heterocycles in improving the potency of a synthesised analog prompted the usage of ternary complex formation studies using MOE. The obtained docking scores revealed that the incorporation of nitrogen heterocycles in linker design lowered the energy of the ternary complex, a result attributed to the involvement of piperidine rings in hydrogen bonding, carbon-hydrogen bonds and alkyl interactions at the protein-protein interface.^79^ The series of analogs were constructed through the covalent linkage of Olaparib/Niraparib to a thalidomide E3 ligase ligand using a diverse range of linkers, namely those containing nitrogen heterocycles such as azetidine or pyrroline derivatives, as well as variability in the number of carbon atoms comprising the linker. The PROTAC D6 was determined to have the most favourable antiproliferative activities, with a DC_50_ of 25.23 nM.^79^ This successful analog consists of a Niraparib warhead and a thalidomide E3 ligase ligand, connected by a linker consisting of two piperidine rings and alkyl spacers. A series of competition experiments utilising treatments with Niraparib, thalidomide, the proteasome inhibitor MG132 and an ML192 inhibitor yielded the blocked degradation of PARP1. It was also determined that D6 resulted in cell cycle arrest at the G2/M phase. A downregulation of the proteins CDK1 and CDC25C was identified through proteomics and western blot assays, further indicating the role of D6 in intercepting the CDC25C-CDK1 axis to disrupt key cell cycle transitions.^79^ The synthetic approach for Compound D6 is outlined in Scheme 4 and was achieved using the following steps. The previously synthesised intermediate compound **22** reacted with the aldehyde of compound **21** in a reductive amination to afford compound **23**. Following the second reductive amination of compound **23** with compound **21**, the resulting compound **25** was reacted with thalidomide 5-fluoride (compound **24**) in an initial deprotection and nucleophilic aromatic substitution to afford the PROTAC product Compound D6.

**Figure 7.**
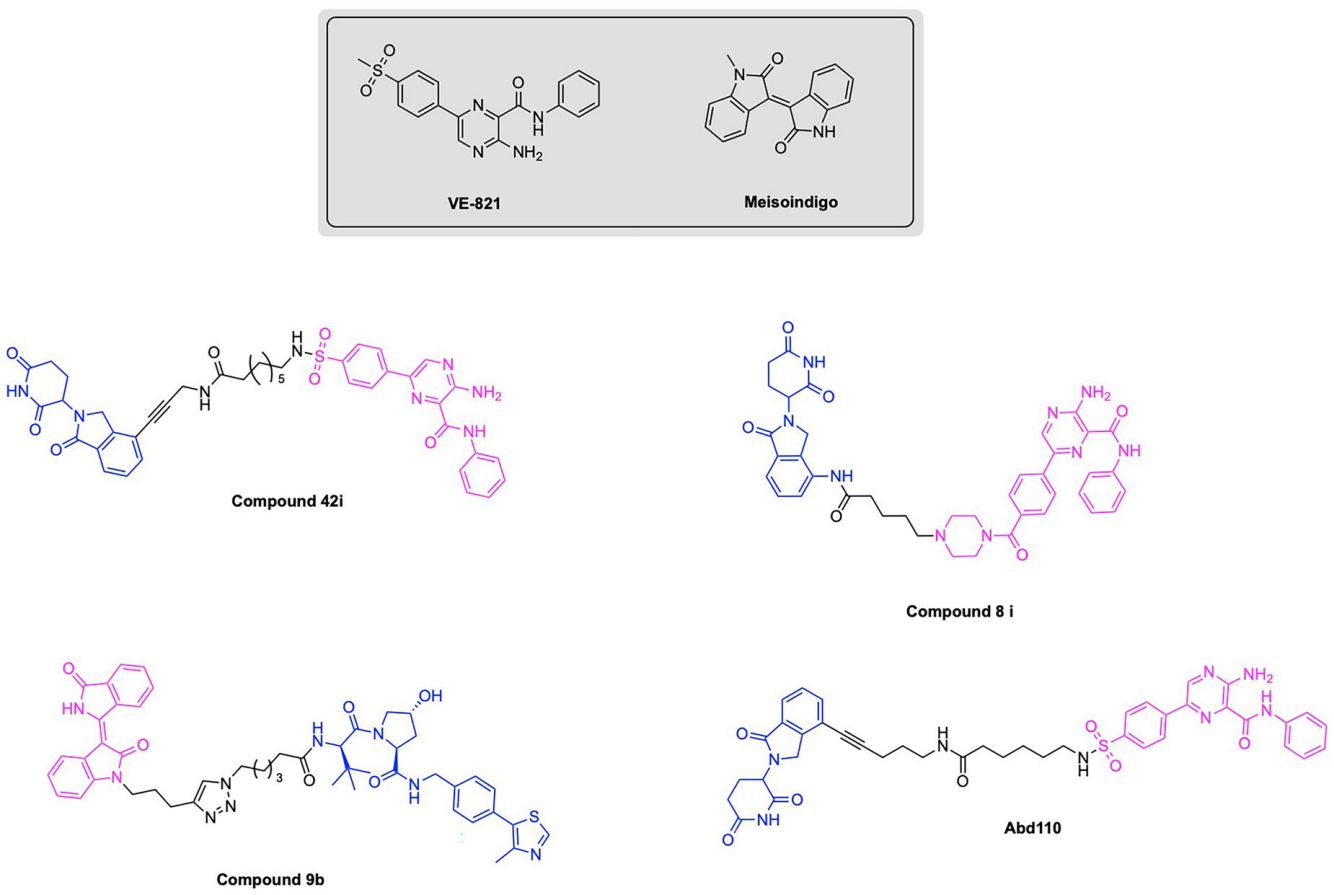
Overview of PROTACs targeting proteins in compensatory pathways for synthetic lethality induced by PARP degradation.

The establishment of PARP degrading PROTACs in literature is not only complemented by structural diversity in ligand and linker choice, but the discovery of novel mechanistic pathways by which these heterobifunctional molecules act. The effect of PARP degraders on inducing a non-apoptotic form of cell death known as ferroptosis was explored by Li et al. in their discovery of the PROTAC NN3 (Figure 6, Table 3).^81^ This PROTAC was designed to contain the PARP inhibitor Niraparib covalently attached to the MDM2 E3 ligase ligand Nutlin-3a through a PEG linker. An IC_50_ of 13.97 μM was achieved by this PROTAC in the MDA-MB-231 cell line.^81^ A series of competition experiments involving the treatment of cells with the proteasome inhibitor MG132 and the neddylation inhibitor MLN2924 revealed the dependence of the PROTAC NN3 on the ubiquitin proteasome system to cause the degradation of PARP1. NN3 also demonstrated greater potency than Niraparib in p53-positive cells, attributed to the activation of ferroptosis and p53 pathways by the degrader, as revealed by proteomics assays. A notable hallmark characteristic of ferroptosis includes lipid peroxidation and the accumulation of reactive oxygen species (ROS). To confirm the role of NN3 in inducing ferroptosis, the cell lines MDA-MB-453, MCF-7, MDA-MB-231 and MDA-MB-468 were studied to determine lipid ROS levels through the utilisation of BODIPY^®^ lipid probes, in which it was observed that lipid ROS levels were significantly increased in a dose-dependent manner due to NN3 treatment.^81^ Further advancements have been made in the incorporation of the PARP inhibitor Rucaparib for the development of novel PARP degraders. Chen et al. have developed the PROTAC 180055 through the covalent attachment of Rucaparib to a VHL ligand through a diverse range of linkers encompassing varying lengths of both PEG and alkyl-based structures (Figure 6, Table 3).^83^ A strategic approach was used to derivatize Rucaparib for attachment to the PROTAC through leveraging the solvent exposed secondary amine on the inhibitor for linker attachment. An evaluation of degradation efficiency in the cancer cell lines T47D and MDA-MB-231 revealed that the compound 180055 to have the greatest potency, with a DC_50_ of 180 nM and 240 nM in T47D and MDA-MB-231 cell lines respectively (Table 3).^83^ A series of competition experiments conducted through the addition of MG132 revealed the dependence of 180055 on the ubiquitin proteasome system, serving to confirm that this degrader is a PROTAC. It was also seen that the degradative properties of 180055 exclude chromatin trapping of PARP1, an undesirable consequence of PARP inhibitors resulting in DNA replication stress and eventual cell death.^93–95^ Additionally, a proteomics study showed that PARP1 was the sole target of 180055, with no degradation observed for other PARP family members such as PARP2 and Tankyrase.^83^ The selectivity of 180055 in enabling the degradation of solely PARP1 demonstrates advancements in the design and application of PARP targeting PROTACs, specifically for effective approaches in targeting specific oncogenic proteins within a protein family. The capabilities of PROTAC-based TPD have made significant progress in utilising a range of known PARP inhibitors to develop successful degraders. Despite the significant progress made in the expansion and optimisation of PARP-targeting PROTACs, limitations remain resulting from innate compensatory mechanisms that facilitate cell survival in the absence of the PARP protein. Cells maintain homeostasis using a highly interconnected network of DNA damage response pathways, encompassing methods of repair for both single-stranded and double-stranded breaks. When one repair axis is compromised, the upregulation of compensatory signalling networks facilitates DNA repair and preserves cell viability. In the context of cancer therapy, such mechanistic versatility serves to promote cell proliferation and tumour progression. The targeting of such mechanisms in tandem with PARP degradation can therefore amplify the therapeutic efficacy through synthetic lethality (Figure 7). Among the most prominent mediators in this compensatory pathway are the phosphatidylinositol 3-kinase related kinases ataxia telangiectasia mutated (ATM) and ataxia telangiectasia and Rad3-related (ATR).^96–98^ ATM is activated primarily by DNA double-stranded breaks and orchestrates both checkpoint activation and homologous recombination repair through the phosphorylation of substrates including BRCA1, p53 and CHK2. ATR is mobilised in the presence of replication stress and the accumulation of single-stranded DNA at stalled replication forks, resulting in CHK1 activation and the stabilisation of replication intermediates. Cooperatively, these kinases compose a multicomponent surveillance system that integrates damage detection and repair.

As noted earlier, the inhibition or degradation of PARP-family proteins imposes a profound replication stress by causing the accumulation of unrepaired single-stranded breaks that lead to cell death. In these circumstances, ATM and ATM assume compensatory roles, mitigating replication fork collapse and facilitating cell survival through checkpoint enforcement and the stabilisation of replication forks.^99–101^Such adaptive processes attenuate the cytotoxic potential of PARP inhibition, proving a mechanistic basis for therapeutic resistance in tumours.

Inherent limitations of PARP inhibition or degradation provide an opportunity for the TPD approach to be applied towards proteins involved in the described compensatory mechanisms. The concomitant inhibition of PARP and ATM or ATR with small-molecule agents has recently emerged as a compelling therapeutic strategy, underscoring the potential of synergistic combinations to overcome tumour cell proliferation persisting under PARP inhibition alone. The work of Lloyd et al has demonstrated that in ATM-deficient FaDu cells, the combined inhibition of PARP and ATR elicited striking synthetic lethality. Co-administration of 3 µM olaparib and 1 µM AZD6738 resulted in the highest observed cytotoxicity, achieving 84% cell death in the ATR-knockout cell line.^102^ Mechanistic investigation of this synergy demonstrated that Olaparib monotherapy induced G2-M checkpoint arrest through ATR-CHK1 activation, thereby permitting the occurrence of repair processes. In contrast, the dual inhibition abrogated this checkpoint, resulting in the start of mitosis prior to DNA repair.^103^ This occurrence was accompanied by hallmark features of mitotic catastrophe, most notably a pronounced increase in micronuclei formation. These findings align closely recent studies demonstrating that a range of inhibitors for ATM, ATR and WEE1 have combinatorial effects with PARP inhibitors, specifically Niraparib, Rucaparib and Olaparib. The ATR inhibitor Elimuserib and the ATM inhibitor AZD1390 demonstrated the most potential to increase anti-cancer effects and prevent the occurrence of compensatory biological responses.^104^ The field of ATR-targeting PROTACs was pioneered by the discovery of Abd110 (Ramotac-1), which integrates a derivative of the ATR inhibitor VE-821 with a lenalidomide-based CRBN ligand. Abd110 effectively induces ATR degradation across multiple leukemic cell types, with an IC50 of 17.3 nM obtained in primary leukemic cells isolated from a chromic myeloid leukaemia (CML) patient.^105^ The expansion of ATR-targeting PROTACs was further expanded by the work of Alfayomy et al, in the design of a series of VE-821 derived degrader having either CRBN or VHL ligands (Figure 7). Among these analogs, compound 42i emerged as the most potent with a reported IC50 value of greater than 10 µM from an in-vitro study.^106^ Wang et. Al similarly utilised a lenalidomide and VE-821 based architecture to develop the ATR degrader 8i (Figure 7). This PROTAC exhibited potent target engagement, with a DC_50_ of 22.9 nM in MV-4–11 cells and 34.5 nM in MOLM-13 cells. Notably, 8i exerted superior inhibitory activity in comparison to its parent VE-821 analog, having IC50 values of 0.108 µM versus 2.69 µM respectively.^107^ These examples illustrate a growing paradigm in which PROTAC-mediated TPD of kinases such as ATR and ATM can be harnessed to dismantle compensatory biological pathways preventing the synthetic lethality induced by PARP degraders. Although direct synergy between PARP and ATR or ATM degraders has yet to be systematically explored, emerging studies demonstrating successful PROTAC-mediated degradation of these kinases establish a conceptual foundation for future combination studies aimed at amplifying synthetic lethality.

### PROTACs for GPX4 degradation

#### Biology

The advantage of PROTAC based targeted protein degradation strategies is also evident in the utilisation of ferroptosis pathways to induce tumour cell death. Ferroptosis is a distinct form of regulated cell death that differs markedly from more commonly induced mechanisms for inducing tumour cell death, such as apoptosis and necrosis.^108^^,^^109^ The accumulation of iron along with lipid peroxidation are notable features of this form of cell death, along with mitochondrial reduction and the absence of key processes involved in apoptosis and autophagy.^11^ Ferroptosis is highly relevant to the onset and progression of many cancer types such as lung cancer, gastrointestinal cancers and pancreatic cancer, in addition to other pathologies.^110^^,^^111^ The induction of cancer cell death through promoting ferroptosis has become a prevalent strategy in recent years for preventing drug resistance.^112^ One particular enzyme, GPX4 has garnered attention as a crucial target due to its role in converting lipid peroxidation products that induce ferroptosis. This selenoperoxidase serves to catalyse the reaction of hydroperoxides through the reduction of glutathione, thereby preventing the occurrence of ferroptosis based cell death. The detoxifying function of GPX4 has proven to be of great relevance through strategies leveraging GPX4 overexpression and knockdown for the modulation of cell death as well as the lethality of known ferroptosis inducers.^113^ Inhibitors for this enzyme have yet to reach the clinic but several warhead structures have been studied for the covalent inhibition of the catalytic selenocysteine.^114^ PROTAC based degradation of GPX4 has thus emerged as a promising tool for regulating this fundamental component of ferroptosis pathways, given the challenges associated with achieving optimal binding at the active site of this enzyme.^115^^,^^116^

#### Synthesis

**Scheme 5.**
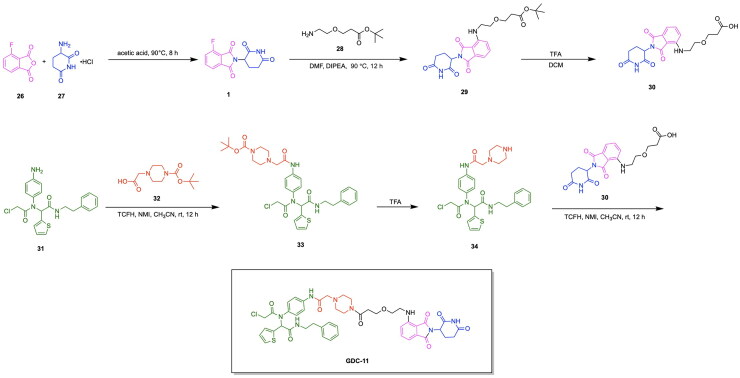
Synthesis Pathway for GDC-11 adapted from reference.^117^

**Figure 8.**
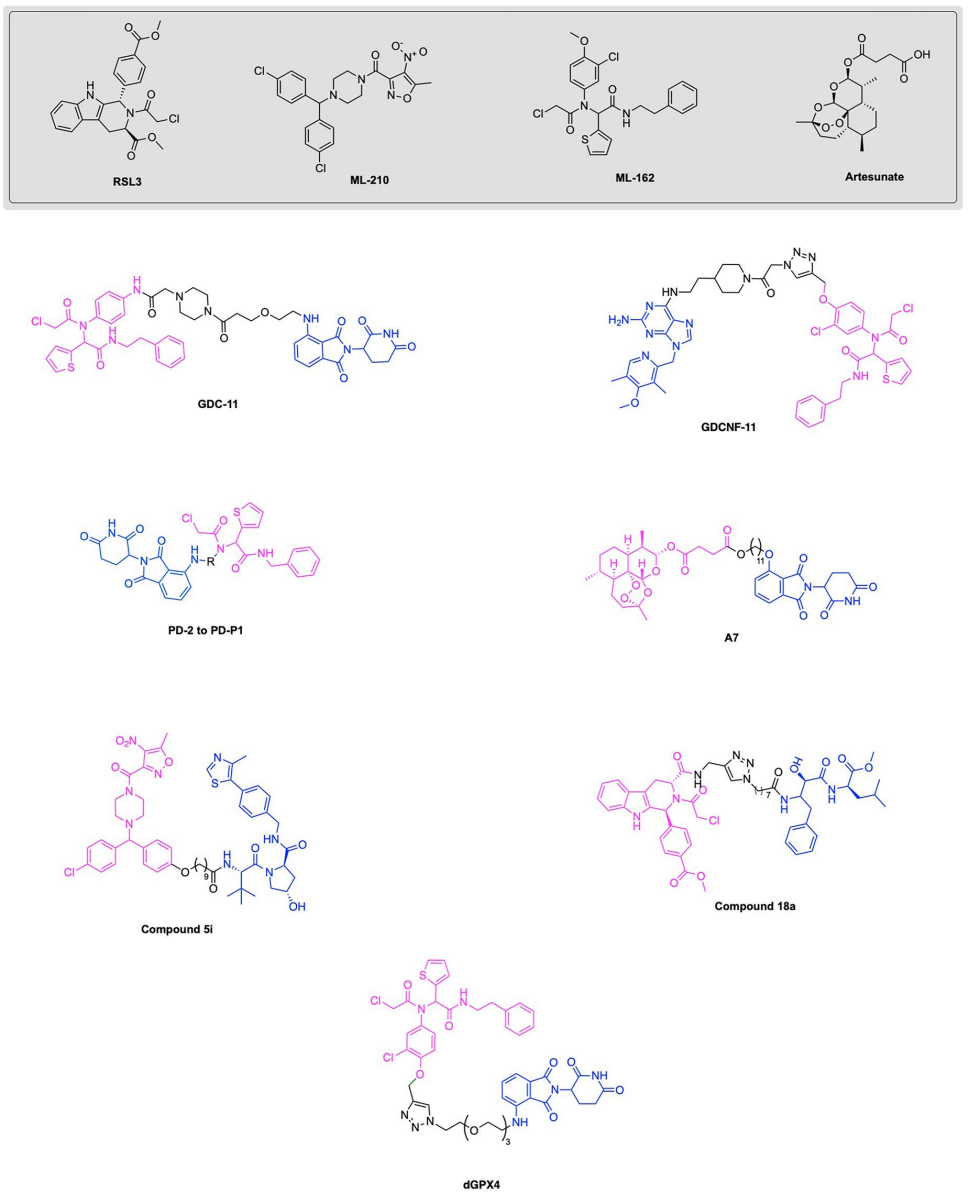
Overview of GPX4 degraders covered in the subsequent section on GPX4-targeting PROTACs..^117–123^

Notable progress has been made in leveraging known E3 ligase ligands in tandem with existing GPX4 inhibitors for inducing the degradation of GPX4 for promoting ferroptosis cell death. The emergence of GPX4 degraders was pioneered by Cai et al., who synthesised and evaluated various PROTAC designs using derivatives of the GPX4 inhibitor ML162 in combination with CRBN and VHL E3 ligase ligands, along with chemical optimizations of the linker structure.^117^ The importance of linker length for the degradative potential of developed PROTACs was demonstrated through the relatively superior degradation achieved by the VHL ligand-based PROTAC variants GDC-22∼GDC-24, consisting of a linker spacing of 8–10 atoms, along with pomalidomide based PROTAC variants GDC-5, GDC-9 and GDC-11 with a linker spacing of 12–13 atoms.^117^ They demonstrated that the optimal linker length in ML162-based PROTACs is contingent upon the specific E3 ligase ligand employed. GDC-22 emerged as the most potent PROTAC from this study, yielding 45% degradation of GPX4 at 10 μM concentration in a preliminary screening. However, the PROTAC variant yielding the most cytotoxicity was demonstrated to be GDC-11, with a degradation rate of 33% at 10 μM and IC_50_ value of 11.69 μM (Figure 8, Table 4).^117^ Lipid peroxide assays revealed that GDC-1 and GDC-11 resulted in the accumulation of lipid peroxides. Results of this study served to demonstrate that relatively high levels of GPX4 degradation do not inherently imply superior lipid peroxide buildup and cytotoxicity, providing insight into factors of GPX4 degradation important for inducing ferroptosis-based cell death. The synthesis of the PROTAC GDC-11 is shown in Scheme 5.^117^ The first step in the synthesis of the PROTAC GDC-11 involves an aminolysis reaction between 3-fluorophthalic anhydride (compound **26)** and 3-amino-2,6-piperidinedione (compound **27)** to afford 4-fluoro thalidomide (compound **1)**. Compound **1** was reacted with a tert-butyl ester-containing amine (compound **28**) to yield the pomalidomide analog compound **29**, which underwent deprotection by trifluoroacetic acid (TFA) to yield the carboxylic acid functionalised compound **30**. The GPX4 inhibitor ML162 (compound **31**) underwent condensation with the piperazine-based Boc-protected linker compound **32** to result in compound **33**, which was deprotected to produce compound **34**. Compounds **34** and **30** were then reacted via a TCFH coupling to produce the PROTAC GDC-11.

**Table 4.** Summary of Reported GPX4-Targeting PROTACs and Their In Vitro Potency Profiles.

PROTAC	POI	E3 Ligase/Binding Protein	Warhead	Linker Motif	Cell Line	In Vitro Potency/Affinity	References
GDC-11	GPX4	CRBN	ML162	PEG	HT-1080	IC_50_: 11.69 μM	112
GDCNF-11	GPX4	HSP90	ML162	Piperadine	HT-1080	IC_50_: 0.74 μM DC_50_: 0.08 μM	113
PD2-PD1	GPX4	CRBN	ML162	Alkyl/PEG	HT-1080	IC_50_ value for analogs: PD-2: > 10 μM PD-4: 0.86 μM PD-6: > 10 μM PD-P1: 3 μM PD-P2: 2.93 μM	114
A7	GPX4	CRBN	Artesunate	Alkyl	RT4, T24	IC_50_ in RT4 cells: 0.09 μM IC_50_ in T24 cells: 2.97 μM DC_50 _in RT4 cells: 475 nM DC_50 _in T24 cells: 1367 nM	115
dGPX4	GPX4	CRBN	ML162	PEG	HT-1080	IC_50_: 300 nM DC_50_: 200 nM	116
Compound 18a	GPX4	cIAP	RSL3	Alkyl	HT-1080	IC_50_: 2.37 μM DC_50_: 1.68 μM	117
Compound 5i	GPX4	VHL	ML210	Alkyl	HT-1080	IC_50_: 0.435 μM DC_50_: 0.135 μM	118

DC_50_, half-maximal degradation concentration; D_max_, maximal fraction of a target protein that is degraded; IC_50_, half-maximal inhibitory concentration; EC_50_, half-maximal effective concentration

**Scheme 6.**
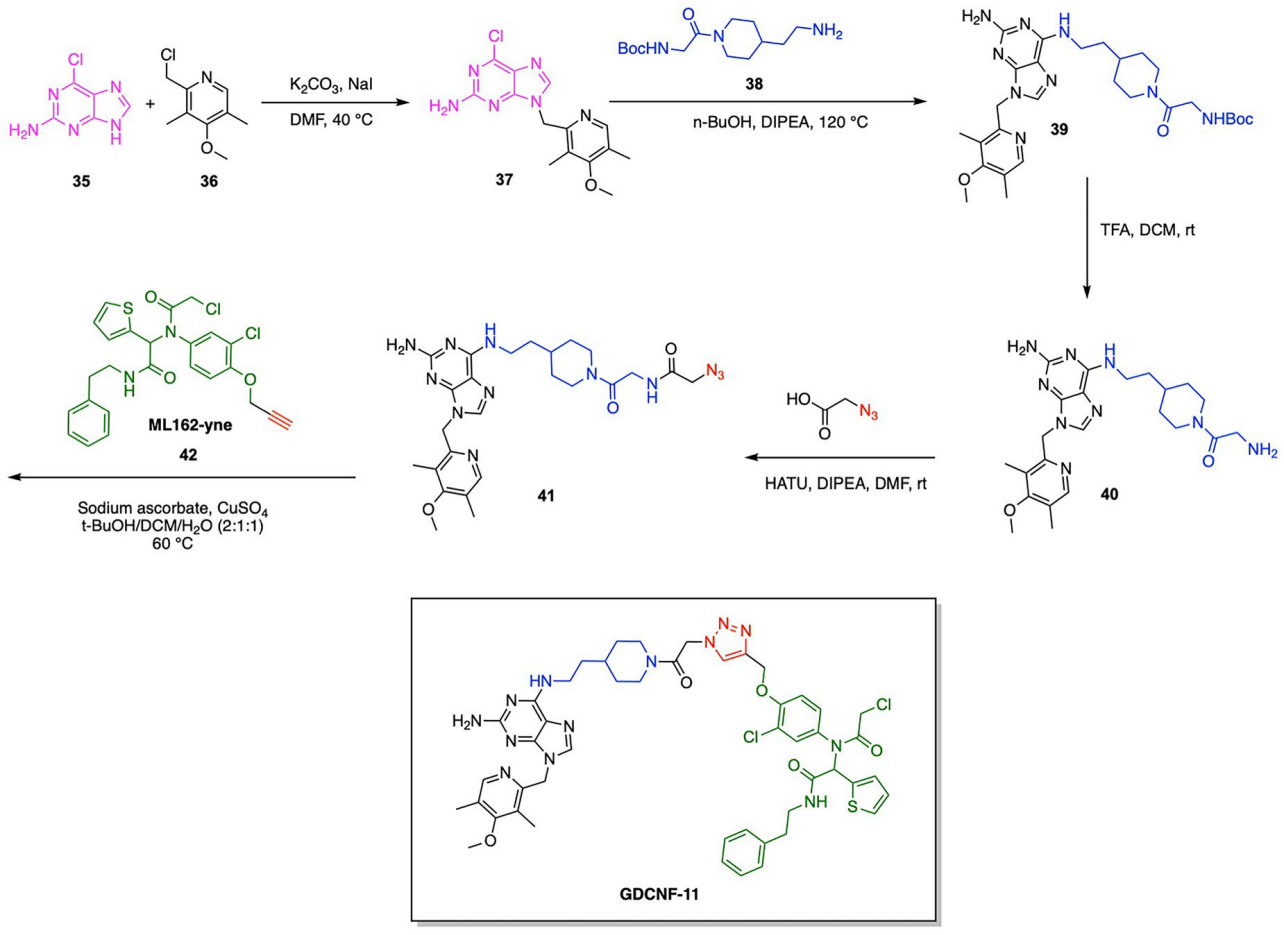
Synthesis Pathway of GDCNF-11 adapted from.^118^

The degradation of GPX4 using PROTACs has also been utilised in efforts to diversify the methods by which components of the ubiquitin proteasome system are utilised for the degradation of pathogenic proteins.^124^^,^^125^ Dong et al. developed a novel strategy for the degradation of GPX4 through the development of a PROTAC targeting heat shock protein for ubiquitin-proteasomal degradation.^118^ The developed heterobifunctional molecule termed as a HIM-PROTAC exhibited successful induction of ferroptosis cell death, as demonstrated by the successful variants GDCNF-2 and GDCNF-11.^118^ The molecular design of this HIM-PROTAC consists of the conjugation of the known GPX4 inhibitor ML162 to either of the reported HSP90 inhibitors PU-H71 and CNF-2024 through a diverse range of linkers such as polyethylene glycol chains, piperazine/piperidine chains and alkyl chains. Among the synthesised PROTACs, the top candidates, namely GDCNF-2 and GDCNF-11, exhibited DC_50_ values of 0.18 μM and 0.08 μM, respectively. Notably, GDCNF-11 demonstrated high ferroptosis selectivity, as evidenced by a substantial increase in its IC_50_ value from 0.79 μM to 35.5 μM upon co-incubation with a ferroptosis inhibitor (Figure 8, Table 4).^118^ A performed proteomics assay further exemplifies the highly selective nature of GDCNF-11 for less than 20 proteins, as compared to GDCNF-2 and other tested PROTAC variants. The synthesis of the PROTAC GDCNF-11 is depicted in Scheme 6.^118^ Compound **35** and compound **36** were covalently linked via a substitution reaction to afford compound **37.** Compound **37** was reacted with compound **38** in a nucleophilic aromatic substitution to afford compound **39**, which then underwent a deprotection reaction with TFA to afford compound **40**. Compound **40** reacted with azidoacetic acid in the presence of Hexafluorophosphate Azabenzotriazole Tetramethyl Uronium (HATU) and DIPEA peptide coupling reagents to afford compound **41**. Compound **41** was reacted with the GPX4 inhibitor derivative ML162-yne (compound **42**) in a copper-catalysed azide alkyne cycloaddition click chemistry reaction to result in the PROTAC GDCNF-11.

**Scheme 7.**
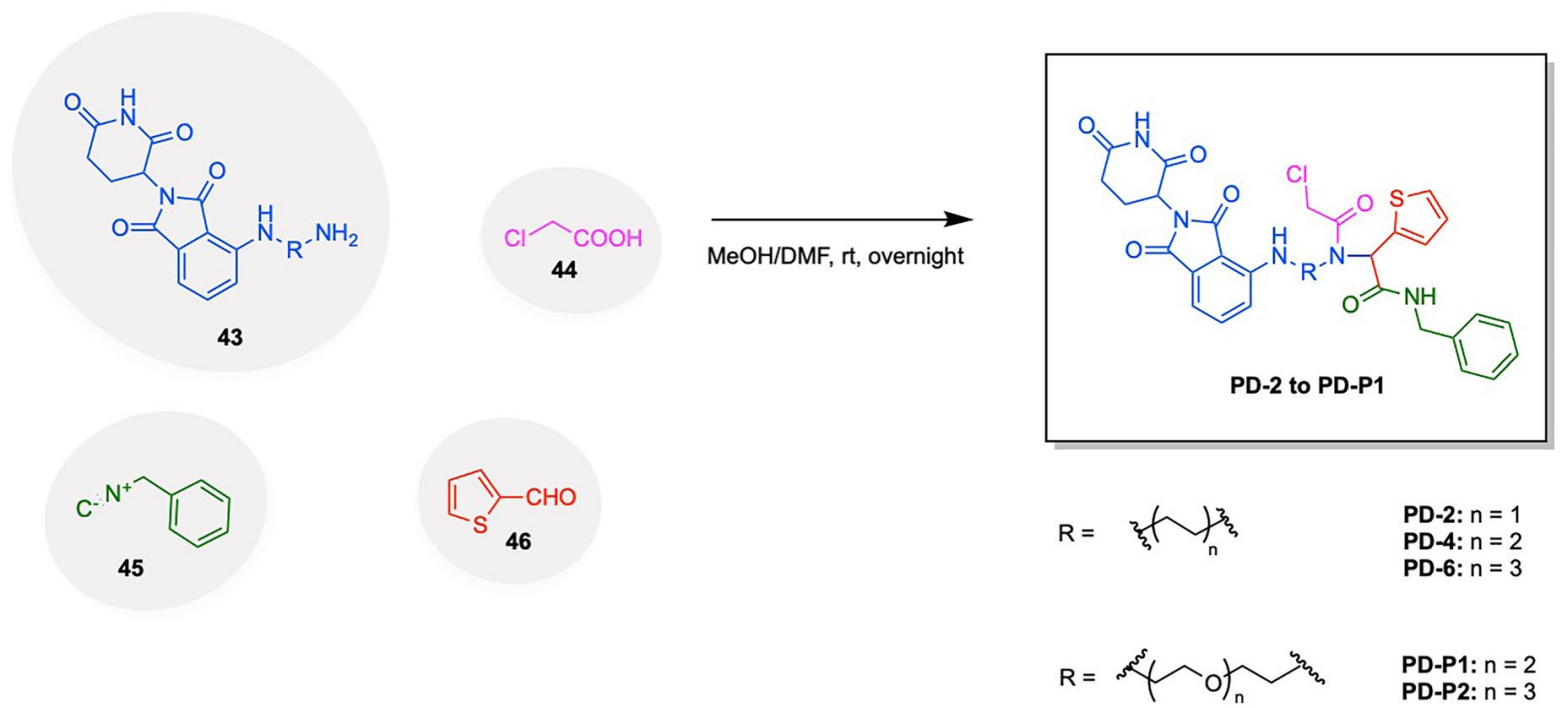
Synthesis Pathway of PD-2-PD-P1 adapted from reference.^119^

Given the notable progress in utilising PROTAC-based GPX4 degradation to mediate ferroptosis-induced tumour cell death, it is evident that these developments build upon prior synthetic advancements, facilitating the exploration of diverse PROTAC variations through linker and ligand modifications. Zhu et al. have highlighted the immense utility of the Ugi reaction, a multicomponent reaction, for the assembly of a range of ML162 and pomalidomide based GPX4 degraders with variations in linker type properties.^119^ The probe PD-Q2 was initially synthesised by conjugating the GPX4 inhibitor ML162 and pomalidomide to a clickable handle containing a 4-ethynylphenyl moiety, enabling click-chemistry based labelling and activity-based protein profiling (ABPP) of targeted proteins. To confirm GPX4 targeting by PROTACs, a probe-directed strategy was implemented to assess GPX4 activity following the conjugation to pomalidomide, thereby enabling the synthesis of additional PROTAC variants for further investigation.^119^ The synthesis of three PROTACs, PD-2, PD-4 and PD-6 with aliphatic linkers and two PROTACs PD-P1 and PD-P2 with a PEG linker was accomplished, with PD-4 and PD-P2 exhibiting optimal DC_50_ values of 0.13 μM and 0.21 μM respectively (Figure 8, Table 4).^119^ The synthesis of the developed PROTACs is shown in Scheme 7.^119^ The aldehyde compound **46**, the carboxylic acid compound **44**, the isocyanide compound **45** and compound **43** (a CRBN ligand with an amine functionality) were reacted in an Ugi one-pot reaction in an MeOH/DMF solvent system to afford the series of compounds PD-2, PD-3, PD-4, PD-P1 and PD-P2.

**Scheme 8.**
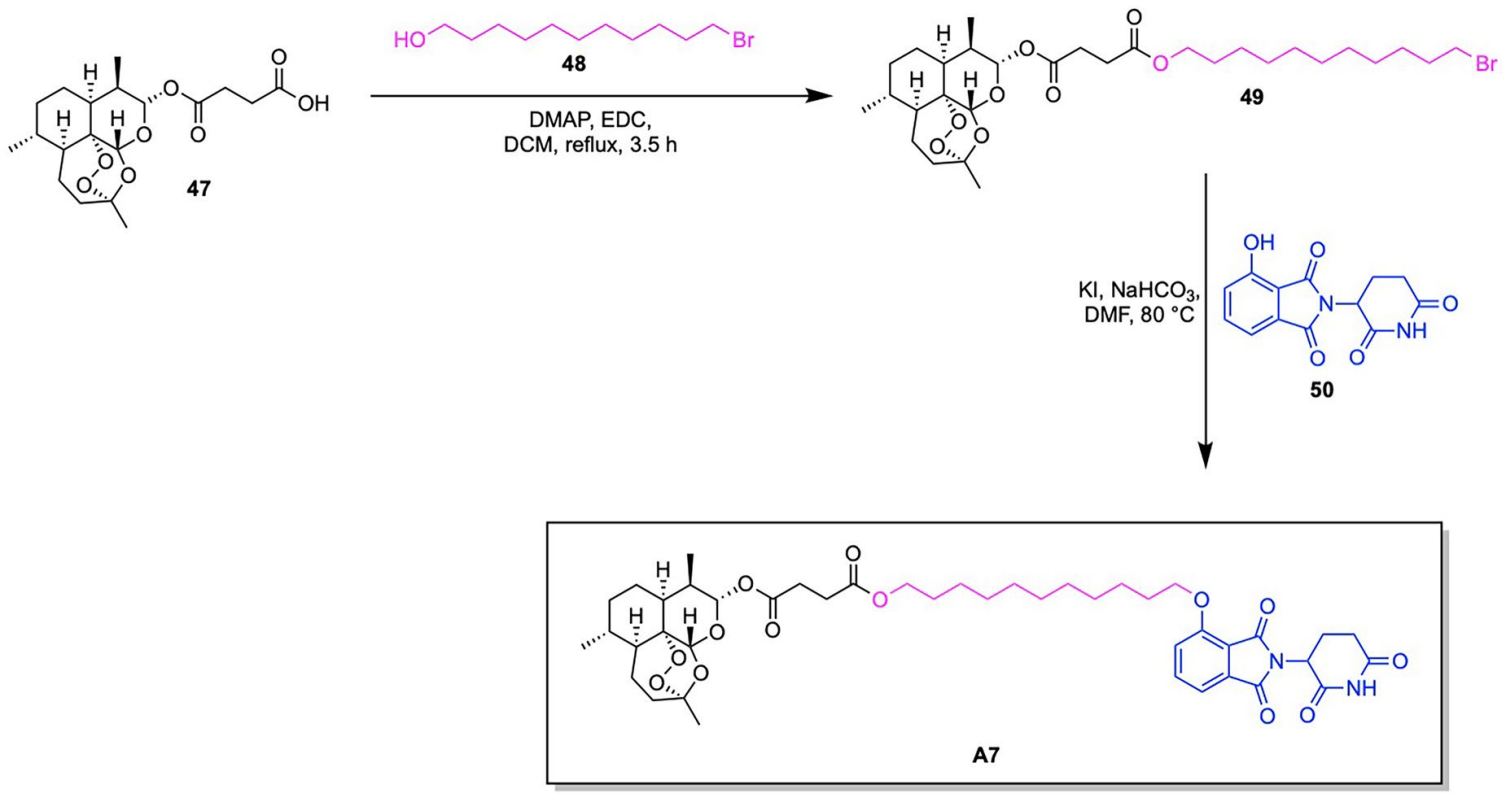
Synthesis Pathway of A7 adapted from reference.^120^

Recent innovations in the synthesis of GPX4 inhibitors and analogous PROTAC-based targeted protein degradation therapies seek to investigate novel GPX4 ligands amongst existing anti-cancer drugs and medicinal compounds available. Notable developments in GPX4 degraders include RSL3, ML162, ML210, JKE-164 and C18, which have been shown to induce ferroptosis in cancer cells.^126–129^ Leveraging existing therapies through the investigation of their potential to target GPX4 serves to provide benefits in expanding the chemical space of potential PROTAC ligands that can be utilised, while ensuring that the safety and bioactivity properties of these newly adopted ligands have been previously studied.^130–132^ Limitations associated with current therapeutic approaches in bladder cancer treatment in the form of adverse reactions and low effectiveness incentivise the utilisation of emerging targeted degradation therapies.^133^^,^^134^ These approaches aim to reduce toxicity and achieve therapeutic effects at relatively low concentrations than that of conventional small molecule treatments, thereby highlighting the ‘event-driven pharmacology’ approach that PROTACs use for protein degradation. Notably, recent studies have revealed that artesunate (ARS), a drug traditionally used to treat severe malaria, also exhibits anticancer activity. This highlights its potential for use in novel targeting protein degradation strategies for cancer treatment.^120^^,^^135^^,^^136^ Recent work by Yang et al. explores the utility of the anti-malarial drug artesunate (ARS) for inducing GPX4 degradation and ferroptosis in various bladder cancer cell lines by designing a series of ARS based PROTACs.^120^ Harnessing existing research on the role of ferroptosis in the anti-bladder cancer effects of ARS, the authors designed a series of ARS-based PROTACs. Among the synthesised variants, A7 emerged as a promising candidate (Scheme 8), demonstrating superior antiproliferative activity and GPX4 specificity through leveraging the ubiquitin-proteasome system to degrade GPX4 (Figure 8, Table 4).^120^ The design of PROTAC variants entailed the conjugation of ARS to either a VHL or CRBN E3 ligase ligand via a linker composed of alkyl or PEG chains of varying lengths, integrated across the different constructs. Among the synthesised variants, PROTAC A7, consisting of ARS conjugated to a CRBN ligand by an 11-carbon alkyl linker, exhibited the lowest IC_50_ value of 0.09 μM in the RT4 bladder cancer cell line.^105^ Although the PROTAC variant A8, consisting of a four-carbon alkyl chain, exhibited comparable IC_50_ values in the cell lines T24 and J82, A7 demonstrated relatively lower toxicity in the normal uroepithelium cell line SV-HUC-1, thus highlighting this variant as a potent and promising candidate. A performed western blot assay demonstrated the dose-dependent degradation of GPX4 in the RT4, T24 and J82 cell lines, with PROTAC sensitivity in these bladder cancer cells having a positive correlation with elevated levels of GPX4. The lipid ROS was measured through BODIPY-C11 staining, revealing the relatively higher ROS levels present following A7 treatment in comparison to ROS levels obtained from the ARS and control treatment. This is indicative of the role of A7 in promoting the occurrence of ferroptosis through the degradation of GPX4, a key detoxifier of lipid peroxides. The synthesis of PROTAC A7 is shown in Scheme 8.^120^ Compound **47** (Artesunate) was reacted with 11-bromo-1-undecanol (compound **48**) via a coupling esterification reaction in the presence of EDC/DMAP to afford compound **49**. Compound **49** was then reacted with compound **50** (4-hydroxy thalidomide) via a substitution reaction in the presence of potassium iodide and sodium bicarbonate to afford the PROTAC A7.

**Scheme 9.**
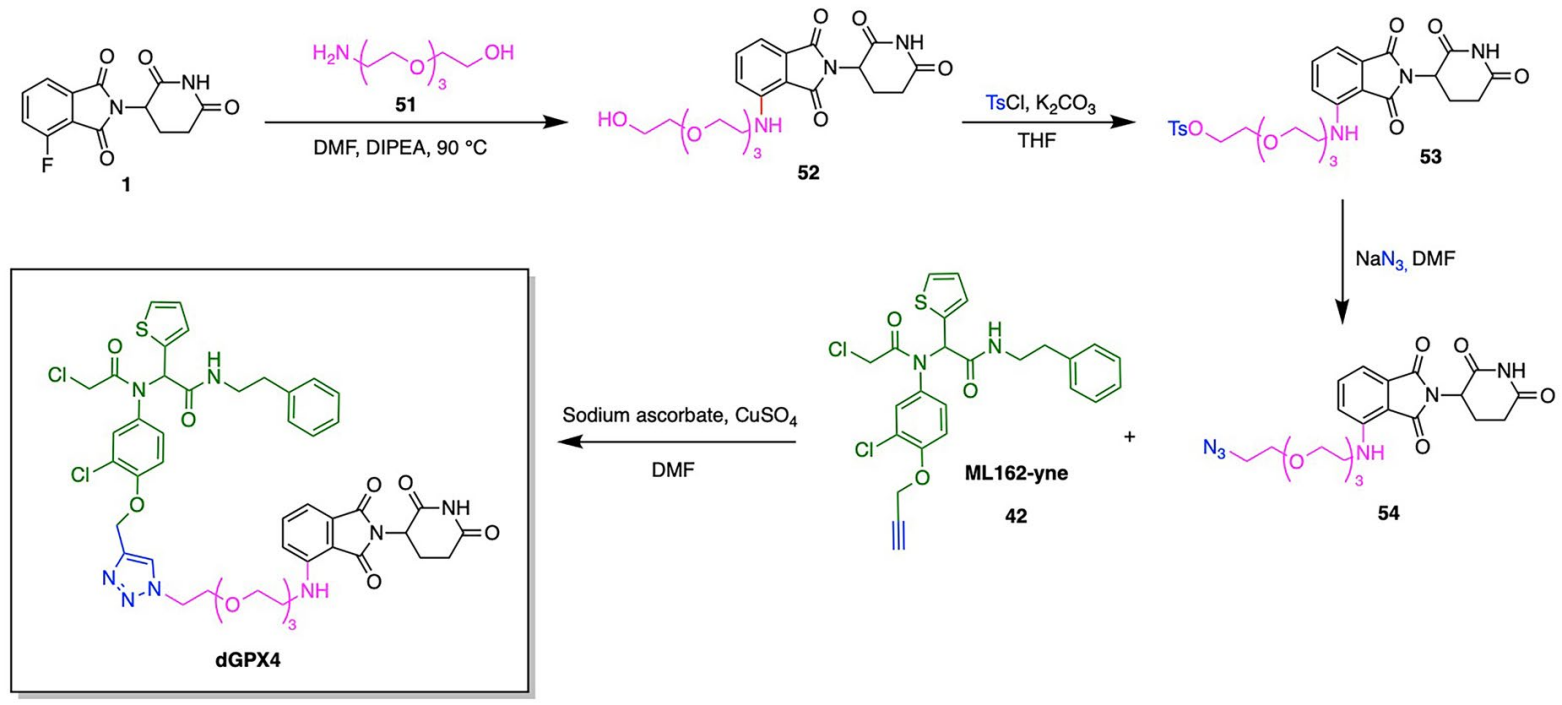
Synthesis Pathway of dGPX4 adapted from reference.^121^

Prior to the highlighted recent advancements in the development of GPX4 degrading PROTACs, application of PROTACs for GPX4 degradation have also been employed in tandem with emerging site-specific delivery systems, notably stimuli-responsive nanoparticles utilising factors such as pH, temperature and redox responsiveness for the site-specific delivery of therapeutics.^137–141^ Luo et al. have developed a PROTAC GPX4 degrader and facilitated the delivery of this molecule through utilising an ROS-responsive biodegradable lipid nanoparticle delivery system to enhance tumour targeting and cell selectivity.^121^ It can be noted that the work of this group served to pioneer the emergence of GPX4 degraders, as the developed GPX4-targeting PROTAC represents the first ever degrader of this protein. The authors report a five-fold increase in ferroptosis induction efficiency in comparison to the known GPX4 inhibitor ML162. The PROTAC was designed through the conjugation of ML162 to the CRBN E3 ligase ligand pomalidomide with a glycol linker having varying lengths. The dGPX4 PROTAC variant exhibited the highest potency, having an IC_50_ of 300 nM for HT-1080 fibrosarcoma cells, known to overexpress GPX4 (Figure 8, Table 4).^121^ A comparison with the IC_50_ value of 1.5 µM obtained for ML162 is indicative of the superior potency of the developed dGPX4 PROTAC. The presence of elevated ROS levels is a key indicator of the inhibition of GPX4 and the occurrence of ferroptosis due to the buildup of lipid peroxidation.^121^ The enhancement of intracellular ROS following dGPX4 treatment was confirmed through confocal laser scanning microscopy (CLSM) imaging and flow cytometry analysis of cells, following treatment with increasing concentrations of this PROTAC variant. Lipid peroxidation in the HT-1080 cell line was measured using BODIPY 581/591 C11 stain, which demonstrated an increase in fluorescence with a dependency on the concentration of dGPX4 added to the cells. The developed dGPX4 degrader was encapsulated in a lipid nanoparticle formation designed by the group, known as dGPX4@401-TK-12.^121^ To further validate the potency of this nanoparticle-encapsulated PROTAC, in-vivo studies confirmed that treatment in mice resulted in a 20% decrease in tumour size relative to what was observed in the PBS (phosphate-buffered saline) group.^121^ The synthesis of the PROTAC dGPX4 is shown in Scheme 9.^121^ 4-fluoro thalidomide (compound **1**) was reacted with amino-PEG3-OH (compound **51**) to afford compound **52** via a nucleophilic aromatic substitution. The obtained compound **52** was reacted with tosyl chloride to yield compound **53** which was subsequently reacted with sodium azide in DMF to yield compound **54**. The latter was reacted with ML162-yne (compound **42**) in a copper-catalysed azide alkyne cycloaddition click chemistry reaction to yield the PROTAC dGPX4.

The design and development of GPX4-targeting PROTACs incorporating the GPX4 inhibitor RSL3 have been advanced by Song et al. who report the synthesis and characterisation of Compound 18a (Figure 8, Table 4).^122^ This PROTAC demonstrated potent degradation activity in HT-1080 cells, achieving a DC_50_ of 1.68 μM and a maximum degradation of 85%. Antiproliferative activity was also observed, with an IC_50_ of 2.37 μM in the same cell line.

**Scheme 10.**
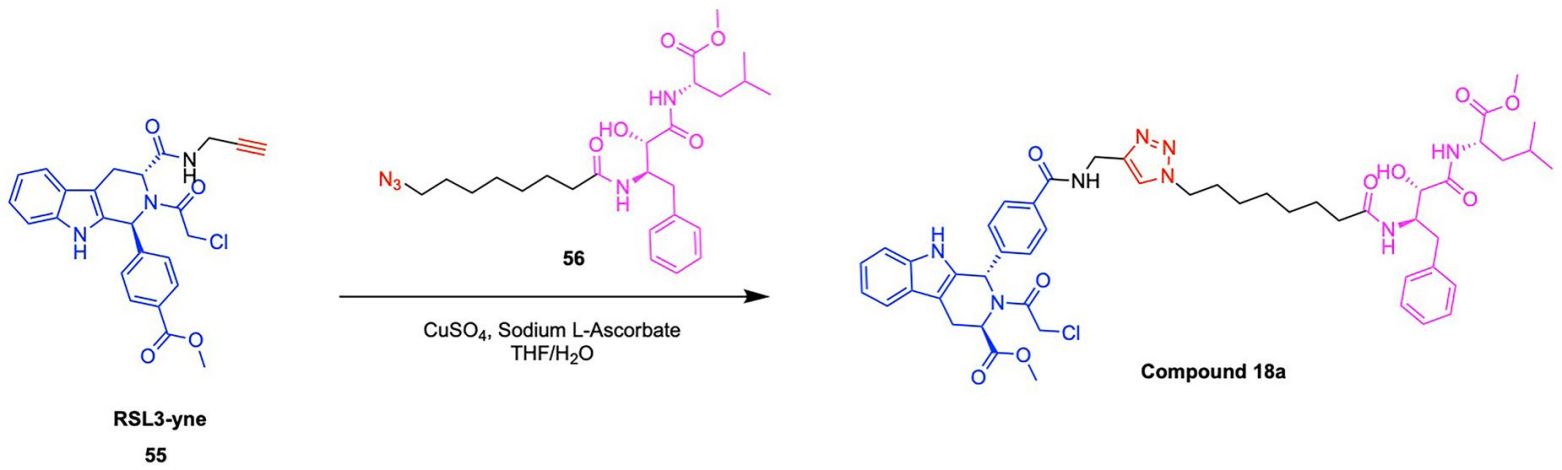
Synthesis of Compound 18a adapted from reference.^122^

A key innovation in the design of GPX4 degraders is demonstrated through the incorporation of bestatin as a ligand for recruiting the E3 ligase cIAP, representing a novel approach in GPX4 degrader design. The covalent linkage of RSL3 was achieved through the analysis of a crystal structure, revealing the solvent-exposed methyl carboxylate ester group and methyl benzoate group suitable for derivatization in the PROTAC design. A series of linker and E3 ligase ligand variations were employed through incorporating PEG and alkyl linkers of varying lengths, along with E3 ligase ligands for CRBN, VHL, MDM2 and cAIP.^122^ Among the synthesised analogs, compound 18a emerged as a potent candidate based on the superior degradation and antiproliferative profiles achieved in the HT-1080 cell line. Notably, analogs incorporating the cIAP-recruiting bestatin ligand exhibited enhanced antiproliferative activity compared to those employing alternative E3 ligase ligands, highlighting the efficacy of cIAP engagement in GPX4 degradation. Compound 18a showed minimal cytotoxicity in the HEK293T cell line in comparison to RSL3, indicating the improved selectivity associated with GPX4 degraders.^122^ A competition study was performed through treating cells with inhibitors of key UPS components, such as the proteasome, lysosome and E3 ligases. A decrease in degradation of GPX4 confirmed that Compound 18a acts through a PROTAC-mediated mechanism. The click chemistry reaction for Compound 18a is depicted in Scheme 10 and is as follows. The alkyne containing derivative of the GPX4 inhibitor RSL3 (compound **55**) was reacted in a copper catalysed azide alkyne cycloaddition reaction with an azide terminated cIAP ligand derivative (compound **56**) to afford Compound 18a.

The work of Hu et al. advanced the field of GPX4 degraders through the development of compound 5i (Figure 8, Table 4), employing the GPX4 inhibitor ML210 in a heterobifunctional PROTAC design.^123^ ML210 was selected as the POI ligand due to its superior *in vivo* performance compared to RSL3, as demonstrated in prior studies.^128^^,^^142^ A series of analogs were synthesised by covalently linking ML210 to a VHL ligand via various alkyl, PEG and conformationally restricted cyclic linkers of differing lengths. Derivatization of ML210 was achieved through a solvent exposed chlorophenyl group, identified via GPX4 crystal structure analysis.

**Scheme 11.**
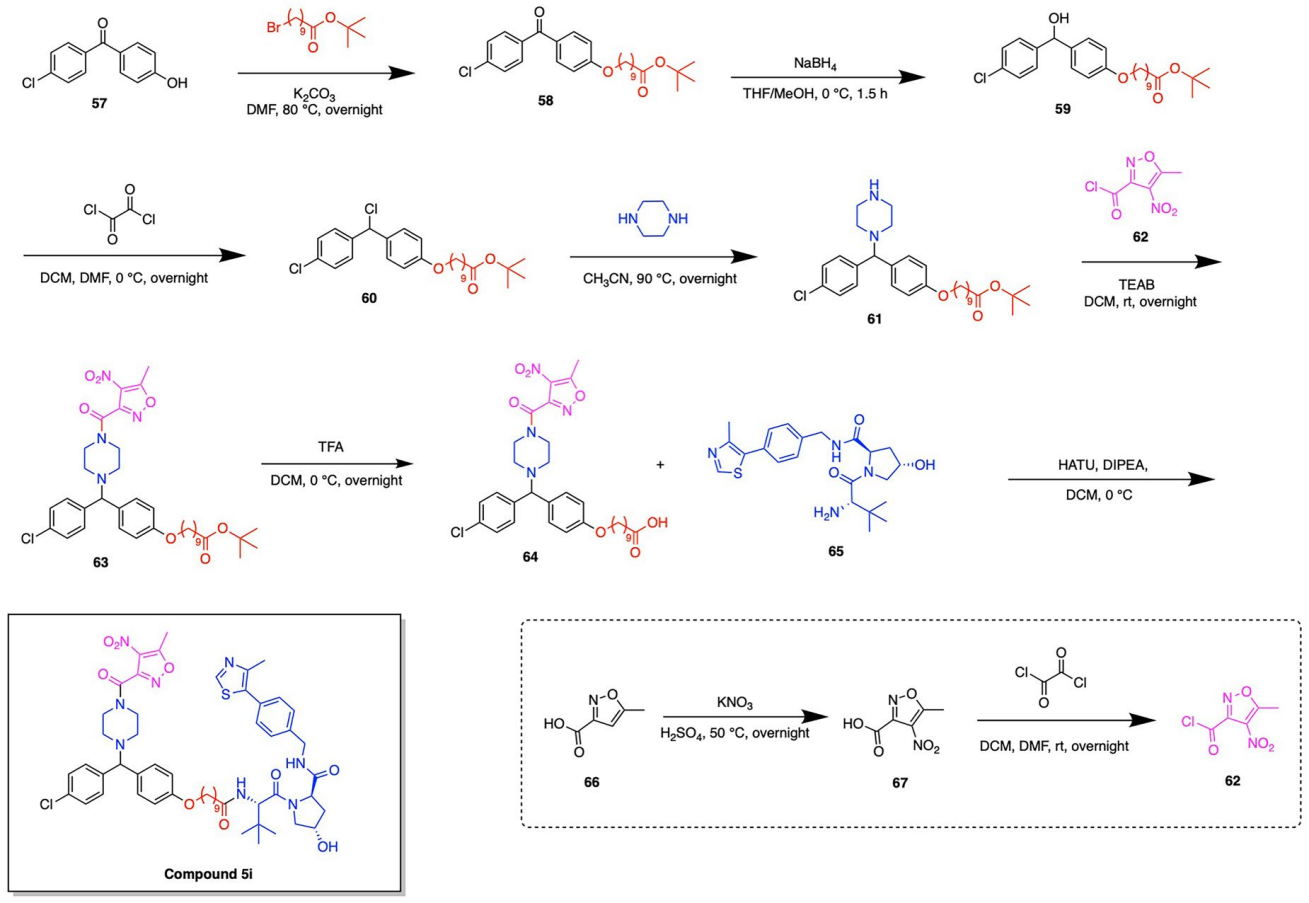
Synthesis route of Compound 5i adapted from reference.^123^

Compounds with PEG and cyclic linkers exhibited low potency, whereas those incorporating flexible alkyl chains showed improved antiproliferative activity. Among these, Compound 5i emerged as the most potent, with an IC_50_ of 0.435 μM and a DC_50_ of 0.135 μM.^123^ Competition assays using the proteasome inhibitor MG132 and the lysosome inhibitor chloroquine confirmed that GPX4 degradation was dependent on the UPS system, validating the PROTAC mechanism. The role of 5i in inducing ferroptosis was explored through ROS measurements in HT-1080 cells. Flow cytometric analysis of the DCFH-DA assay confirmed elevated ROS levels post treatment.^123^ Moreover, pre-treatment with ferrostatin-1 effectively suppressed ferroptosis, further confirming that compound 5i induces cell death through this pathway. The synthetic route for Compound 5i is outlined in Scheme 11 and is as follows.^123^ The compound (4-chlorophenyl)(4-hydroxyphenyl)methanone (compound **57**) was reacted with a Boc protected alkyl bromide in a Williamson ether reaction to afford compound **58**, which was subsequently reduced to afford compound **59**. Compound **59** was reacted with oxalyl chloride and piperazine to yield compound **61**. Compound **61** was reacted with the acyl chloride-containing compound **62**, which was previously synthesised from the nitration and acylation of the isoxazole derivative Compound **66**. Compound **63** was deprotected with TFA and reacted with the VHL ligand compound **65** to afford the PROTAC compound 5i.

#### Key takeaways

A persistent challenge in GPX4 inhibition and degrader design lies in balancing electrophilic reactivity with pharmacological stability and selectivity for the target protein. Covalent GPX4 inhibition is reliant on a binding interaction between electrophilic small molecules and the nucleophilic selenocysteine residue of this protein, potent inhibitors often rely on this interaction to achieve irreversible engagement.^114^^,^^143^ Nevertheless, pronounced electrophilicity can result in non-specific interaction with nucleophilic residues present in constituents of the cell proteome, resulting in off-target toxicity. Conversely, the presence of poor reactivity in GPX4 inhibitors may fail to engage the selenocysteine residue effectively, yielding poor degradation efficiency when incorporated into PROTACs. The field of GPX4 inhibitors was first pioneered by the Stockwell group, with the development of the compound RSL3, containing a highly reactive chloroacetamide warhead.^11^ Following this kickstart to the exploration of GPX4 inhibition, novel small-molecule inhibitors emerged, such as ML162 and ML210.^127^ The latter compound introduced the use of a masked nitrile oxide warhead, thereby providing a platform for tuning the reactivity towards the target protein. While the work of Weïwer et al. in 2011 first introduced ML210 and ML162, it was the work of Eaton et al. in 2020 that elucidated the mechanism of ML210 to reveal its workings as a prodrug.^128^ The utilisation of mass spectrometry revealed that the constituent nitro isoxazole moiety hydrolyses to form an α-nitro ketoxime intermediate, which subsequently dehydrates to a nitrile-oxide electrophile. This unmasked nitrile-oxide is then able to covalently react with the selenocysteine residue to GPX4 to irreversibly inhibit this enzyme. Notably, this inhibitor covalently binds to GPX4 solely under intracellular conditions, thereby exhibiting exceptional proteome selectivity. Insights from the investigation of GPX4 inhibitor reactivity hold immense potential in the fine-tuning of GPX4 degrader to enable greater selectivity. Nevertheless, caveats exist with the transition from POI inhibitor to degrader; factors such as ternary complex formation and protein-ligand affinities complicate the derivatization of ML210 to afford the same level of selectivity in comparison to other GPX4 inhibitors. Another important consideration in the biological assessment of GPX4 degraders is the interplay between GPX4 degradation and the consequent accumulation of lipid peroxides as a functional manifestation of ferroptosis stress.

Prior to introducing parallel antioxidant systems that constrain ferroptosis, it is important to delineate the upstream mechanisms that generate ferroptotic oxidative stress, with ferritinophagy-driven iron mobilisation serving as a central source of this event.

It can be noted that the generation of lipid radicals and lipid peroxides are due to both canonical processes resulting from metabolic functions of the mitochondria, the workings of lipoxygenases for immune responses, along with Fenton chemistry which may be the central driver of lipid peroxide accumulation.^144–152^

Cells continuously generate lipid radicals as a direct consequence of endogenous reactive oxygen species (ROS) production during mitochondrial metabolism. Electron leakage from the respiratory chain yields ROS that can propagate into surrounding cellular components, where they act as potent initiators of lipid oxidation.^153–155^ Components of the cellular membrane known as polyunsaturated fatty acids (PUFAs) are particularly susceptible to ROS-mediated attack, with arachidonic acid, linoleic acid, docosahexaenoic acid, and eicosapentaenoic acid being notable examples of substrates likely to undergo this process.^144^^,^^156^ Upon initiation, radical formation readily propagates along polyunsaturated fatty acid (PUFA) acyl chains, converting lipids into lipid peroxyl radicals and downstream peroxidation products that can compromise membrane integrity and downstream signalling.^144^^,^^146^^,^^157^^,^^158^ Secondly, lipid oxidation can arise through regulated enzymatic pathways, most prominently via lipoxygenases (LOX) which catalyse the stereoselective incorporation of molecular oxygen into PUFAs.^159–161^ In contrast to the largely stochastic, radical chain-driven lipid peroxidation initiated by diffuse ROS, LOX enzymes mediate controlled oxidation reactions that convert PUFAs into lipid hydroperoxides.^162^^,^^163^ The resulting oxidised lipids function as bioactive intermediates, serving as precursors for a diverse class of lipid mediators, including leukotrienes, lipoxins and related oxylipins.^146^^,^^164^^,^^165^ Consequently, LOX-driven PUFA oxygenation occupies a central position in the regulation of inflammatory tone, membrane-associated signalling, and immunometabolic communication, linking enzymatic lipid redox chemistry to coordinated cellular responses. Thirdly, the effects of iron-catalysed Fenton reactions, together with the size of the intracellular labile iron pool, constitutes a key mechanistic basis for lipid peroxidation and ferroptosis. Lipid peroxidation can be conceptualised as a three-stage process encompassing initiation, propagation and termination, encompassing the generation of lipid radicals, spread to PUFA-containing membrane phospholipids, and quenching by antioxidant systems respectively.^11^^,^^146^^,^^166–170^

Iron accelerates lipid peroxidation by promoting both radical initiation and chain propagation amplification. Mechanistically, this is driven by Fenton chemistry, in which redox-active iron catalyses the decomposition of hydrogen peroxide to generate highly reactive hydroxyl radicals, which attack membrane phospholipid PUFAs and generate the lipid peroxide chain reaction.^171^^,^^172^ Additionally, ferrous iron (Fe^2+^) can react with pre-existing lipid hydroperoxides (LOOH) to generate lipid alkoxyl radicals, thereby reinforcing chain-propagation reactions, amplifying lipid peroxidation, and exacerbating oxidative damage to membrane constituents.^173–176^ Collectively, these processes produce a net increase in lipid peroxide burden, necessitating detoxification by cellular antioxidant systems. The amount, subcellular distribution, and chemical speciation of intracellular iron are among the strongest determinants of lipid peroxidation propensity, as iron provides the redox activity required to initiate and amplify radical chain reactions.^169^^,^^177–179^ Fundamentally, most cellular iron is tightly sequestered within haem groups, and the iron storage protein ferritin, which collectively restrain iron-driven oxidative chemistry.^180^^,^^181^ The risk of lipid peroxidation is therefore governed primarily by the size and accessibility of the labile iron pool, comprising weakly-chelated iron species that can readily interconvert between Fe^2+^ and Fe^3+^ for participation in Fenton-type reactions.^182^^,^^183^ Perturbations that increase redox-active iron availability, including ferritin turnover, disrupted iron homeostasis, or impaired antioxidant defense, can consequently shift membranes towards heightened lipid peroxide accumulation and increase susceptibility to ferroptotic processes.^166^^,^^184^

Ferritin iron stores can be mobilised through ferritinophagy, a selective autophagy pathway that functions as a regulatory mechanism for expanding the labile iron pool (LIP). In this process, the cargo receptor NCOA4 (nuclear receptor coactivator 4) specifically recognises ferritin and promotes its delivery to the lysosome for degradation.^185^^,^^186^ Lysosomal turnover of ferritin then releases previously sequestered iron, increasing the availability of redox-active intracellular iron capable of participating in Fenton-type chemistry and thereby enhancing cellular susceptibility to iron-dependent lipid peroxidation. Accordingly, ferritinophagy provides a mechanistic link between iron homeostasis and oxidative membrane damage by controlling the flux of iron from inert storage complexes into the labile iron pool (LIP) for availability in peroxidation processes.^187–190^ A key component of expanding the LIP via ferritinophagy is the degradation of ferritin via selective autophagy, a process mediated by the workings of NCOA4, along with a host of proteins crucial for the formation of this vesicle and engulfment of ferritin. Structurally, ferritin is a hollow 24-subunit protein nanocage assembled from two core components: ferritin heavy chain (FTH1), which possesses ferroxidase activity that oxidises Fe^2+^ to Fe^3+^ to facilitate safe storage and ferritin light chain (FTL), which contributes to the structural stability and mineralisation of the ferric core.^191–193^ The mobilisation of ferritin-bound iron is nevertheless essential under multiple physiological and pathological conditions, including demands for cell division processes, support of mitochondrial metabolism, adaptation to iron scarcity and metabolic changes associated with oncogenic growth.^186^^,^^194^^,^^195^ The process of ferritinophagy is comprised of the enclosure of ferritin within a double-membrane vesicle known as the autophagosome, which subsequently fuses with lysosomes to enable cargo degradation.^196^ In this process, NCOA4 binds ferritin complexes and targets them for incorporation into developing autophagosomes, ultimately delivering ferritin to lysosomes for proteolysis and subsequent iron release. Consistent with this role, the loss of NCOA4 disrupts ferritin activity to autophagic membranes, resulting in ferritin accumulation and reduced mobilisation of labile iron.^197^^,^^198^ The mechanistic basis of selective autophagy is rooted in cargo receptor proteins that couple the substrate recognition process to the autophagy machinery.^199–201^ Accordingly, autophagosomal machinery is decorated by the autophagy-related protein 8 (ATG8) family proteins, including microtubule-associated protein 1 light chain 3 (LC3A/B/C) and the γ-Aminobutyric acid receptor-associated protein (GABARAP) subfamily, which act as docking sites for cargo receptors via LC3-interacting motifs. During autophagosome biogenesis, cytosolic LC3-I undergoes conjugation to phosphatidylethanolamine (PE) to form the membrane-associated LC3-II. This modification is crucial for autophagosome membrane elongation, phagophore expansion, and efficient sequestration of receptor-bound cargo.^202–204^ This LC3 lipidation process is executed by the canonical autophagy conjugation cascade, involving the E1-like enzyme ATG7, the E2-like enzyme ATG3 and the ATG5-ATG12-ATG16L1 complex, which together promote LC3 attachment to PE. These interactions enable the effective tethering of NCOA4-bound ferritin to nascent autophagosome membranes, and subsequent sequestration for lysosomal degradation.^205^^,^^206^ The genetic disruption of core autophagy genes such as ATG5 or ATG7 impairs LC3 lipidation and autophagosome maturation, resulting in reduced labile iron release and attenuated sensitivity to lipid peroxidation and the occurrence of ferroptosis.

Beyond genetic perturbations in ATG5 or ATG7, autophagy defects that reduce the occurrence of ferritinophagy arise from disruptions across the LC3 lipidation cascade, including compromised activity of the ATG5-ATG12-ATG16L1 conjugation complex or impaired upstream enzymatic steps in the conjugation cascade.^187^^,^^207^^,^^208^ Disruption of LC3 lipidation can arise from several events in the ATG12-ATG5-ATG16L1 conjugation system. Formation of the ATG12-ATG5 conjugate depends on the ubiquitin-like enzyme cascade driven by ATG7 and ATG10. Therefore, disruptions that reduce ATG5 or ATG12 abundance and impair or destabilise the resulting conjugate serve to reduce LC3/GABARAP lipidation.^172^^,^^205^^,^^209^ In addition, defects in the ATG16L1 expression impair recruitment of the ATG12-ATG5 conjugation machinery to the phagophore, reducing LC3/GABARAP lipidation and consequently decreasing LC3-II docking sites required for selective ferritin capture.^209^ Beyond genetic lesions to the ATG-family genes, post-translational regulation of ATG5 and ATG16L1 can further tune complex abundance and activity, linking autophagy flux to broader signalling and stress-response pathways.^206^^,^^210^ Notably, ferritinophagy can also be selectively modulated upstream of core autophagy execution by proteins that regulate cargo receptor stability and lysosomal trafficking, including HERC2 (HECT and RCC1-like domain-containing protein 2), TAX1BP1 (Tax 1 binding protein 1), and COPZ1 (Coatomer protein complex I subunit zeta I).^211^^,^^212^ The E3 ubiquitin ligase HERC2 functions as an iron-responsive regulator of ferritinophagy by targeting NCOA4 for proteasomal degradation under iron-rich conditions, thereby limiting ferritin degradation and increase to the LIP.^184^^,^^212^ Recent mechanistic studies suggest that NCOA4 possesses iron-sensing features, including iron-sulfur cluster-dependent conformational states enabling selective recognition by HERC2, thereby affirming the link between ferritinophagy flux to intracellular iron status.^213^^,^^214^ From a translational perspective, HERC2 represents a desirable upstream therapeutic target for both the control of ferritinophagy and the advancement of PROTAC design. Despite the advances in exploring E3 ligase pathways and targeting, direct and selective small-molecule modulation of HERC2 remains comparatively underdeveloped.^215–217^

In parallel, the selective autophagy adaptor TAX1BP1 (Tax 1 binding protein 1) has been identified as an essential NCOA-interacting factor that promotes lysosomal trafficking of ferritin. TAX1BP1 binds directly to NCOA4 and promotes the delivery of NCOA4-ferritin complexes to degradative compartments, thereby coupling ferritin substrate recognition to membrane trafficking and ferritin sequestration processes.^211^ TAX1BP1 has been reported to be essential for efficient ferritin trafficking under basal and iron-depleted conditions and has been implicated in an alternative lysosomal targeting route that is independent of the ATG8 conjugation (LC3/GABARAP lipidation) machinery, distinguishing it from canonical autophagy. This indicates that ferritin turnover is also modulated by processes for cargo sorting, recruitment, and delivery to lysosomes, with TAX1BP1 serving as an auxiliary determinant of ferritinophagic iron flux. Collectively, these examples support a multilayered regulatory framework in which ferritinophagy is influenced by both upstream determinants of iron availability that govern NCOA4 stability and degradation, along with downstream trafficking adaptors, creating multiple points at which the LIP and susceptibility to iron peroxidation can be modulated. Highlighting an additional regulatory layer in the occurrence of ferritinophagy, Wu et al. Have recently demonstrated that COPZ1 (Coatomer Protein Complex I subunit zeta 1) is linked to ferroptosis regulation in lung adenocarcinoma through the control of ferritinophagic iron mobilisation.^218^ Canonically, COPZ1 is a component of the coatomer protein complex I (COPI) vesicle coat machinery that governs intracellular cargo trafficking and organelle homeostasis, and has been implicated in membrane flux events that support autophagosome biogenesis, vesicular maturation, and lysosomal delivery, thereby positioning it to modulate selective autophagy pathways.^219–221^ In lung adenocarcinoma clinical datasets and patient specimens, COPZ1 expression is elevated and correlates with higher tumour grade and poorer survival, whereas NCOA4 expression is reduced, consistent with the suppression of ferritinophagy in cancer. It was discovered that COPZ1 silencing in lung adenocarcinoma and xenografts increases ROS, Fe^2+^ accumulation and lipid peroxidation products, increases mitochondrial shrinkage typical of ferroptotic stress, and limits tumour growth.^218^ These noted effects were observed to be reduced by ferroptosis inhibition using liproxstatin-1 or iron chelation using deferoxamine. Co-immunoprecipitation experiments indicated that COPZ1 associates with NCOA4, while COPZ1 knockdown and immunofluorescence co-localisation experiments further indicated that COPZ1 restrains NCOA4-dependent ferritin turnover. COPZ1 knockdown was observed to elevate NCOA4 and LC3II/I, thus promoting lysosomal targeting and ferritin degradation, along with increasing labile iron and lipid peroxidation.^218^ In contrast, NCOA4 depletion restores ferritin heavy chain 1 (FTH1) abundance and attenuates iron-associated oxidative damage. Although the precise mechanism by which COPZ1 controls NCOA4 abundance remains to be defined, these findings position COPZ1 as a tumour-supporting suppressor of ferritinophagy that limits labile iron release and thereby enables lung adenocarcinoma cells to evade ferroptosis. Lipid peroxide formation has been established as a convergent outcome of both basal mitochondrial ROS production, regulated PUFA oxygenation, and iron-catalysed radical chemistry. Amongst these drivers of oxidative stress, the availability of redox-active iron emerges as a dominant contributor to lipid peroxide formation, as Fe^2+^ both initiates lipid radical generation through Fenton reactions and propagates chain reactions through the decomposition of lipid hydroperoxides. Importantly, ferritinophagic flux is not solely dictated by core autophagy machinery but is modulated across multiple regulatory layers, including LC3/GABARAP lipidation and upstream factors controlling NCOA4 stability and lysosomal cargo routeing such as HERC2, TAX1BP1 and COPZ1. This multilayered network governing labile iron availability therefore defines the magnitude and persistence of oxidative stress that must be counterbalanced by antioxidant defense systems, defining the importance of GPX4-dependent detoxification processes along with parallel redox buffering pathways discussed in the subsequent section.

Although GPX4 is well established as a central regulator of ferroptosis through its role in lipid peroxide detoxification, parallel antioxidant pathways also constrain lipid peroxide accumulation, rendering the relationship between GPX4 inhibition and lipid peroxidation nuanced. While GPX4 serves as the primary enzymatic safeguard against ferroptosis, its inhibition doesn’t invariably result in excessive lipid peroxide accumulation and cell death. The existence of GPX4-independent antioxidant systems work to maintain redox homeostasis when GPX4 has been compromised by inhibition or degradation. A key compensatory mechanism is the FSP1-CoQ10 axis. The protein FSP1, originally known as AIFM2,^222^^,^^223^ functions independently of GPX4 by catalysing the NAD(P)H-dependent regeneration of the reduced CoQ10, a potent lipophilic radical-trapping antioxidant. This process enables the increase in intracellular CoQ10 levels, thereby preventing the propagation of lipid peroxide accumulation and preventing ferroptosis cell death. The overexpression of FSP1 has the potential to robustly suppress ferroptosis induced by GPX4 inhibition, underscoring its role as a compensatory mechanism of preventing lipid peroxide accumulation. A similar detoxifying agent known as dihydroorotate dehydrogenase (DHODH) is present in mitochondria.^224^ Beyond this enzyme’s canonical role in pyridine synthesis, DHODH enables the generation of reduced CoQ10 within the mitochondrial inner membrane. This serves to mitigate lipid radical formation, thus establishing this enzyme as a ferroptosis defense module acting independently of GPX4. Collectively, these parallel systems indicate that lipid peroxide levels are not a direct functional readout of GPX4 degradation due to the multilayered effects that parallel detoxification pathways entail towards the prevention of ferroptosis. The biological evaluation of lipid peroxide levels has been extensively studied in the evaluation of developed GPX4 degraders, and the compensatory pathways described should serve as an important consideration in assessing GPX4 degrader potency. The elucidation of the mechanisms by which redox homeostasis is maintained holds great promise in the implementation of synergistic protein inhibition and degradation strategies that more effectively induce ferroptosis.

In addition to compensatory detoxification mechanisms that preserve redox homeostasis when canonical GPX4 activity is diminished, it is important to note that ferroptosis susceptibility is also governed by upstream metabolic factors that influence lipid biosynthesis and thereby, the availability of oxidisable PUFAs. In this context, broader metabolic regulators outside canonical lipid peroxide detoxification mechanisms may indirectly influence vulnerability to ferroptosis by remodelling the flux of lipid synthesis.^225–227^ A recent review by Sun et al. reports cannabinoids to engage metabolic signalling pathways such as AMP-activated protein kinase (AMPK), a cellular energy sensing protein that regulates lipid biosynthesis through the inhibition of acetyl-CoA carboxylase (ACC).^228^ AMPK-dependent suppression of PUFA biosynthesis can reduce the pool of peroxidation-prone lipids contributing to ferroptosis, thus illustrating how upstream metabolic states may modulate ferroptosis sensitivity in a context-dependent manner.

### PROTACs for epigenetic regulator degradation

#### Biology

The emergence of PROTAC based protein degradation for inducing tumour cell death has also been extensively applied towards cancers originating from mutations of epigenetic regulators. DNA and histone acetylation and methylation are dynamic chemical modifications that govern patterns of gene expression through the functionality of a range of proteins, namely writers, and erasers.^229^^,^^230^ The localisation of these proteins is dependent on reader proteins that recognise histone and DNA modifications. The activity of such proteins involved in epigenetics serves to enable the modulation of chromatin structure and dynamic changes in gene expression, notably the expression of tumour suppressor genes and proto-oncogenes.^231^ The occurrence of mutations in proteins regulating epigenetic modifications has become a prominent focus in the treatment of cancer, particularly regarding the bromo and extra-terminal domain (BET) family of proteins, notably BRD4, histone deacetylase (HDAC), and enhancer of zeste homolog 2 (EZH2).^232–234^ Aligning with the general incentive for PROTAC based treatment approaches, the driving force to facilitate targeted protein degradation of these key proteins in epigenetic regulation lies in the modest clinical activity exhibited by current small molecule inhibitors along with the opportunity to use sub-stoichiometric doses for targeted protein degradation.^235^^,^^236^

In tandem with the targeting of epigenetic regulator proteins through PROTAC mediated degradation, emerging computational strategies have advanced the understanding of the network of enhancer and epigenetic modification protein interactions that are a hallmark of cancer proliferation, thereby enabling the identification of key regulatory pathways and processes amendable to therapeutic intervention.^237–239^ The degradation of proteins involved in chromatin remodelling and transcription remains a promising method of treating cancer due to the impact such proteins have on sustaining oncogenic states that drive cancer proliferation.^240^^,^^241^ Through the elimination of processes silencing tumour suppression, promoting continuity of the cell cycle, and the activation of metabolic programs, targeted degradation of these proteins can disrupt oncogenic transcriptional networks and thereby facilitate cancer cell death, as noted in the examples provided in this review.^242–247^ While PROTACs targeting such epigenetic regulators demonstrate potent anticancer effects, their efficacy is closely tied to the underlying control of transcription and chromatin structure through an interconnected network of proteins.^235^^,^^247–253^ Emerging computational strategies are therefore critical for analysing enhancer-protein networks and predicting suitable targets for PROTAC -mediated protein degradation. Enhancers, defined as noncoding DNA elements that promote the transcription of target genes, play central roles in sustaining oncogenic transcription programs in cancer. The binding of regulatory proteins (transcription factors) in response to extracellular signals to this platform, followed by the recruitment of co-activators and co-repressors serve to create an integrated regulatory cue that enables cell-specific gene expression.^238^^,^^254–256^ Enhancers fine tune the extent and timing of gene expression, and their dysregulation can contribute to tumorigenesis through driving the aberrant overexpression of oncogenes.^254^^,^^256^

Enhancer landscapes are critical for establishing and maintaining cell identity.^239^^,^^254^^,^^256^^,^^257^ In cancer, the emergence of super-enhancers at oncogenic loci, characterised by dense clusters of enhancers with an unusually high occupancy of transcriptional regulatory proteins, can hijack normal gene regulatory programs.^239^^,^^258–261^ Such changes to enhancer architecture drives the overexpression of oncogenes, leading to sustained promotion of uncontrolled cell proliferation, altered differentiation states, and enhanced cell survival.^238^^,^^258^^,^^262^

Several canonical examples of super-enhancers across both solid and haematologic malignancies have been identified, that converge on the reprogramming of normal regulatory networks into oncogenic programs. The MYC oncogene is a notable example of a cancer driving genetic element driven by super enhancers, wherein dense assemblies of transcription factors and coactivators concentrate at the MYC locus to enforce persistent expression and thereby cancer proliferation, biomass accumulation and survival.^254^^,^^256–258^^,^ MYC encodes the cellular MYC transcription factor (c-MYC) which functions as a central regulator in oncogenesis, exhibiting its multifaceted impact on cancer through the promotion of uncontrolled proliferation, boosting cellular metabolism, and preventing terminal differentiation, thereby resulting in the maintenance of an oncogenic cell identity.^263^ Amongst the range of super enhancer networks resulting in aberrant MYC expression, one example is present in T-cell acute lymphoblastic leukaemia (T-ALL), in which the oncogenic Notch receptor 1 (NOTCH1) signalling drives MYC expression through a distal enhancer directly controlled by NOTCH1 transcriptional complexes and functions as a regulatory hub, recruiting a range of co-activators to sustain MYC transcription.^264^ Similarly, the expression of oncogenes such as TAL1 (T-cell acute lymphocytic leukaemia protein 1), ESR1 (Oestrogen receptor 1) and AR are also controlled by networks of super enhancers, thereby illustrating the significance of elucidating the mechanistic workings of these intricate protein-gene frameworks.^265–270^

In regard to the development of degraders targeting transcription factors binding to super enhancers, prominent advancements have been made specifically for the degradation of androgen receptor and oestrogen receptors, BCL6, STAT3, and IKZF1/IKZF3 with several PROTAC examples covered in this review.^271–281^ The success of developing and optimising PROTAC degraders targeting these key binders of super-enhancers provides a strong incentive to further elucidate the complex network defining oncogene overexpression and uncover more ‘PROTAC-able’ targets to create a more robust method of degrading oncogenic proteins. Recent computational frameworks have enabled the systematic mapping of super-enhancer (SE) regulatory networks, providing mechanistic insight into the pathways by which these enhancer clusters control oncogenic programs in cancer. These emerging bioinformatic approaches often integrate epigenomic datasets, comprised of Chromatin immunoprecipitation (ChIP) sequence data, gene annotations, motif enrichment, and defined autoregulatory loops, to identify SEs and construct the transcriptional regulatory circuits they support. This entails the explicit linking of super enhancers to transcription factors, along with upstream and downstream genes. The resulting network models describe the binding of TFs to SEs and in many cases reinforce each other through interconnected feedback loops. One of the first computational tools developed for the investigation of super enhancers is ROSE (rank ordering of super enhancers), introduced in 2013 by the Young lab at MIT.^259^ This framework defines enhancer regions from ChIP-sequence datasets enriched from enhancer associated chromatin marks or proteins, most commonly H3K27ac, BRD4 or Mediator.^282^ Adjacent enhancer peaks within a finite distance are ‘stitched’ together to represent enhancer clustering at loci. Such enhancer domains are then ranked by the cumulative signal intensity, generating a distribution that illustrates subsets of regions having disproportionately high occupancy as compared to typical enhancers. Since the release of ROSE, additional computational frameworks have provided greater insight into not only the identification of super enhancers, but also the transcription factors forming the regulatory network and their influence on upstream and downstream genetic elements. CRCMapper was developed by Saint-Andre et al. in 2016 for the inference of core regulatory circuitry (CRC), defined as an interconnected series of transcription factor autoregulatory loops anchored by SEs.^283^ The emergence of this framework has furthered the capabilities of identifying TFs associated with SEs, while also enabling the identification of interconnected networks of transcription factors. This network reconstruction is built through the integration of enhancer-gene assignment with transcription factor binding potential to generate a map of TF autoregulation. While ROSE and CRCMapper define and interpret super-enhancer landscapes from an enhancer-centric perspective, complementary computational frameworks infer regulatory control starting with transcriptional output, LISA (epigenetic landscape in silico detection analysis) enables systematic prediction of upstream transcription factors and regulators that drive a gene expression program, providing a method of inferring master regulators of SE-associated transcriptional states.^284^ Through combining inputted gene sets detailing upregulated genes and disease signatures, LISA leverages the integration of ChIP sequence binding and chromatin accessibility data to generate a list of plausible upstream TFs/chromatin regulators. Together, computational tools such as ROSE, CRCMapper and LISA each address a specific facet of protein-enhancer biology through a primary computational goal. By contrast, SEanalysis 2.0 functions as an integrative, network-level interrogation of SE function.^285^^,^^286^ This program is a web-based platform that enables the comprehensive analysis of regulatory networks formed by TFs, SEs and target genes, supported by an expanded SE atlas comprising 1,167,518 human SEs from 1,7349 samples and 550,226 mouse SEs from 931 samples thereby facilitating the comparison of enhancer-gene interactions between differing tissue types and disease states. In addition to enhanced modules for signalling pathway downstream analysis, upstream regulatory analysis and genomic region annotation, SEanalysis2.0 introduces dedicated tools for transcription factor regulatory analysis and sample comparison analysis, enabling the reconstruction of transcription factor-SE gene networks and identification of common versus pathology-specific SE circuits. Furthermore, overlaying GWAS (Genome-wide association study) risk variants onto SE maps provides a framework for connecting noncoding genetic associations to candidate regulatory elements, thereby strengthening mechanistic inference and prioritisation of SE-linked gene programs. Building on super enhancer centred network frameworks, a complementary class of computational networks has emerged that aims to predict epigenomic regulatory states using genomic sequence and integrative multi-omic inputs, thereby extending regulatory inference beyond enhancer circuity alone.^287–289^ In addition to enhancer activation profiles, core epigenetic mechanisms including DNA methylation, histone methylation and histone acetylation collectively shape chromatin accessibility and transcription factor occupancy, modulate enhancer competence, and stabilise oncogenic transcription factors across disease contexts.^290–295^ Accordingly, computational epigenomic prediction models that predict chromatin chemical modifications such as CpG methylation levels and histone mark deposition have become increasingly important for characterising regulatory heterogeneity in cancer and nominating epigenetic dependencies that may not be apparent from enhancer maps alone. Baiysa and Lombardi introduced DeepPTM, a deep learning framework for predicting histone post translational modifications (PTMs) using both transcription factor binding profiles and underlying DNA sequence features, thereby establishing histone chemical marks as a predictable regulatory outcome of transcription factor occupancy and genomic context. Using ENCODE (Encyclopedia of DNA elements) Tier 1 reference cell lines, which are extensively profiled benchmark cell types within the ENCODE consortium (H1, K562 and GM12878), the authors modelled promoter-associated PTMs, including H3K27me3 in H1 cells. Histone PTM labels were assigned based on the presence of ChIP sequence peaks in a 100 bp window around transcription start sites, while transcription factor binding features were represented by normalised ChIP sequence read counts near promoters, and sequence features were encoded using 6-mer frequency vectors.^296^ Notably, transcription factor binding data yielded more accurate histone mark prediction than DNA sequence alone, and the integration of both sequence and transcription factor binding improved performance. These results support that promoter histone PTM states are highly predictable from transcription factor occupancy patterns, consistent with the association between chromatin modification landscapes and TF binding. In parallel, recent epigenomic frameworks have increasingly investigated DNA methylation, a distinct but mechanistically coupled epigenetic regulatory layer that modulates chromatin accessibility and transcription levels.^297^^,^^298^ Feng et. al introduced AutoFE-Pointer, a lightweight deep learning framework for DNA methylation site prediction that addresses limitations of species-specific models by enabling cross-species generalisation within a single architecture.^299^ AutoFE-Pointer leverages an improved softened pointer network to dynamically extract and weigh information features from DNA sequences, integrating multiple feature extraction backbones such as CNN (Convolutional neural network), BiGRU (Bidirectional gated recurrent unit), and MLP (Multilayer perceptron) modules, whose outputs are scored and combined through pointer vector-based attention. Using benchmark datasets spanning three major methylation types, including 5hmc (5-hydroxymethylcytosine), 6 mA (N6-methyladenine), and 4mC (N4-methylcytosine) across 17 species, AutoFE-pointer achieved superior predictive performance relative to existing deep-learning methylation predictors. The model integrates both positional encoding and species-specific encoding to enhance representation learning across diverse taxa, providing a saleable strategy for epigenomic prediction in contexts where methylation data are heterogenous or limited across biological systems. Oncogenic transcriptional dysregulation is sustained by multilayered epigenetic regulation that varies across tumour types and patient subgroups, creating complex regulatory networks that cannot be resolved through single-assay profiling alone.^290^^,^^300^ Computational frameworks that reconstruct super enhancer regulatory architectures and predict chromatin chemical modifications, including DNA methylation and histone modification landscapes (histone methylation and acetylation), therefore play a central role in identifying disease drivers, stratifying clinically distinct populations, and identifying actionable protein targets for therapeutic intervention.^301–309^ The application of these computational approaches enables the mechanistic integration of enhancer circuitry with chromatin-state regulation, thereby facilitating the targeting of regulatory proteins and chromatin-associated factors responsible for sustaining oncogenic programs and may be amendable to PROTAC-mediated degradation.^310^^,^^311^

**Figure 9.**
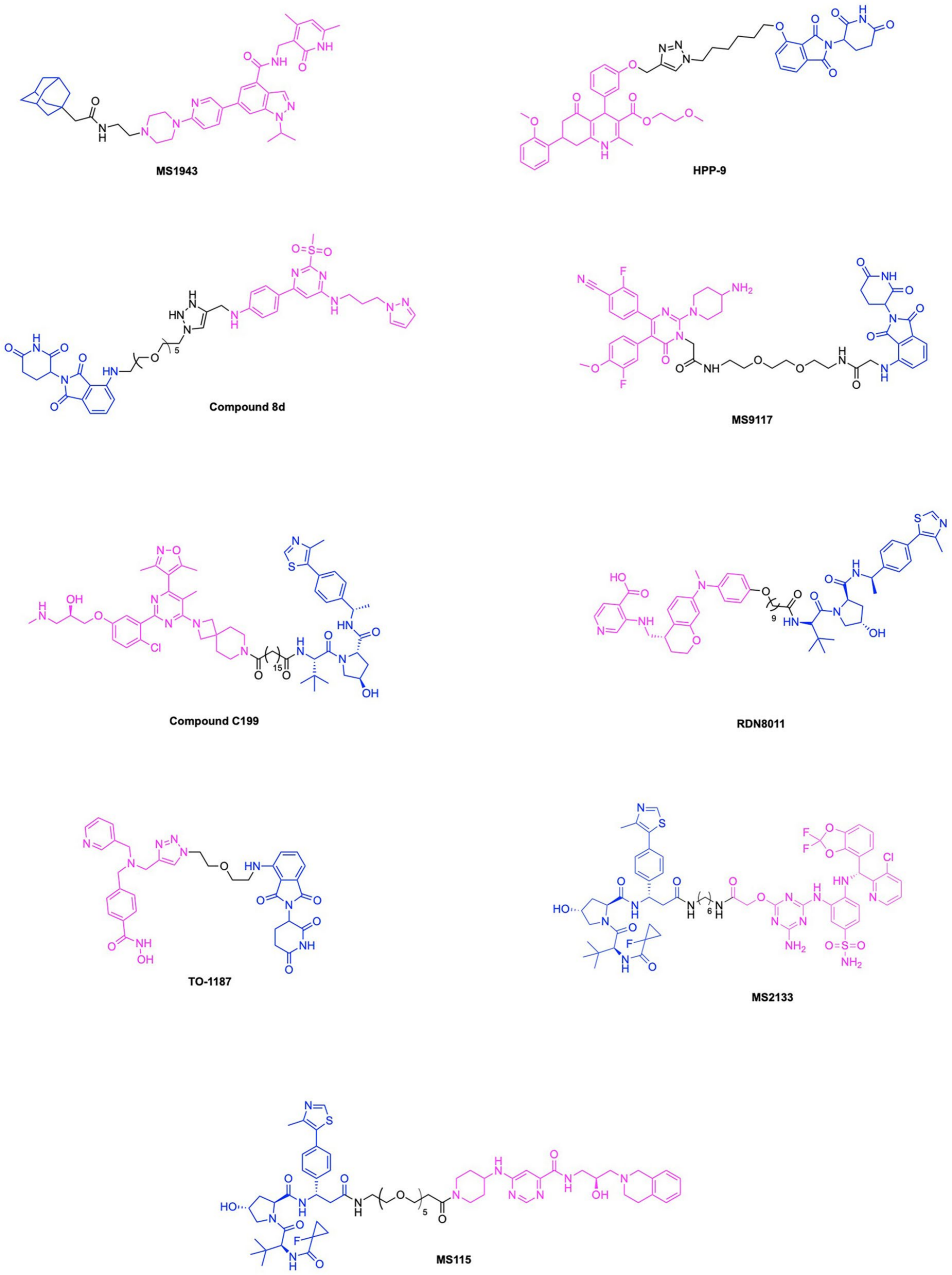
Overview of epigenetic regulator protein degraders covered in the subsequent section on the notable PROTACs within this field..^312–321^

The field of EZH2 degraders was spearheaded by the work of Ma et al. in the synthesis of MS1943 (Figure 9, Table 5).^312^ The unique molecular design of this degrader is comprised of an adamantyl group tethered to the EZH2 inhibitor C24 through a piperazine-based linker. The presence of an adamantyl group in the degrader plays a key role in inducing EZH2 degradation by mimicking the structural features of a misfolded protein, owing to its bulky and hydrophobic nature. This enables recognition by protein degradation machinery, thereby facilitating the elimination of EZH2 in the absence of E3 ligase recruiters. An IC_50_ of 120 nM was obtained, and a xenograft study in MDA-MB-468 tumours revealed tumour growth suppression with a dose of 150 mg/kg administered over 36 days. The success of this EZH2 degrader was complemented by subsequent innovations in PROTAC design, particularly those incorporating CRBN-recruiting and VHL-recruiting components.^322–326^ The degradation of the BET family of proteins has also become a valuable target of emerging PROTAC-based therapies. The dysregulation of this class of proteins involved in histone acetylation can lead to the activation of oncogenes and the occurrence of cancer.^310^ Recent advancements have been made in the development of successful BET inhibitors, but their applications towards reducing tumour cell proliferation are limited by their potential toxicity towards non-target proteins containing a bromodomain.^327^^,^^328^ The degradation of BET proteins through the ubiquitin proteasome system has thus been adopted to improve the dose associated limitations of existing BET inhibitors.

**Table 5.** Summary of Reported Epigenetic Regulator-Targeting PROTACs and Their In Vitro Potency Profiles.

PROTAC	POI	E3 Ligase/Binding Protein	Warhead	Linker Motif	Cell Line	In Vitro Potency/Affinity	References
MS1943	EZH2	Adamantane	EZH2-IN-14	Piperazine	MDA-MB-231, MDA-MB-468	IC_50_: 120 nM IC_50_ in MDA-MB-231: > 40 nM IC_50_ in MDA-MB-468: 3.41 µM	149
HPP-9	BET family	CRBN	HPI-1	Alkyl	SHH-GFP	pDC_50_ values: BRD2: 6.66 BRD3: 7.30 BRD4: 6.74	151
Compound 8d	BPTF	CRBN	TP238	PEG	Huh-7	DC_50_: 10.3 µM	152
MS9117	LSD-1	CRBN	CC-90011	PEG	THP-1 AML	EC_50_: 49 nM	153
Compound C199	PRMT4	VHL	EZM 2302	Alkyl	NCI-H929	DC_50_: 106 nM D_max_: 93.1%	154
RDN8011	KDM4	N/A	N/A	N/A	KYSE-150	DC_50_ for KDM4 family proteins: KDM4A: 37.53 nM KDM4B: 39.93 nM KDM4C: 49.41 KDM4D: >1000 nM IC_50_: 16 nM	155
TO-1187	HDAC6	CRBN	TO-317	PEG	MM.1S	DC_50_: 5.81 nM D_max_: 94%	156
MS2133	DOT1L	VHL	DOT1L ligand-1	Alkyl	THP-1	DC_50_ in THP-1 cells: 56 nM DC_50_ in MV4-11 cells: 25 nM	157
MS115	PRMT5, MEP50	VHL	EZP015666	PEG	MDA-MB-468	DC_50_ in PRMT5: 17.4 nM DC_50_ in MEP50: 11.3 nM IC_50_ in MDA-MB-468: 5.6 µM	158

DC_50_, half-maximal degradation concentration; D_max_, maximal fraction of a target protein that is degraded; IC_50_, half-maximal inhibitory concentration; EC_50_, half-maximal effective concentration; pDC_50_, negative log of the half-maximal degradation concentration.

**Figure 10.**
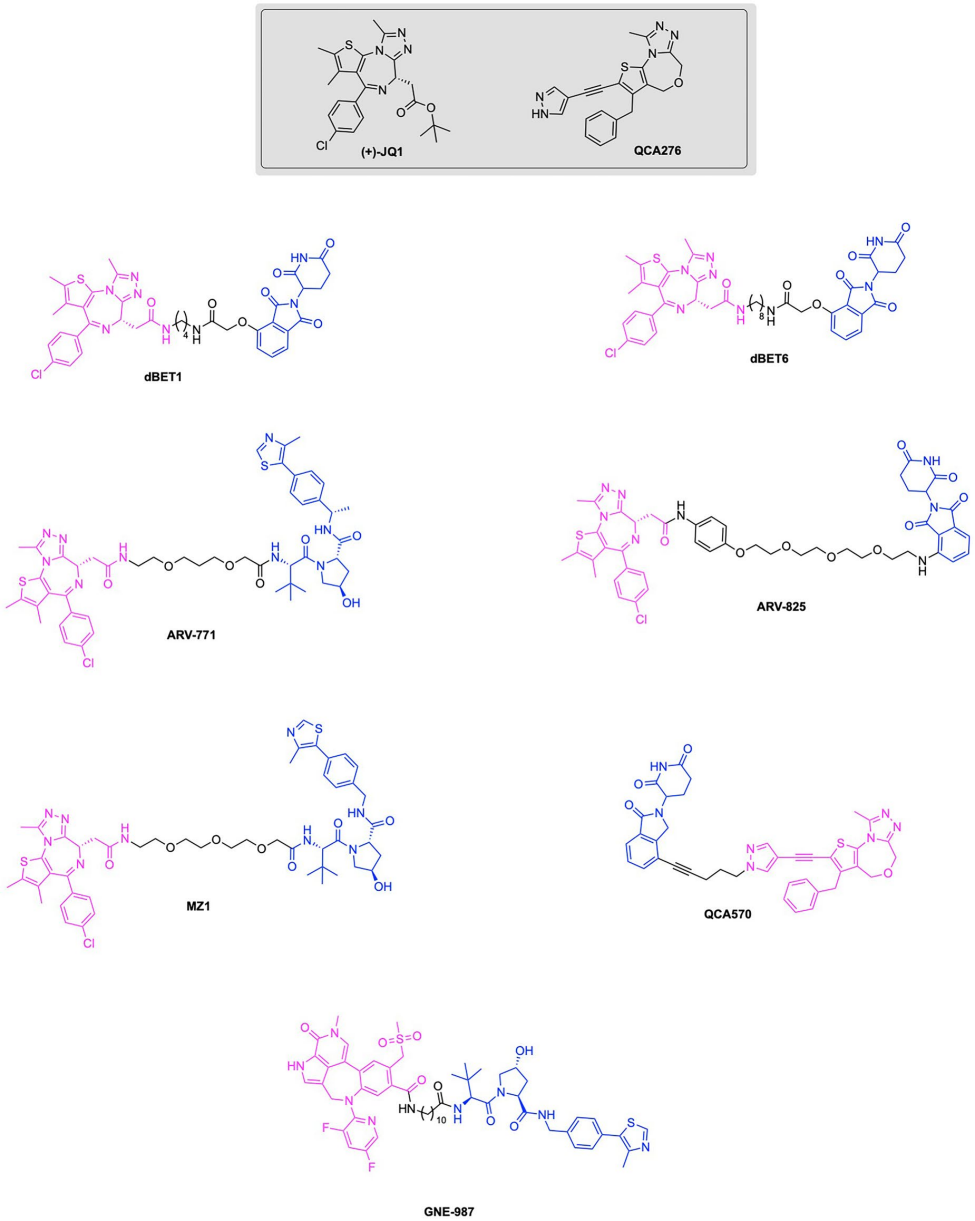
Overview of BET protein degraders covered in the subsequent section on the notable PROTACs within this field..^28^^,^^248^^,^^329–337^

The emergence of BET protein degraders was pioneered by the Crews group through the development of dBET1, a heterobifunctional molecule linking the BRD4 inhibitor (+)-JQ1 to a thalidomide ligand via an alkyl chain (Figure 10, Table 6).^28^ Compared with the constituent inhibitor, dBET1 demonstrated superior biological activity, inducing enhanced apoptosis in the acute myeloid leukaemia (AML) cell lines MV4-11 and in DHL4 lymphoma cells. The PROTAC dBET1 achieved an EC_50_ of 430 nM in the SUM149 cell line and exhibited improved antitumor effects as seen in *in vivo* studies utilising a human leukaemia xenograft. Selectivity of this degrader was exhibited in a quantitative proteomics study, in which the degradation of BRD2, BRD3 and BRD4 was demonstrated.^28^ Building on this success, the Crews group developed dBET6, a second-generation BET degrader featuring optimised linker composition and warhead positioning (Figure 10, Table 6).^329^ A significantly greater potency was exhibited by dBET6 in comparison to dBET1. This PROTAC was confirmed to act through the UPS, as demonstrated by competition experiments utilising JQ1, thalidomide, carfilzomib and MLN4924, which abolished the degradative effects of dBET6. Arvinas, a spin-off from the Crews group, played a pivotal role in advancing this rapidly emerging class of epigenetic protein degraders, particularly through the development of ARV-825 following the release of dBET1 (Figure 10, Table 6).^333^ The successful degradation of BRD4 was observed in the Burkitt’s lymphoma cell line, as seen by a DC_50_ value of less than 1 nM. Industry efforts have continued to broaden the scope of BET degraders. Genentech developed GNE-987, a VHL-recruiting PROTAC designed for AML therapy (Figure 10, Table 6).^338^ Although it remains at the pre-clinical stage, mechanistic studies revealed efficient ternary complex formation between GNE-987, the VHL E3 ligase, and the BRD4 bromodomain 1 at 7.8 µM, providing insights into the degradation mechanism of this PROTAC. Parallel efforts in academia have further diversified BET degrader design. Zengerle et al. reported the first VHL-recruiting BRD4 degrader MZ1, which tethers a JQ1 inhibitor to a VHL ligand through a flexible PEG3 linker (Figure 10, Table 6).^335^ MZ1 preferentially degraded BRD4, with treatment at 100 μM for 24 h inducing MYC downregulation and the upregulation of P21 and AREG, thus demonstrating transcriptional reprogramming driven by the degradation of BRD4. Qin et al. contributed to the rapidly expanding landscape of BET degraders with QCA570, a remarkably potent CRBN-recruiting degrader exhibiting effects at concentrations as low as 10 picomolar in the leukaemia cell lines MV4-11 (human biphenotypic B myelomonocytic leukaemia) and RS4;11 (human acute lymphoblastic leukaemia) within just 3 h (Figure 10, Table 6).^331^ The team not only discovered a novel BRD4 inhibitor but also utilised this molecular framework to engineer a highly efficacious BRD4 degrader. A structure-based design approach was taken to design the [1,4]oxazepine core scaffold, with the goal of emulating the key structural motif in the known BRD4 inhibitor JQ1. The developed inhibitor QCA276 was selected as the warhead and a series of linker optimizations in the designed analogs revealed that the most potent construct contains an ethynyl linker and lenalidomide CRBN recruiter. This highly potent PROTAC led to reduced c-Myc expression and apoptosis in the cell lines MV4-11, MOLM-13 and RS4;11. Numerous examples of successful PROTACs targeting the BET protein family have emerged since the primary example of dBET1 by the Crews group. Although we have provided an overview of solely the earliest and notable degraders of this class, the field of BET family degraders is undergoing an anticipated expansion of chemical space.

**Table 6.** Summary of Reported BET-Targeting PROTACs and Their In Vitro Potency Profiles.

PROTAC	POI	E3 Ligase/Binding Protein	Warhead	Linker Motif	Cell Line	In Vitro Potency/Affinity	References
dBET1	BRD4	CRBN	(+)-JQ1	Alkyl	MV4-11	IC_50_: 0.14 μM	24
dBET6	BRD4	CRBN	(+)-JQ1	Alkyl	MV4-11	IC_50_: 14 nM IC_50_ in MV-411: 10.33 nM	167
ARV-771	BRD4	VHL	(+)-JQ1	PEG	MOLM-13, MV4-11, RS4-11, Z-138	IC_50_ in MOLM-13: 7.45 nM IC_50_ in MV4-11: 0.43 nM IC_50_ in RS4-11: 2.4 nM IC_50_ in Z-138: 142 nM	168
ARV-825	BRD4	CRBN	(+)-JQ1	PEG	MOLM-13, MV4-11, RS4-11	IC_50_ in MOLM-13: 18.2 nM IC_50_ in MV4-11: 1.05 nM IC_50_ in RS4-11: 3.3 nM	172
MZ1	BRD4	VHL	(+)-JQ1	PEG	697, RS4-11	IC_50 _in 697: 0.117 μM IC_50_ in RS4-11: 0.199 μM	174
QCA570	BRD4	CRBN	QCA276	N/A	MOLM-13, MV4-11, RS4-11	IC_50_ in MOLM-13: 62 pM IC_50_ in MV4-11 : 8.3 pM IC_50_ in RS4-11: 32 pM	170
GNE-987	BRD4	VHL	PROTAC BRD4 ligand-1	Alkyl	EOL-1 AML, HL-60	DC_50_ in EOL- 1: AML: 0.03 nM IC_50_ in EOL-1: AML: 0.02 nM IC_50_ in HL-60: 0.03 nM	177

DC_50_, half-maximal degradation concentration; D_max_, maximal fraction of a target protein that is degraded; IC_50_, half-maximal inhibitory concentration; EC_50_, half-maximal effective concentration

#### Synthesis

**Scheme 12.**
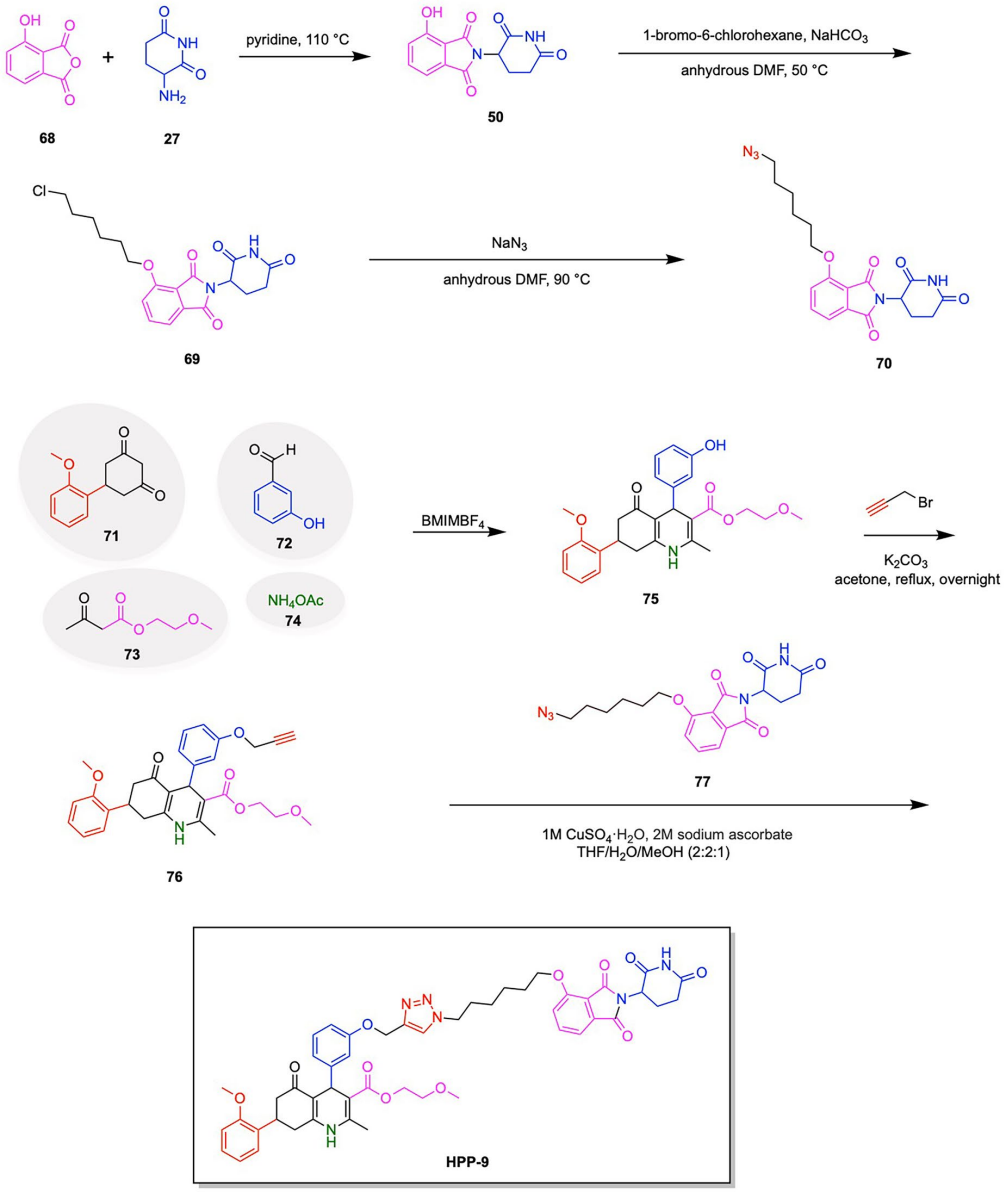
Synthesis Pathway for HPP-9 adapted from reference.^314^

Bagka et al. have recently implemented an innovative PROTAC based strategy to discover the target of Hedgehog Pathway inhibitor-1 (HPI-1) to be BET bromodomain proteins, demonstrating a novel use of PROTACs in target deconvolution and identifying key regulators of BET proteins.^314^ The hedgehog (Hh) pathway is a signalling cascade with roles in the onset of certain cancers, notably medulloblastoma and basal cell carcinoma.^328^ Current clinically approved drugs targeting the Hh pathway have limited effects on cancer activation originating from downstream pathways, providing an incentive to probe the protein targets of existing Hh inhibitors. A library of HPI-1 targeting PROTACs were synthesised with variations in linkers (polyethylene glycol, aliphatic chains), triazole, amide and ether attachments as well as E3 ligase ligands (hydroxythalidomide, pomalidomide, VHL peptide ligand).^314^ The potencies of the PROTACs HPP-1 through HPP-11 were assessed and the CRBN ligand in combination with short aliphatic linkers were determined to yield the highest potency. The PROTAC HPP-9 was determined to have the closest potency to the HPI-1 inhibitor for the degradation of the known downstream protein GLI-1 in both the cell lines SHH-LIGHT2 and SUFU-KO-LIGHT (Figure 9, Table 5).^314^ The degradation of the GLI-1 protein serves to confirm the efficacy of the designed HP1-1 based PROTACs for subsequent investigation of the downstream targets of HPI-1. Upon analysing the proteins affected by HPP-9, the significant degradation of two BET-family proteins, BRD3 and BRD4, were observed. Performed western blot assays confirmed the degradation of BRD2/3/4. The pDC_50_ of HPP-9 for BRD2, BRD3 and BRD4 in the mouse embryonic fibroblast NIH-3T3 cell line were determined to be 6.66, 7.30 and 6.74 respectively, demonstrating comparable values to the pDC_50_ of the known BET protein PROTAC dBET6.^314^ The synthesis of the PROTAC HPP-9 is shown in Scheme 12.^314^ The synthesis of the azide terminated thalidomide derivative was performed utilising a previously reported procedure by Zhou et al.^339^ It was reported in this work that the condensation of 3-hydroxyphthalic anhydride (compound **68)** with compound **27** was performed to yield 4-hydroxy-thalidomide (compound **50**). An alkylation of this obtained hydroxyl-based ligand using 1-bromo-6-chlorohexane yielded the thalidomide derivative compound **69**, which was then reacted with sodium azide to result in the formation of the azide-terminated thalidomide ligand compound **70**. Compound **71**, compound **72**, compound **73,** and compound **74** were reacted in the multicomponent Hantzsch reaction utilising 1-Butyl-3-methyl-imidazolium-tetrafluoroborate (BMIMBF_4_). The alkylation of the resulting compound **75** with propargyl bromide resulted in compound **76**. A copper-catalysed azide alkyne cycloaddition click chemistry reaction was used to react compound **76** with an azide-terminated thalidomide derivative compound **77** in sodium ascorbate and copper (II) sulphate to obtain the PROTAC variant HPP-9 (Scheme 12).

**Scheme 13.**
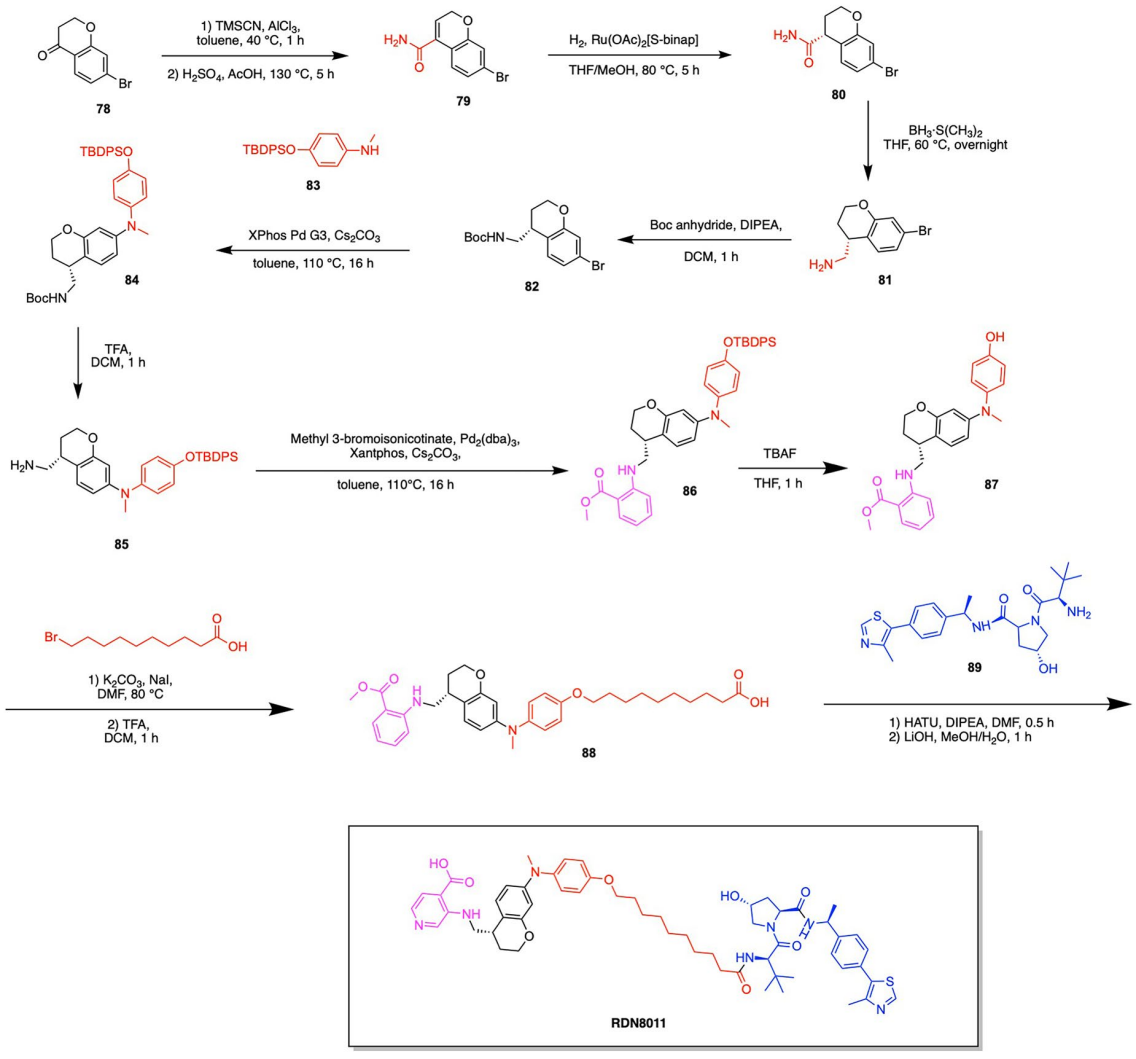
Synthesis pathway of RDN8011 adapted from reference.^318^

The discovery of the degrader compound 8d was achieved by Li et al. in efforts to enable the knockdown of the protein bromodomain PHD-finger containing transcription factor (BPTF), thereby ameliorating the immune system response to hepatocellular carcinoma (HCC) (Figure 9, Table 5).^315^ Natural killer cells are a type of liver lymphocyte that demonstrates significant potential for the treatment of HCC, specifically through mediating cytotoxicity and cytokine release that hinder the progression of this type of liver cancer. The synthesis of four degrader analogs was accomplished through incorporating the BPTF inhibitor TP238 with the CRBN ligand pomalidomide through utilising PEG linkers of varying lengths. Compound 8d, consisting of PEG5 linker, was found to be the most potent, with a DC_50_ value of 10.3 μM in the human hepatoma cell line Huh7.^315^ Compound 8d further exhibited superior selectivity for BPTF in comparison to the constituent inhibitor TP238, as seen by the lack of degradation of the proteins CECR2 and BRD9. The impact of 8d on facilitating an immune response to HCC was further investigated through RNA sequencing studies conducted on Huh7 cells. The results of this study revealed a reduction in the mRNA of HPSE, a gene encoding the enzyme heparanase, involved in cleaving glycans that serve as ligands on tumour cells.^315^^,^^340^ This suggests a potential link between BPTF degradation and modulation of tumour immunogenicity in HCC, thereby affirming the utility of BPTF degraders in furthering effective cancer therapies. Hosseini et al. synthesised the PROTAC MS9117 for the degradation of LSD1 (lysine specific demethylase 1), a key epigenetic regulator in acute myeloid leukaemia progression (Figure 9, Table 5).^316^ The known LSD1 inhibitor CC-90011 was strategically functionalised at the methyl group of the pyrimidine ring for covalent attachment to varying carbon and PEG linkers, along with a pomalidomide E3 ligase ligand. Amongst the 12 analogs synthesised, compound 11 (MS9117) emerged as the most promising degrader with an EC_50_ value of 49 nM in the THP-1 cell line.^316^ The PROTAC MS9117 consists of a PEG2 linker covalently bound to an acetamide moiety, using nitrogen as the atom connected to the CRBN binder. The effect of MS9117 in preventing the demethylation of histone H3 by LSD1 was determined through a western blot assay in which elevated levels of methylated histone H3 at lysine 4 (H3K4me1) were observed.^316^ Rao et al. achieved the degradation of lysine-specific demethylase 4 (KDM4), a histone lysine demethylase, through the development of the PROTAC RDN8011 (Figure 9, Table 5).^318^ The KDM4 family of enzymes serves to remove methyl groups from histone H3 lysine residues, in addition to serving non-enzymatic roles as a scaffolding protein in the translation initiation process. The overexpression of KDM4 has been linked to cancer progression and tumorigenesis for cancers such as prostate, lung, breast, oesophageal, and others.^341–346^ The synthesis of PROTAC analogs was accomplished through the covalent linkage of the inhibitor TACH101 to a VHL ligand through utilising linker variations. Amongst the synthesised compounds, RDN8011 emerged as the most potent analog, with a DC_50_ of 37.53 nM in the KYSE-150 oesophageal cell line.^318^ The inhibitor TACH101 was strategically functionalised through leveraging the solvent-exposed 4-isopropylbenzene moiety. A series of linker variations amongst the analogs indicated that a greater alkyl linker length yielded an improved degradation activity. The compound RDN8011 demonstrated selectivity in the degradation of KDM4, KDM4B and KDM4C, while sparing KDM4D.^318^ Additionally, proteasome inhibition experiments and ubiquitination assays in the presence of RDN8011 treatment revealed the dependence of KDM4 degradation on the ubiquitin proteasome system, along with the increased ubiquitination of KDM4. Furthermore, the treatment of KYSE-150 with RDN8011 resulted in increased levels of Histone H3 Lysine 36 (H3K36me3) methylation, with a 1000 nM concentration of this PROTAC resulting in a two-fold increase in H3K36me3 levels in comparison to a DMSO control.^318^ The synthetic route of RDN8011 is outlined in Scheme 13 and proceeds as follows.^318^ The compound bromochroman-4-one (compound **78**) underwent a cyanation and acidic hydrolysis to afford the amide compound **79**. An asymmetric reduction was performed using the ruthenium pre-catalyst (*S*)-Ru(OAc)_2_ (BINAP) to yield the R-enantiomer compound **80**. A borane reduction was performed using this compound to yield the primary amine in compound **81**. Following the Boc protection of the primary amine, the resulting Compound **82** was reacted in a Buchwald-Hartwig coupling with the amine of compound **83** in the presence of XPhos to afford compound **84**. A second Buchwald-Hartwig coupling was performed following the deprotection of compound **84** to afford **86**, which underwent a deprotection with tetrabutylammonium fluoride (TBAF) to afford compound **87**. This compound was reacted in a Williamson ether reaction with an alkyl bromide to afford compound **88**, which was subsequently reacted with the VHL ligand compound **89** in an amide coupling reaction to afford the PROTAC RDN8011.

Histone deacetylases (HDACs) are a category of epigenetic regulator enzymes with key roles in the removal of acetyl groups from histones, serving to tighten chromatin compactness and reduce transcriptional levels. The effects of HDACs are multifaceted, as seen through their roles in the modification of non-histone proteins in the cytosol, thereby affecting cellular structure homeostasis.^347^^,^^348^ Alterations to HDAC functionality play a significant role in tumour differentiation and the proliferation of cancer cells, due to the associated reduction of apoptosis signals related to changes in gene control.^349^^,^^350^ The TPD of HDACs has thus emerged as a promising strategy for the treatment of tumour cells, and numerous successful degraders have been discovered.^311^

**Figure 11.**
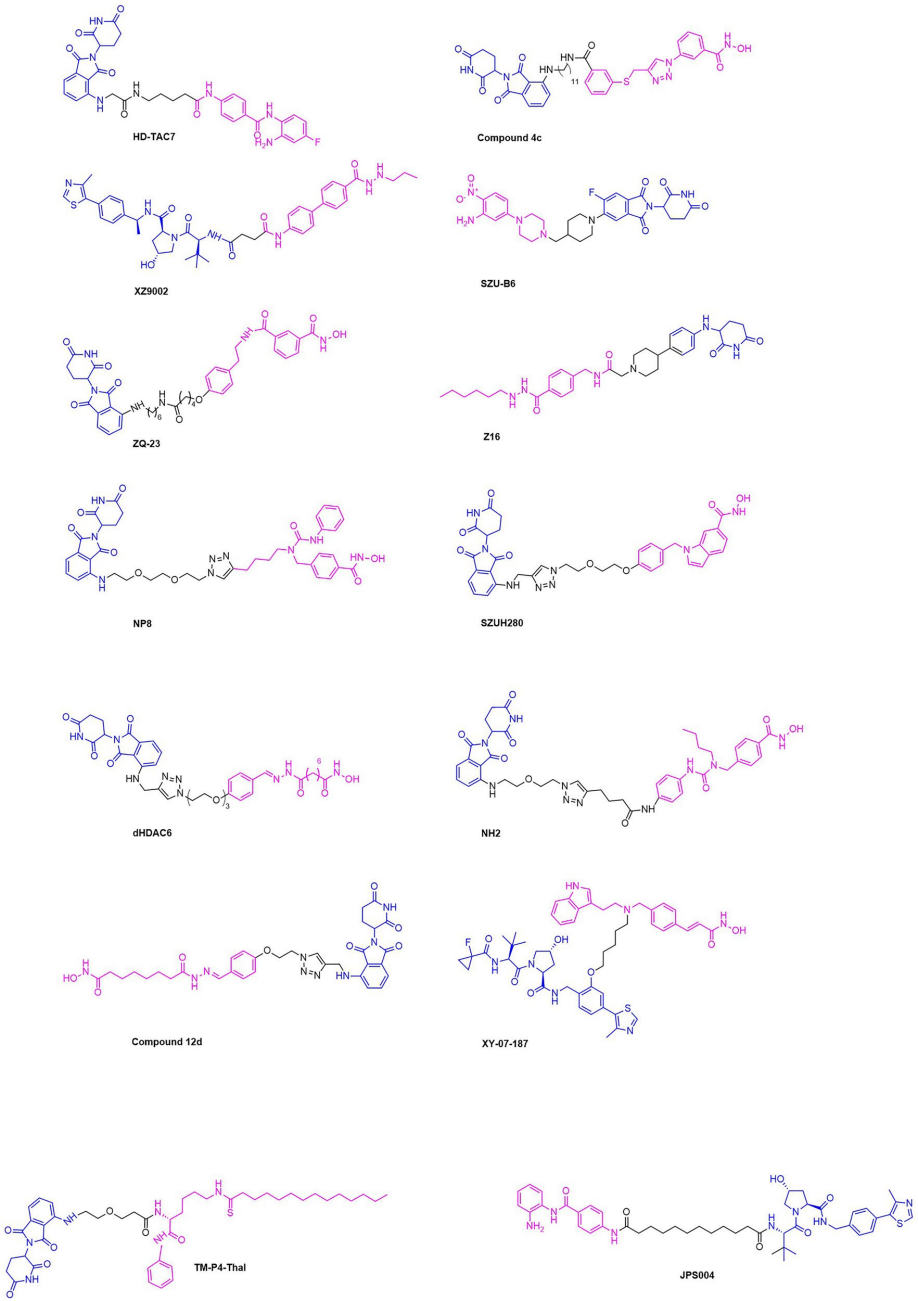
Significant progress has been made in expanding the PROTAC strategy to Class I HDACs..^351–365^

**Scheme 14.**
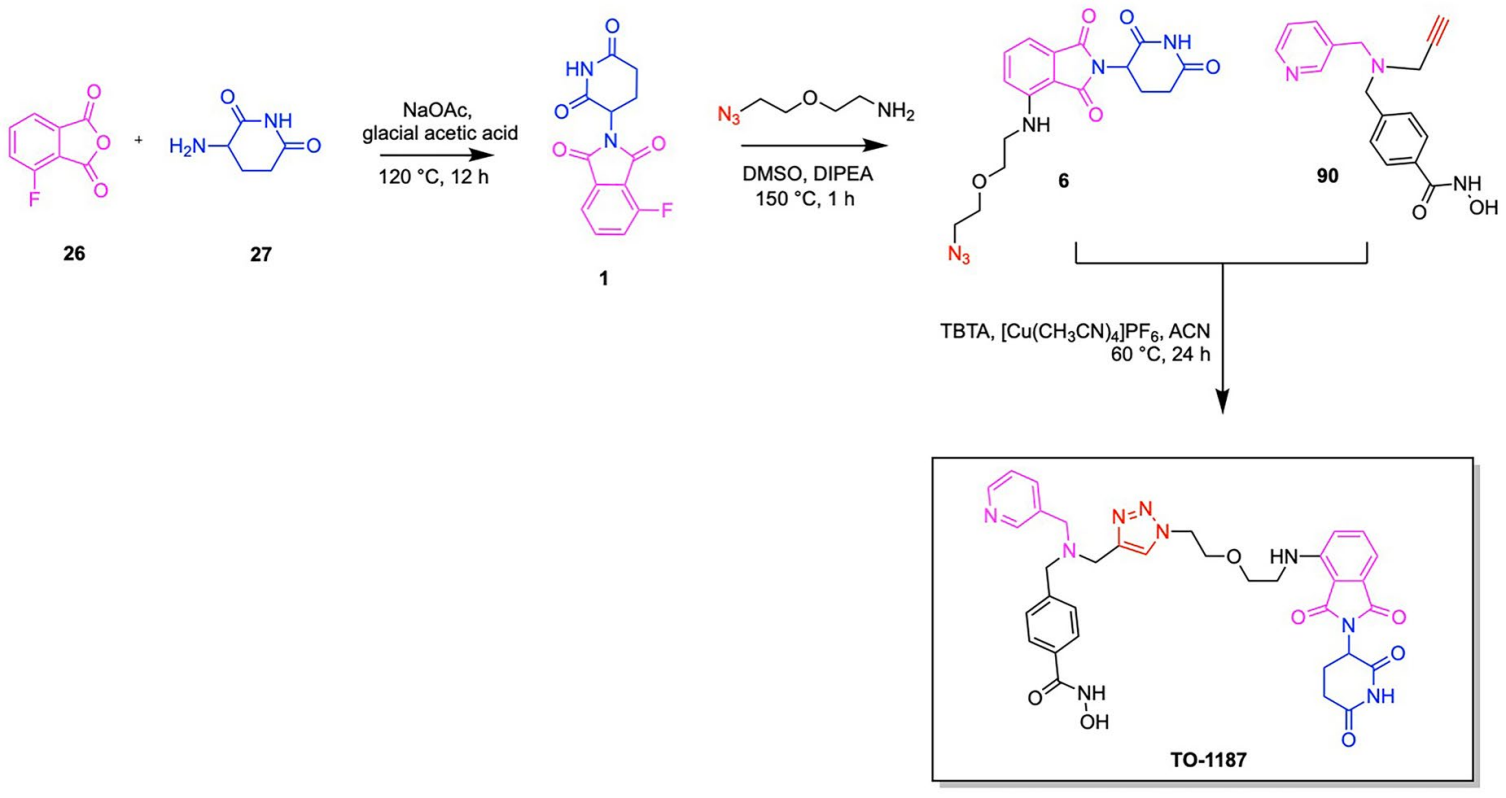
Synthesis Pathway of TO-1187 adapted from reference.^319^

In the field of HDAC3 degraders, Cao et al. reported the development of HD-TAC7 and Xiao et al. introduced XZ9002, both exhibiting selective and potent degradation of HDAC3 with DC_50_ values of 0.32 μM and 42 nM respectively (Figure 11, Table 7).^351^^,^^353^ The work of Smalley et al. has also been contributory in advancing Class I HDAC degraders with selectivity of HDAC1-3. Their most potent degrader, JPS004, comprises a benzamide-based HDAC inhibitor conjugated to a VHL E3 ligase ligand via an alkyl linker (Figure 11, Table 7).^365^ Although the successful degradation of HDAC1/2/3 was noted in colon cancer cells, Smalley et al. conducted a follow up study with the aim of optimising the heterobifunctional degrader for enhanced potency. Alkyl and PEG linkers were identified as optimal candidates for molecular design. Furthermore, the degradation of HDAC1/2/3 was found to play a critical role in inducing cell cycle arrest, apoptosis and transcriptional changes.^364^ Further innovation in PROTAC design emerged from the incorporation of a cIAP E3 ligase ligand, resulting in the development of the degraders JPS026 and JPS027, which demonstrated enhanced efficacy in HCT116 cells.^366^ Parallel advancements have been made in the field of HDAC8-targeting degraders. Chotitumvanee et al. introduced the CRBN-recruiting PROTAC Compound 4c, Sun et al. reported ZQ-23, Huang et al. developed SZUH280 and Zhao et al. designed the hydrazide-based degrader Z16, serving to broaden the chemical space for HDAC8 PROTAC design (Figure 11, Table 7).^352^^,^^355^^,^^356^^,^^358^ The scope of HDAC6-targeting PROTACs has also grown significantly. Yang et al. pioneered the field with dHDAC6, exhibiting a DC_50_ of 34 nM in MCF-7 cells (Figure 11, Table 7).^359^ This group has also made significant contributions to the optimisation of CRBN ligands for HDAC6 PROTACs, facilitating the discovery of additional degraders such as NP8, compound 12d and NH2 (Figure 11, Table 7).^357^^,^^361^^,^^367^ Degradation efforts based on the PROTAC design have also been extended to Class III of HDACs, constituting Sirtuin proteins. Schiedel et al. accomplished the degradation of Sirtuin 2 (Sirt2), a member of class III of HDACs characterised by their dependence on NAD+ for their deacetylase activity (Figure 11). Through the linkage of a type of Sirt2 inhibitors known as SirReals to a thalidomide CRBN ligand through an N-butyl-2-oxyacetamide linker, an IC_50_ value of 0.25 μM was achieved in the HeLa cell line.^368^ Subsequent advancement in the degradation of Sirt2 was also accomplished by Hong et al. in their degrader TM-P4-Thal, having an IC_50_ value as low as 0.078 μM in a performed *in vitro* enzyme assay (Figure 11, Table 7).^363^ Huang et al. have expanded the scope of Sirtuin proteins degradation through their development of the Sirt-6 targeting PROTAC SZU-B6, having a DC_50_ of around 45.3 nM in the SK-HEP-1 cell line (Figure 11, Table 7).^354^ Efforts to develop multi-targeting pan-HDAC degraders have also yielded promising results. One such example is the degrader XY-07187, which has demonstrated the simultaneous degradation of HDAC3, HDAC6 and HDAC8, highlighting the potential for targeting multiple proteins within the HDAC family (Figure 11, Table 7).^362^ The current landscape of HDAC degraders underscores the promise of PROTAC technology in further developing more potent and diverse degraders for a number of POIs. Building on this foundation, we herein describe a recent example of HDAC degraders detailing the selective targeting of HDAC6. Garcha et al. achieved the selective degradation of HDAC6 in cellular and *in vivo* through the development of a potent PROTAC TO-1187 (Figure 9, Table 5).^319^ This developed PROTAC did not affect the concentration of prevalent CRBN neo substrates, specifically IKZF1, IKZF3, CK1α, SALL4 and GSPT1. A previously reported HDAC6 inhibitor created by this group, known as TO-317, was conjugated to the CRBN ligand pomalidomide utilising a diverse range of linkers: alkyl-triazole-alkyl, alkyl-triazole-PEG, or the direct instalment of an alkyl or PEG linker.^319^ An analysis of *in vitro* inhibitory activity revealed that PROTAC 9 containing a PEG2 linker achieved the lowest DC_50_ value of 5.01 nM. The PROTAC 8 (methyl-triazole-PEG1 linker) and the PROTAC 3 (methyl-triazole-propyl) along with PROTAC 9 exhibited the hook effect at a range of 5 nM to 10 nM, indicating the reduction in ternary complex formation and reduced degradation efficiency.^319^ Despite the superior DC_50_ value of PROTAC 9, PROTAC 8 (TO-1187) was discovered to be the optimal choice due to having a PAMPA cell permeability of 5.82 (-log Pe), indicating ideal cell permeability. The PROTAC TO-1187 also demonstrated a half-life of 80 min in the performed whole blood stability assay, indicating its superior potential as an HDAC6 degrader.^319^ The selective degradation of HDAC6 was observed by TO-1187 in the global proteomics evaluation performed. Data from proteomics and quantification of DC_50_ in cells was obtained from the human multiple myeloma MM.1S cell line. The synthesis of the PROTAC TO-1187 is shown in Scheme 14.^319^ The first step of the synthesis utilises a condensation reaction between 3-fluorophthalic anhydride (compound **26**) and 3-amino-2,6-piperidinedione (compound **27**) to afford 4-fluoro thalidomide (compound **1**). The product is then reacted with azido-PEG1-amine in a nucleophilic aromatic substitution reaction to yield compound **6.** Compound **6** was reacted with compound **90** (an alkyne functionalised HDAC6 ligand) in a copper-catalysed azide alkyne cycloaddition click chemistry reaction in the presence of TBTA (Tris[(1-benzyl-1*H*-1,2,3-triazol-4-yl)methyl]amine), [Cu(CH_3_CN)_4_]PF_6_ (Tetrakis(acetonitrile)copper(I) hexafluorophosphate) and acetonitrile to afford the PROTAC TO-1187 (Scheme 14).

**Table 7.** Summary of Reported HDAC-Targeting PROTACs and Their In Vitro Potency Profiles.

PROTAC	POI	E3 Ligase/Binding Protein	Warhead	Linker Motif	Cell Line	In Vitro Potency/Affinity	References
HD-TAC7	HDAC 1/2/3	CRBN	CI994	Alkyl	RAW 264.7 macrophage cell line	IC_50_ HDAC1: 3.6 µM IC_50_ HDAC2: 4.2 µM IC_50_ HDAC3: 1.1 µM DC_50_ in RAW 264.7: 0.32 µM	191
Compound 4c	HDAC8	CRBN	NCC-149	Alkyl	Jurkat cells	IC_50_: 0.372 µM DC_50_ in Jurkat cells: 0.702 µM	192
XZ9002	HDAC3	VHL	SR-3558	Alkyl	MDA-MB-468	DC_50_: 42 µM	193
SZU-B6	SIRT6	CRBN	5-(4-methylpiperazin-1-yl)-2-nitroaniline	Piperidine	SK-HEP-1, Huh-7	DC_50_ in SK-HEP-1: 45 nM DC_50_ in Huh-7: 154 nM IC_50_ in SK-HEP-1: 1.51 µM	194
ZQ-23	HDAC8	CRBN	BRD73954	Alkyl/Amide	HCT-116	DC_50_: 147 nM D_max_: 93%	195
Z-16	HDAC8	CRBN	N/A	Alkyl	Jurkat, HCT-116	DC_50_ in Jurkat cells: 0.32 nM DC_50_ in HCT-116: 2.4 nM IC_50_ in Jurkat cells: 1.7 µM IC_50_ in HCT-116: 1.4 µM	196
NP8	HDAC6	CRBN	Nexturastat A	PEG	MM.1S	DC_50_: 3.8 nM	197
SZUH280	HDAC8	CRBN	PCI-34051	PEG	A549	DC_50_: 0.58 µM	198
dHDAC6	HDAC6	CRBN	WT-161	PEG	MCF-7	DC_50_: 34 nM	199
NH2	HDAC6	CRBN	Nexturastat A	Alkyl/PEG	MM.1S	DC_50_: 3.2 nM	200
Compound 12d	HDAC6	CRBN	Nexturastat A	PEG	MM.1S	IC_50_: 8.7 nM DC_50_: 1.64 nM	201
XY-07-187	HDAC3/6/8	VHL	Dacinostat	Alkyl	MM.1S, KELLY	MM.1S: ∼ −2.80 log_2_FC for HDAC3 ∼ −2.80 log_2_FC for HDAC6 ∼ −2.80 log_2_FC for HDAC8 KELLY: ∼ −1.4 log_2_FC for HDAC3 ∼ −1.4 log_2_FC for HDAC6 ∼ −1.4 log_2_FC for HDAC8	202
TM-P4-Thal	SIRT2	CRBN	Thiomyristoyl	PEG	MCF-7, BT-549	IC_50_: 0.078 µM (SIRT2-selective degradation induced in MCF-7 and BT-549 cells at 0.5-10 μM)	203
JPS004	HDAC1/2/3	VHL	CI994	Alkyl	N/A	IC_50 _: 16.8 μM for LSD1-COREST-HDAC1 complex	204, 205

DC_50_, half-maximal degradation concentration; D_max_, maximal fraction of a target protein that is degraded; IC_50_, half-maximal inhibitory concentration; EC_50_, half-maximal effective concentration

**Scheme 15.**
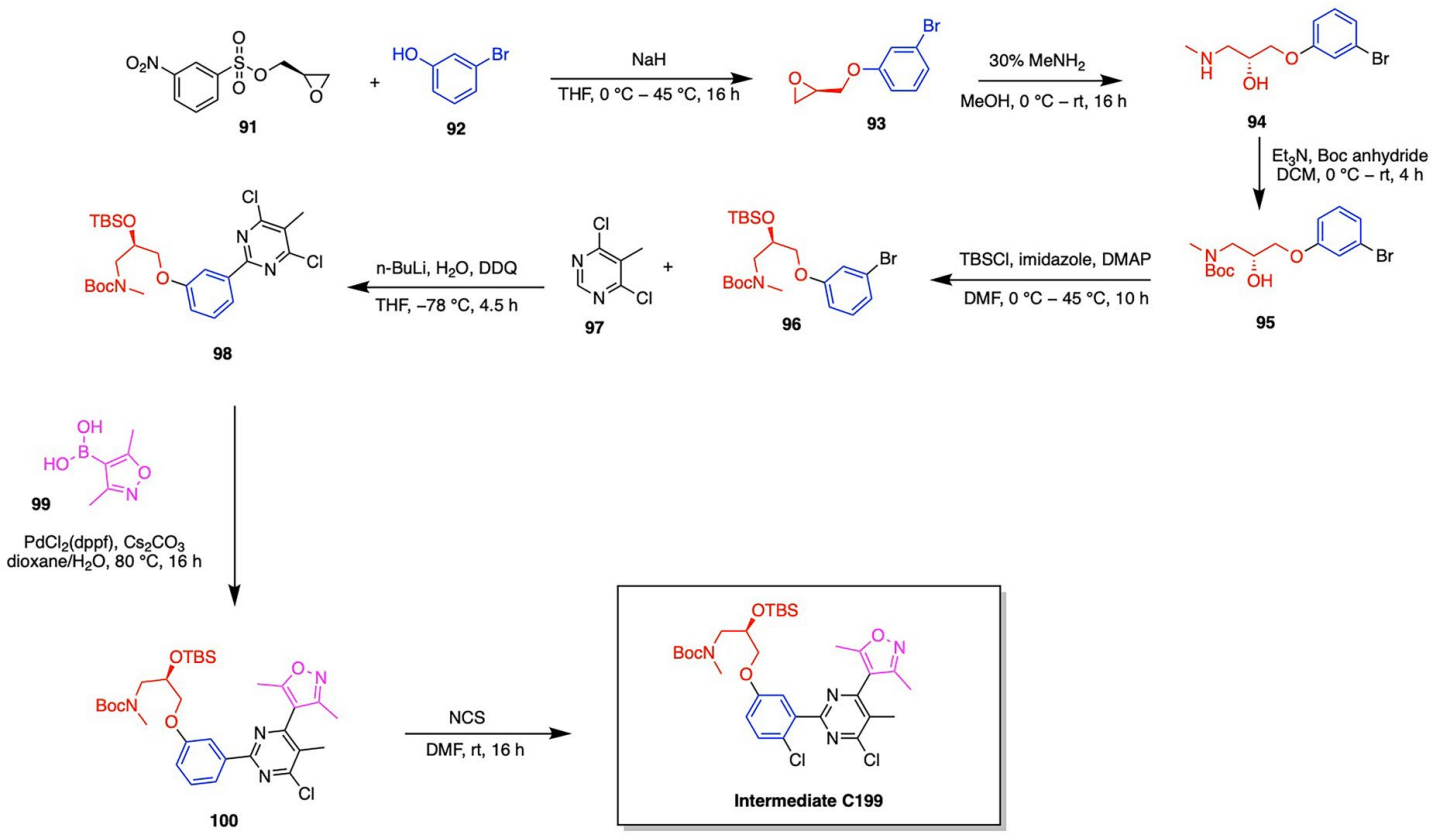
Synthesis pathway of an intermediate of Compound C199 adapted from reference.^317^

**Scheme 16.**
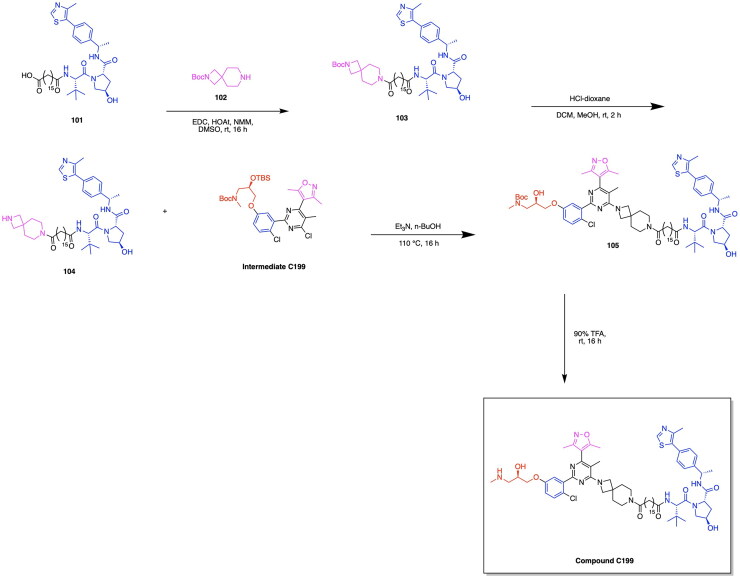
Synthesis pathway of Compound C199 adapted from reference.^367^

The PROTAC-mediated degradation of epigenetic regulators has been expanded to include a broader range of pathogenic proteins beyond the HDAC and BET protein families. Recently, Yim et al. developed a novel PROTAC degrader known as MS2133 to target DOT1L, a methyltransferase enzyme involved in the methylation of histone H3 lysine 79 and having key roles in leukaemia progression (Figure 9, Table 5).^320^ A series of compounds incorporating a previously identified DOT1L degrader, known as Compound 2, were covalently attached to an alkyl linker of varying lengths and one of two different types of VHL ligands.^320^^,^^369^ The modification of the DOT1L inhibitor was performed through utilising the solvent-exposed methoxy moiety present on the molecule. They found that compound 13 had high potency in causing the degradation of DOT1L in a time and concentration dependent manner, with high selectivity of the DOT1L methyltransferase and minimal effect of DOT1L mRNA expression.^320^ This compound achieved a DC_50_ value of 56 nM in the human leukaemia monocytic THP-1 cell line and 15 nM in the MV4-11 cell line.^320^ Zhong et al. reported a PROTAC degrader for protein arginine methyltransferase 5 (PRMT5), an epigenetic regulator having roles in the symmetric demethylation of arginine residues as well as common overexpression in cancers such as glioblastoma, prostate cancer, breast cancer, leukaemia, and colorectal cancer.^320^^,^^370^^,^^371^ A structure-activity relationship study was conducted to explore various linkers and ligands for the target protein PRMT5 along with the E3 ligase VHL. A highly selective and potent degrader known as MS115 was developed, demonstrating the degradation of both PRMT5 and the coactivator MEP50 in a concentration and time dependent manner and through the usage of the ubiquitin proteasome system (Figure 9, Table 5).^320^ The structure of the MS115 PROTAC incorporates the previously developed PRMT5 inhibitor GSK3326595 to a VHL ligand using a PEG5 linker. The obtained DC_50_ for MS115 is 17.4 nM for PRMT5 and 11.3 nM for the protein MEP50 in the MDA-MB-468 cell line (triple negative breast cancer cell line).^320^ Recent progress has been made in the degradation of the protein arginine methyltransferase family, namely PRMT4. Ju et al. have achieved the synthesis of the PRMT4 degrader C199, having a DC_50_ value of 106 nM (Figure 9, Table 5).^317^ The target enzyme PRMT4 has been identified previously as a key negative regulator of tumour immunity and inhibition of this protein holds benefits in enhancing T cell activity and increases tumour sensitivity to cytotoxic T cells through the upregulation of type I interferon response. The design of PRMT4 degraders was accomplished through the covalent attachment of the potent inhibitor EZM2302 to either a thalidomide or a VHL ligand through utilising PEG and alkyl linkers of varying lengths. The compound C199 was identified as the most potent, yielding a maximum degradation of 93.1% in HCI-H929 cells.^317^ This compound consisted of a 15-carbon linker connecting the EZM2304 POI ligand to a VHL ligand. A series of competition experiments targeting the proteasome, VHL E3 ligase and lysosomal degradation pathways demonstrated that PRMT4 levels were restored upon disruption of these components. These results indicate that the PRMT4 degrader C199 is a PROTAC that leverages the ubiquitin proteasome system to enable the degradation of the POI. The methylation of the PRMT4 inhibitors polyadenylate-binding protein 1 (PABP1) and BRG-associated factor 155 (BAF155) was assessed and a concentration of 0.25 nM yielded a 97% inhibition of PABP1 and a 71% inhibition of BAF155, thereby demonstrating superior efficacy in comparison to the inhibitor EZM2302.^317^ The selectivity of C199 was confirmed through a global proteomics analysis, in which seven proteins were downregulated amongst 4838 proteins. The synthesis pathway for compound C199 and a key intermediate in the synthetic route are depicted in Scheme 15 and Scheme 16 respectively. The synthesis of the intermediate C199 is described as follows. Glycidyl 3-nitrobenzenesulfonate (compound **91**) is reacted in a nucleophilic substitution reaction with 3-bromophenol (compound **92**) to afford compound **93**. A subsequent epoxide ring opening is performed, followed by the protection of a secondary amine and alcohol through utilising Boc anhydride and tert-butyldimethylsilyl chloride. A nucleophilic aromatic substitution reaction is performed with compound **96** in the presence of n-Butyllithium and 2,3-Dichloro-5.6-dicyano-1,4-benzoquinone (DDQ) to afford compound **98**. This compound is reacted with the boronic acid compound **99** in a Suzuki coupling to afford compound **100**, which is subsequently chlorinated to afford the intermediate C199. The derivatized VHL ligand compound **101** was reacted with 2,7-diazaspiro[3.5]nonane-2-carboxylate (compound **102**) in an amide coupling in the presence of EDC, HOAt and NMM to afford compound **103**. The secondary amine of this compound was deprotected with HCl, prior to reacting with the intermediate C199 in a nucleophilic aromatic substitution reaction to afford compound **105**. This compound was deprotected in the presence of TFA to yield the PROTAC compound C199.

### Adjacent targeted protein degradation strategies

Since the rapid diversification of PROTAC design, the heterobifunctional molecule concept has been adapted to harness diverse cellular pathways for achieving targeted biological effects beyond UPS-mediated protein degradation. Similar to the proteasomal degradation of the POI induced by PROTACs, emerging molecular designs aim to facilitate degradation via other prevalent elimination mechanisms in the cell, namely the lysosomal pathway, autophagy pathway. This has resulted in the emergence of molecules such as LYTACs and AUTACs. Pioneered by the Bertozzi group at Stanford, LYTACS (lysosome targeting chimaeras) enable the degradation of extracellular proteins through the incorporation of a ligand for a lysosome targeting receptor,^372^ thereby enabling lysosomal degradation via receptor-mediated endocytosis. Likewise, AUTACS (Autophagy targeting chimaeras) are heterobifunctional molecules targeting intracellular proteins with a degradation signal mimicking s-guanylation, a post-translational modification that induces selective autophagy.^373^ This process serves to recruit LC3-associated machinery to selectively degrade the modified targets through the autophagy-lysosome pathway. AbTACs (Antibody-based PROTACs) are bispecific antibodies that simultaneously bind a cell-surface target protein and an E3 ubiquitin ligase, thereby promoting ubiquitination and lysosomal degradation of membrane-bound proteins. These three heterobifunctional designs extend protein degradation to novel reaches that were initially inaccessible by the standard PROTAC model. The utility of the heterobifunctional design also has applications towards the precise modulation of protein function, thereby affecting the activity or signalling role of key components in biological pathways. Molecules such as PhosTACs and DEPTACs facilitate the targeted phosphorylation or dephosphorylation of the POI respectively, thereby utilising post-translational modifications to reprogram signalling networks governing cellular processes.

## Outlook

The rapid growth of PROTAC technology through the expansion of scope and improved molecular design has redefined the approach to targeting disease-related proteins, notably targets considered to be undruggable by small-molecule inhibitors. The examples of PARP, GPX4 and epigenetic regulators exemplify how rational degrader design integrating linker and ligand variations with rigorous validation of specificity and potency enables TPD to be extended towards protein classes governing key processes in cellular homeostasis, including DNA repair, chromatin modelling and changes to redox balance, thereby promoting outcomes such as ferroptotic cell death. As the frontier of PROTAC development continues to advance towards expanding the degradable proteome, several challenges persist, particularly those related to expanding target diversity, improving selectivity and oral bioavailability, optimising multivalent binding and addressing mechanisms of resistance. With novel disease-associated proteins being discovered at an increasing rate, extending the TPD approach to these emerging targets remains challenging. Key obstacles include the identification of POI ligands for PROTAC molecular design and the optimisation of E3 ligase ligand and linker pairing required to achieve a stable ternary complex. Historically, the design of PROTACs has largely relied on repurposing existing small molecule inhibitors as ligands for the protein of interest, thus utilising a known protein-ligand interaction to facilitate the UPS-mediated degradation. Although this strategy provides a pragmatic entry point into expanding the frontier of PROTACs, the lack of small-molecule inhibitors available for certain disease-associated proteins remains a limitation for expanding the reaches of the targeted protein degradation model.^38^^,^^374^^,^^375^ Various strategies have been developed to incorporate new methods for introducing affinity towards the POI, including the use of diverse warheads such as peptide-based warheads, antibody warheads and nucleotide-based warheads.^375–378^ However, such methods present the necessity for a trade-off of cell permeability due to the inherently larger molecular weight associated with such biological macromolecules. Parallel to advances in POI targeting, there remains a necessity for expanding the repertoire of E3 ligases recruited by PROTACs. In spite of the conceptual flexibility of PROTAC design, the narrow range of E3 ligases currently utilised remains a central limitation to target diversification. Only a fraction of the biologically available E3 ligases are employed in current TPD approaches, specifically VHL and CRBN ligands. The presence of such a limitation arises from challenges in ligandability; most E3 ligases lack well-known and high affinity ligands that can be incorporated into the PROTAC model.^379^ This limitation is furthered by the lack of structural and biophysical data available for the diversity of E3 ligases present, proving the rational design of ligands to be challenging.^124^ The incorporation of a greater range of E3 ligase proteins holds immense value in the enablement of protein degradation, as the expression of several E3 ligase proteins is tissue-specific. This represents an additional constraint on the broad applicability of the PROTAC approach to TPD. Another critical aspect of PROTAC design that continues to impede the advancement of this field is the optimisation of oral bioavailability. Since the emergence of Lipinski’s rule of five in 1997, this guideline has remained a key governing factor for the determination of oral bioavailability of a small molecule. The realm of heterobifunctional molecules is especially subject to violations of this rule due to the inherently large molecular weight associated with the multi component structure of PROTACs and similar TPD tools. Despite recent advancements exemplified by the development of orally bioavailable PROTACs such as Arvinas’ ARV-110 and ARV-471, the chemical space compatible with both potent and selective degradation and compliance with physiochemical parameters for oral bioavailability remains narrow.^380^ Addressing these competing demands continues to represent a major design challenge in this field. An investigation into the oral bioavailability of ARV-110 in the work of He at al. revealed that oral bioavailability in rats was only 10.75% under fasted conditions and 20.97% when fed, highlighting that even clinically active PROTACs continue to face challenges in oral availability.^381^ The comprehensive analysis of Hornberger et al. on PROTAC physiochemical property determinants revealed the key factors contributing to the limited subset of PROTACs that can be orally administered. The analysis of an extensive Arvinas database of over 1800 PROTACs indicated that large molecular weight, polarity and high hydrogen bonding properties remain among the most restrictive parameters towards PROTAC oral bioavailability. The established quantitative framework delineated a refined and more lenient set of restrictions for PROTAC bioavailability, constituting a requirement 950 Da or less, TPSA of less than 200 angstroms squared and a cLogP in the range of 1–7, demonstrating an expansion beyond the Lipinski rule of five but nonetheless posing restraints on PROTAC molecular design.^382^ Recent advancements in protein degradation tools have shown promise in enabling a broader range of proteins to be targeted with molecules that better comply with Lipinski’s rule of five and other key criteria governing oral bioavailability. The emergence of molecular glue degraders has further advanced this field by enabling smaller, more permeable molecules that better satisfy the physicochemical requirements for oral bioavailability. The architecture of molecular glues does not require a linker or distinct binding domains, as target engagement is achieved through a single small-molecule scaffold. Despite recent advances in PROTAC design aimed at enhancing binding to both the POI and E3 ligase, expanding the range of targetable proteins, and ameliorating oral bioavailability, the selectivity and stability of PROTACS remains a critical parameter that warrants further investigation to ensure the efficacy of such heterobifunctional molecules. A fundamental event governing the workings of PROTACs is the formation of a ternary complex; a tripartite assembly consisting of the POI, the E3 ligase and the PROTAC. The ternary complex is a cornerstone to the UPS mediated degradation of proteins, as it brings the POI into close proximity with the E3 ligase, thereby enabling the process of proteasomal degradation to occur.^383^^,^^384^ Factors such as the selectivity and stability of PROTACs are dependent on the ternary complex cooperativity. Although the specificity of small-molecule inhibitors is often attributed to the binary affinity of a warhead or E3 binder, PROTAC selectivity is influenced by positive cooperative effects that serve to increase target degradation.^385^^,^^386^ Cooperativity defines how the extent to which the binding of a PROTAC to one of its binding partners affects its affinity for the other. Achieving such cooperativity in PROTAC design is crucial for ensuring the prevention of off-target effects associated with the binding of unintended proteins of interest, along with ensuring efficacy in the mediation of UPS mediated protein degradation. The development of PROTACs having cooperativity proves to be difficult due to the numerous parameters that influence such a process, namely binary binding affinities, orientation and geometry of binding, kinetics, linker length and orientation and the buried surface area at the ternary interface.^387–389^ Despite significant advancements in molecular design and physicochemical properties, the efficacy of PROTACs in cellular systems remains constrained due to the presence of adaptive biological mechanisms. Advancements in TPD, facilitated by extensive research aimed at improving selectivity and potency, are hindered by cellular adaptive responses such as the upregulation of proteins with compensatory or deubiquitinating functions, as well as the emergence of mutations in ternary complex proteins that reduces the affinity of heterobifunctional molecules for the POI and degradation machinery. A fundamental component of the PROTAC mechanism is the ubiquitination of the POI for subsequent degradation in the proteasome. Even if such ubiquitination is achieved through optimizations in the PROTAC design, the presence of deubiquitinates (DUBs) can prevent degradation from occurring. The upregulation of DUBs has been documented across multiple cancer types, including examples such as USP7, USP9X, USP22, USP28, USP2, USP13, USP17, and USP3.^32^^,^^390^ These oncogenic proteins serve to hijack various signalling pathways for the prevention of apoptosis, thereby proving to be a potential challenge towards achieving TPD with PROTACs. Additionally, mutations in the components of the ternary complex can also reduce the efficacy of PROTACs, specifically mutations in the target E3 ligase and protein of interest. The long-standing issue of target protein mutations compromising small-molecule inhibitor efficacy is likewise pertinent to PROTACs, which depend on the quality of two protein-ligand interactions to enable POI binding and ubiquitination.^391–393^The emergence of mutations within E3 ligases commonly recruited by PROTACs such as CRBN and VHL, has been identified as a key determinant of resistance towards PROTAC-mediated degradation.^394^ This occurrence contrasts with the mechanism of resistance typically observed for small-molecule inhibitors, in which mutations are typically in the POI. In addition to upregulation and mutations of key proteins in the TPD process, the efflux of PROTACs contributes to the array of challenges hampering PROTAC efficacy. The active efflux of administered drugs mediated by membrane transport proteins represents a major determinant of multidrug resistance, a phenomenon that has also been implicated in the observed mechanisms of resistance to PROTACs.^395^^,^^396^ Proteomic analyses of PROTAC-resistant cancer cells have revealed that an overexpression of the drug efflux pump MDR1 (ABCB1) results in reduced intracellular levels of PROTACs, while inhibition of the MDR1 pump restores PROTAC-mediated degradation of the POI. Members of the ABC (ATP-binding cassette) transporter family are highly expressed in certain tissues, notably in human intestinal epithelial cells, which exhibit abundant expression of efflux transporters such as P-glycoprotein (P-gp, ABCB1) and the breast cancer resistance protein (BCRP, ABCG2). The quantification of PROTAC efflux in the work of Scott et al. underscores how minor structural modifications to PROTAC design results in significant efflux ratio differences, demonstrating the need for enabling diversity in PROTAC design while mitigating the challenge of drug efflux.^397^ The influence of lipophilicity on PROTAC efflux has been systematically investigated by Klein et al. using a series of pseudo-PROTAC analogs. In these studies, compounds incorporating a VHL ligand, an amide-based linker, and a surrogate warhead demonstrated that increasing lipophilicity up to approximately 4 AlogP resulted in higher efflux levels, whereas further increases in lipophilicity resulted in a decline in efflux. Collectively, these findings emphasise the importance of optimising structural features that reduce efflux susceptibility, while concurrently mitigating processes that drive efflux, such as the activity of transport pumps.

## Conclusion

Overall, PROTACs (Proteolysis Targeting Chimaeras) have emerged as a transformative and promising approach to treating various forms of cancer through facilitating the elimination of oncogenic proteins in a selective manner. Notable advancements in the optimisation of E3 ligase ligands, linker chemistry and the rational design of warheads enable the opportunity to tap into novel PROTAC designs for the degradation of a diverse range of cancer-associated proteins. Such heterobifunctional molecules also open the door to multi-target degradation strategies and tools for elucidating protein function in complex signalling networks. Prospectively, the integration of structural biology and computational tools is set to advance novel ligand and linker designs yielding PROTACs with enhanced specificity and bioavailability. The extension of PROTAC-based therapies beyond oncology, into fields such as immunology, infectious diseases and metabolic disorders are crucial for affirming the utility of this approach in targeted protein degradation. As the understanding of optimal PROTAC design deepens, these heterobifunctional molecules are anticipated to redefine approaches to drug development across therapeutic areas.

## Data Availability

Data sharing is not applicable to this article as no new data were created or analysed in this study.

## Acronyms

ACC: Acetyl-CoA carboxylase
ACSL4: Acyl-CoA Synthetase Long-Chain Family Member 4
AMPK: AMP-activated protein kinase
AR: Androgen Receptor
ATG: Autophagy-related protein family
BET: Bromodomain and extraterminal domain
BCL6: B-cell lymphoma 6
BCL-XL: B-cell lymphoma-extra-large
BiGRU: Bidirectional gated recurrent unit
BRCA: Breast Cancer Gene
cGAS-STING: cyclic GMP-AMP synthase (cGAS)-stimulator of interferon genes (STING) pathway
c-MYC: Cellular MYC transcription factor
CD36: Cluster of Differentation 36
ChIP: Chromatin immunoprecipitation
CNN: Convolutional neural network
COPI: Coatomer protein complex I
COPZ1: Coatomer protein complex I subunit zeta 1
CRBN: Cereblon
CRC: Core regulatory circuitry
DC_50_: Half maximal degradation concentration
DCAF16: DDB1-and CUL4-associated factor 16
D_max_: Maximal fraction of a target protein that is degraded
EC_50_: Half maximal effective concentration
EGFR: Epidermal Growth Factor Receptor
ENCODE: Encyclopedia of DNA elements
ERα: Oestrogen receptor alpha
ESR1: Oestrogen receptor 1
FSP1: Ferroptosis suppressor protein 1
FTH1: Ferritin heavy chain 1
FTL: Ferritin light chain
GABARAP: γ-Aminobutyric acid receptor-associated protein
GPX4: Glutathione peroxidase 4
GWAS: Genome-wide association study
HERC2: HECT and RCC1-like domain-containing protein 2
HIF-1α: Hypoxia-inducible-factor-1α
HDAC: Histone deacetylase
IC_50_: Half maximal Inhibition concentration
IMiD: Immunomodulatory drugs
-log(Pe) (PAMPA): logarithmic report of membrane permeability as determined by Parallel Artificial Membrane Permeability Assay
LC3: Microtubule-associated protein 1 light chain 3
LIP: Labile iron pool
LOOH: Lipid hydroperoxide
LOX: Lipoxygenase
LYTAC: Lysosome targeting chimaera
MLN4924: Pevonedistat; potent and selective inhibitor of NEDD8-activating enzyme (NAE).
MLP: Multilayer perceptron
MG132: Carbobenzoxy-L-leucyl-L-leucyl-L-leucinal (inhibitor of the 26S proteasome)
NCOA4: nuclear receptor coactivator 4
NOTCH1: Notch receptor 1
NSCLC: Non-Small Cell Lung Cancer
PARP: Poly (ADP-ribose) polymerase
PEG: Polylethylene glycol
POI: Protein of interest
PTM: Post-translational modification
PUFA: Polyunsaturated fatty acid
ROS: Reactive Oxygen Species
SE: Super-enhancer
SLC7A11: Solute Carrier Family 7 Member 11
SLC3A2: Solute Carrier Family 3 Member 3
SMARCB1: SWI/SNF Related BAF Chromatin Remodelling Complex Subunit B1
SMARCA-2: SWI/SNF Related BAF Chromatin Remodelling Complex Subunit ATPase 2
STAT3: Signal Transducer and Activator of Transcription 3
TAL1: T-cell acute lymphocytic leukaemia protein 1
TAX1BP1: Tax 1 binding protein 1
TBK1: TANK-binding kinase 1
TPD: Targeted protein degradation
TRPML: Transient receptor potential cation channel Mucolipin
UPS: Ubiquitin Proteasome system
VHL: Von Hippel-Lindau
5hmC: 5-hydroxymethylcytosine
6mA: N6-methyladenine
4mC: N4-methylcytosine
